# Sustainability through a gender lens: The extent to which research on UN Sustainable Development Goals includes sex and gender consideration

**DOI:** 10.1371/journal.pone.0275657

**Published:** 2022-10-07

**Authors:** Rachel Herbert, Holly J. Falk-Krzesinski, Kristy James, Andrew Plume

**Affiliations:** 1 International Center for the Study of Research, Elsevier, Amsterdam, The Netherlands; 2 Elsevier, Kidlington, Oxford, United Kingdom; 3 Elsevier Inc, New York, New York, United States of America; 4 School of Professional Studies, Northwestern University, Chicago, Illinois, United States of America; Rose-Hulman Institute of Technology / Indiana University, UNITED STATES

## Abstract

Through efforts of the Gender Summits and UN Women, it is evident that all United Nations (UN) Sustainable Development Goals (SDGs) targets must be viewed from a gender perspective to ensure that the outcomes benefit women and men equally. Our research focuses on the extent to which sex and gender topics are explicitly covered in research related to the SDGs. Expanding on previous studies, we have developed an approach to detect and visualize the volume and proportion of research publications that include explicit mention of sex and gender terms. The approach visualizes the topical coverage of the publications in the corpus of each SDG as a term map, and overlays that view with the proportion of the publications associated with sex and gender topics. We show that attention to sex and gender topics is uneven across the SDGs, and that even where overlap between an SDG and consideration of sex and gender is high, significant topical areas of relevance to the SDG have little explicit connection with sex and gender. This study lays the groundwork for the evidence-based development of a roadmap toward greater integration of sex and gender across all SDGs as well as monitoring integration progress over time.

## Introduction

In recent years, there has been growing recognition of the benefits of incorporating sex and gender analysis into research, with calls for this dimension to be considered from the research design stage [1, 2]. By doing so, research questions will be answered more comprehensively and the research itself will be more robust and reproducible [3].

It has also become evident, particularly through a report by UN Women [4] and discussions and work presented at the Gender Summits held since 2011 [5, 6], and previous research [7], that the targets for the United Nations’ (UN) Sustainable Development Goals (SDGs) [8] must be viewed from a gender perspective to ensure that gender-responsive policies and accountability processes are developed and the outcomes to achieve the goals benefit women and men equally. This is also highlighted by the UN principle of ‘Leave No One Behind’ [9]. Indeed, progress on the goals can only be achieved through action plans that incorporate this consideration [10]. The World Health Organization (WHO) has also called for more nuanced consideration of gender: a broader view of the concept [4] and a deeper understanding of the reasons behind gender differences do exist [11].

Attention to sex and gender has increased in many areas of life in recent years, but this important dimension is still often missing from published research. This is especially the case where the first and last authors of publications are not women [12], and it has been demonstrated in recent findings from an Elsevier report that women authors are less commonly last authors than men [13]. Although the significance of the first and last author position varies, in some fields, such as molecular biology, the last author position is usually reserved for the principal investigator or equivalent [14]–an author who will steer the overall research project. Taken all together, this suggests that the situation is unlikely to change without much greater attention on this issue in research institutions.

Several of the SDGs are written with a recognition of the role of sex and gender in achieving outcomes and are specified in their targets and the indicators used to measure progress. But while the UN has recognized a need for “systematic mainstreaming of a gender perspective” [15], this is not the case across all 17 goals: one UN Women report identified cases where targets that included a gender dimension were lacking that dimension in the monitoring indicator [4] and another reported insufficient data to enable comprehensive tracking of progress [16]. With SDG outcomes fixed to a 2030 target date, building an understanding of how sex and gender are embedded within the research supporting the SDGs is an immediate imperative to support evidence-based implementation of the agenda and ensure that the impacts of the SDGs in gender can be robustly assessed. To establish an understanding based on published research studies, we have developed an approach to detect and visualize the volume and proportion of research publications that include explicit mention of sex and/or gender topics. In the rest of this paper we will outline this approach and highlight some key findings.

## Methods

Expanding on previous studies that investigated gender in research from a topical perspective using the Scopus database [17], we have developed a keyword search-based approach to identify publications that explicitly include terms related to sex and/or gender topical research in the title, abstract or keywords. These publications are matched to the corpus of publications reflecting research related to each of 16 SDGs (excluding SDG 17: Partnership for the Goals) that have been defined on the basis of expert-informed Scopus keyword searches augmented with machine learning [18].

As an important point of departure from previous work, in this study we consider sex and gender together. Following the WHO’s definitions, sex “refers to the biological characteristics that define humans as male or female,” and gender “refers to the socially constructed norms, roles and relations of and among women, men, boys and girls,” as well as the “expressions and identities of women, men, boys, girls and gender-diverse people” [19]. While they are separate concepts, they are also related and the keyword search that we have developed incorporates terms that relate to both. This allows for the often interchangeable (and perhaps incorrect) use of terms relating to either or both of these concepts by publication authors.

The keyword-based approach to identify publications including terms related to sex and/or gender in the title, abstract or keywords was created in an iterative fashion as follows. We captured relevant keywords from: i) keywords from publications within the public Mendeley library “Gender in the Global Research Landscape” [20]; ii) terms used by established organizations and societies, e.g. Gender Identity Research and Education Society, UNICEF and UNESCO; iii) and terms provided by Portia Ltd, the organizer of the Gender Summits. Each keyword was tested individually in a Scopus search of publication titles, abstracts and author/index keywords for precision and recall and appropriate wildcards, Boolean and proximity operators were identified for each. Some keywords (such as ‘man’, ‘marriage’ and ‘family’) were excluded because their non-specificity resulted in decreased precision without any increase in recall. While it is true that many of those terms that appear in the final query add only incrementally to the sum total of the publications retrieved in Scopus, we were conscious not to oversimplify our query to those terms that did retrieve the majority of the results (such as the single term ‘gender’, for example). We also consider that all relevant terms are equally valuable to include and that sex and gender terms are used inconsistently and sometimes incorrectly in the literature and our keyword search deliberately conflates them in order to reflect that ambiguity.

We studied years 2015 to 2020, but for the term mapping, publications were limited to those published in 2020, as this is the most recent period for which we had a full data year of articles within Scopus when we executed the analysis, and to peer-reviewed types (i.e., articles, reviews, conference proceedings, short surveys and data articles). In selecting 2020, we are illustrating the analysis that our proposed approach could show and the insights that can be revealed. The final, selected Scopus sex and gender keyword search can be found in Table 1.

**Table 1:** The selected Scopus sex and gender keyword search.

TITLE-ABS-KEY(woman OR transgender* OR sexuality OR sexis* OR patriarch* OR neutrois OR matrimon* OR matriarch* OR maternity OR maternal* OR paternity OR paternal* OR masculin* OR intersectional* OR housewi*e* OR femini* OR {third sex} OR {men} OR *gender* OR "son preference" OR "sexual object*" OR "sex traffick*" OR "non binary" OR "human traffick*" OR "force* marriage*" OR "daughter preference" OR "child rear*" OR "sex* affect*" OR "sex* biodivers*" OR sexing OR male OR female OR childbear* OR {sexes} OR "sexual dimorph*" OR "sex* variat*" OR "sex* system*" OR "sex* select*" OR "sex* related differen*" OR "sex* ratio*" OR "sex* preselect*" OR "sex* matur*" OR "sex* identif*" OR "sex* factor*" OR "sex* extinct*" OR "sex* divers*" OR "sex* distribution*" OR "sex* disparit*" OR "sex* differen*" OR "sex* determin*" OR "sex* dependen*" OR "sex* chromosome*" OR "sex* character*" OR "sex* specific*" OR "sex* indicat*" OR "reproductive work*" OR "reproductive right*" OR "reproductive health*" OR "sex* violen*" OR "sex* harass*" OR "sex* exploit*" OR "sex* discriminat*" OR mother* OR boy OR girl OR father* OR "sex* trait*" OR "sex* health" OR "sex* behavio*r" OR daughter OR parent*) OR TITLE-ABS-KEY(biolog* w/3 sex) OR TITLE-ABS-KEY(biomark* w/5 sex) OR TITLE-ABS-KEY(sex w/5 stratif*) AND DOCTYPE(ar OR re OR cp OR sh OR dp) AND PUBYEAR IS 2020 AND NOT TITLE-ABS-KEY(*engender*)

For each of the 16 SDGs included in this study, a corpus consisting of the publications identified by queries described recently [18] was created from an analytical copy of the Scopus dataset accessed via ICSR Lab [21], snapshot dated June 1^st^ 2021. This delivered a unique publication set for each of the 16 SDGs. A second corpus consisting of the publications identified by each SDG keyword search AND the selected sex and gender keyword search was created from the same data. Publications in the first corpus (SDG-related publications) that also appear in the second corpus (sex and/or gender-related publications) were tagged as such after matching using Scopus unique publication identifiers. This tagging was used as the basis for calculating the proportion of each SDG’s publications that include those related to sex and/or gender research topics as well as for developing topical maps using VOSviewer.

VOSviewer is “a software tool used for constructing and visualizing bibliometric networks” [22]; the current version at the time of analysis (Version 1.6.18) was employed. This tool uses natural language processing and network mapping techniques to process publication data exported from Scopus for visualization and further analysis. Owing to the processing limits of VOSviewer, where SDG publication sets were too large for mapping they have been randomly down-sampled to approximately 20,000 publications; this applies to all SDG publication sets except for SDG 1, which resulted in fewer than 20,000 publications and so all publications were included because that volume falls within the processing power of VOSviewer. In VOSviewer, we applied binary counting of terms, meaning that the presence or absence of a term in a publication was used for determining the occurrence frequencies and term co-occurrence, not the number of occurrences of a term in a publication. We applied a term occurrence threshold of at least 100 occurrences for inclusion in the map across each SDG’s publication set with the exception of SDG 1, where the smaller volume of retrieved publications meant that a threshold of at least 50 publications was more appropriate; these thresholds were selected heuristically as a trade-off between comprehensiveness and readability/interpretability of the resulting maps, and other thresholds did not materially alter our observations. VOSviewer’s default setting to map 60% of the most relevant terms (based on the calculated relevance score) was selected since “terms with a high relevance score tend to represent specific topics covered by the text data, while terms with a low relevance score tend to be of a general nature and tend not to be representative of any specific topic. By excluding terms with a low relevance score, general terms are filtered out and the focus shifts to more specific and more informative terms. By default, 40% of the terms are excluded based on their relevance score” [23]. To further validate the specificity of the sex and gender keyword search, we carefully examined the results of the approach described above for SDG 5: Gender Equality. We found the expected high degree of overlap between the SDG 5 publication set and those publications within it tagged as also being identified by the sex and gender keyword search.

## Results

### Most SDGs have a low and steady proportion of publications related to sex and/or gender

The number of research publications in 2020 identified by each SDG query is shown in Table 2, along with the number and proportion of these that were also identified by the sex and gender keyword query. The variation in volume of each of the SDG’s corpus is apparent, ranging from SDG 3: Good Health and Well-being (417,443 publications) to the much smaller SDG 1: No Poverty (13,424 publications), to some extent reflecting the disparity in the number and complexity of the targets relating to each goals (e.g. 13 targets for SDG 3 versus 7 targets for SDG 1). Of particular relevance to the present work we note the relatively small number of publications returned by the SDG 5: Gender Equality query (25,601 publications). Most of the SDGs have a low proportion of publications that explicitly relate to sex and gender research topics. SDG 5: Gender Equality and SDG 3: Good Health and Well-being stand out for their high shares (95% and 62%, respectively). Among the remainder, no SDG has a share above 40% and eight are under 10%. This means that most research on SDGs does not include explicit consideration of sex and/or gender: indeed, among the full, deduplicated dataset of 1.6 million publications, 21% of publications explicitly mentioned sex or gender.

**Table 2:** Counts and proportion of SDG publications that include sex and gender keywords in 2020.

SDG No.	Sustainable Development Goal	Publications identified by SDG query	Publications ALSO identified by sex and gender keyword search	Proportion of SDG publications that include sex and gender keywords	SDG’s UN Women gender classification
5	Gender Equality	25,601	24,319	95%	Gender-sensitive
3	Good Health and Well-being	417,443	256,741	62%	Gender-sensitive
16	Peace, Justice and Strong Institutions	35,037	13,599	39%	Gender-sensitive
10	Reduced Inequalities	38,250	14,129	37%	Gender-sparse
1	No Poverty	13,424	4,142	31%	Gender-sensitive
	Quality Education	37,206	9,302	25%	Gender-sensitive
2	Zero Hunger	37,067	7,335	20%	Gender-sparse
8	Decent Work and Economic Growth	40,920	5,639	14%	Gender-sensitive
15	Life on Land	35,543	3,114	9%	Gender-blind
11	Sustainable Cities and Communities	57,878	3,992	7%	Gender-sparse
14	Life Below Water	28,146	1,882	7%	Gender-blind
6	Clean Water and Sanitation	51,057	2,349	5%	Gender-blind
12	Responsible Consumption and Production	37,391	1,533	4%	Gender-blind
13	Climate Action	42,699	1,336	3%	Gender-sparse
9	Industry, Innovation and Infrastructure	58,662	1,764	3%	Gender-blind
7	Affordable and Clean Energy	112,053	1,037	1%	Gender-blind
	**Deduplicated total**	**1,669,868**	**352,228**	**21%**	**n/a**

SDGs 1–16 with the count of publications in 2020 identified by the SDG query, and the count and proportion of publications of these also found by the sex and gender keyword search. SDGs are ranked descending by this proportion. The gender classifications are those identified within the UN Women report [4].The ranking of the SDGs by proportion of sex and/or gender topical publications does align to some extent with the classification that the report from UN Women gave to the SDG indicator framework [4]. In Figure 2.1 of that report, each SDG was classified as either “gender-sensitive”, “gender-sparse” or “gender-blind” to reflect the extent to which the SDG indicators are gender-specific; this classification appears in the final column of Table 2. While the UN Women classification addressed gender (not sex), it is nonetheless clear that the “gender-sensitive” SDGs tend to have the amongst the higher proportions of sex and gender publications, and the “gender-sparse” and “gender-blind” SDGs tend to have the lowest shares of sex and gender publications. However, SDG 10: Reduced Inequalities appears to have a higher than expected proportion of publications on sex and gender given its “gender-sparse” indication, and conversely the “gender-sensitive” SDG 8: Decent Work and Economic Growth has a relatively low proportion. It is important to note that some of the SDGs with low proportions of publications explicitly mentioning sex and gender and which are “gender-sparse” or “gender-blind” under the UN Women classification nevertheless do deal with targets and topics of high sex and gender relevance. For instance, SDG 7: Affordable and Clean Energy indicates a need to transition much of the world’s population towards “clean cooking fuels and technologies” which is highly gender-relevant; such nuanced views are more clearly resolved by disaggregating these summary statistics into thematic topics through the use of a term mapping approach.

The results for the single year snapshot (2020) serve as the basis for the next steps in our approach to depict the extent to which sex or gender is considered in the research for each of the 16 SDGs. However, we also wanted to understand how the proportions are changing over time. The SDGs were established in 2015 [8] and so we also calculated the proportions of each SDG’s publications that consider sex or gender for the years 2015 to 2020 (Table 3). Although there are publishing lags that may mean that some 2015 papers were written prior to the SDGs being established, we include all years for completeness. Table 3 shows just the proportions for each year; in the online supplementary information section of this paper, a full table including all the publication counts of all publications relating to each SDG and the subset of which relate to sex or gender can be found (Table 1).

**Table 3:** Proportion of SDG publications that include sex and gender keywords, 2015–2020.

		Proportion of SDG publications that include sex and gender keywords					
SDG No.	Sustainable Development Goal	2015	2016	2017	2018	2019	2020
1	No Poverty	31%	31%	31%	32%	33%	31%
2	Zero Hunger	20%	20%	20%	20%	20%	20%
3	Good Health and Well-being	64%	63%	64%	64%	64%	62%
4	Quality Education	24%	24%	26%	26%	26%	25%
5	Gender Equality	94%	94%	95%	95%	95%	95%
6	Clean Water and Sanitation	5%	5%	5%	5%	5%	5%
7	Affordable and Clean Energy	1%	1%	1%	1%	1%	1%
8	Decent Work and Economic Growth	15%	14%	14%	14%	14%	14%
9	Industry, Innovation and Infrastructure	3%	3%	3%	3%	3%	3%
10	Reduced Inequalities	34%	34%	36%	37%	38%	37%
11	Sustainable Cities and Communities	7%	7%	7%	7%	7%	7%
12	Responsible Consumption and Production	4%	3%	4%	4%	4%	4%
13	Climate Action	3%	3%	3%	3%	3%	3%
14	Life Below Water	7%	8%	8%	7%	7%	7%
15	Life on Land	10%	9%	9%	9%	9%	9%
16	Peace, Justice and Strong Institutions	37%	37%	38%	39%	39%	39%
null	Deduplicated total	20%	20%	20%	21%	20%	21%

The results for 2015–2020 (Table 3) show very little change in the proportions of publications that consider sex or gender. At most, the proportion of SDG 3: Good Health and Well-being publications referencing sex and gender dropped by 2.0 percentage points between 2019 and 2020. If this change did prove to be meaningful in the longer term, we might speculate that this may be linked to an influx of COVID research in 2020 which did not explicitly make reference to sex or gender, particularly in the early stages of the pandemic response. The only other notable change is for SDG 16: Peace, Justice and Strong Institutions which had a slight increase in the proportion of publications that consider sex or gender, increasing by 1.5 percentage points across the six-year period. For the rest of the SDGs, the changes in proportions fluctuate slightly but remain steady.

SDGs 1–16 with the proportion of publications found by each SDG query and by the sex and gender keyword search for publication years 2015–2020. The gender classifications are those identified within the UN Women report [4]. In the supplementary information accompanying this article, an extended version of this table (Table 2) is available which includes the raw counts of the publications associated with each SDG, and the raw counts of those publications also associated with sex and/or gender.

For the most current view using the most recent full year at the time of conducting the research, we selected 2020 to demonstrate our approach to mapping for insights. Though we do acknowledge that 2020 was not a typical year owing to the onset of the COVID-19 pandemic, we nonetheless believe that small perturbations in the research output and sex and gender focus that year will be minimal and only likely to appear in SDG 3: Good Health and Well-being.

The term mapping approach allows us to examine topical clusters in the corpus of research relevant to each SDG, and to overlay this with a view on those clusters which explicitly include terms related to sex and/or gender, which represent publications on sex and gender research topics. The advantage of this approach is that it allows the terms used by the authors of these publications themselves to be examined, rather than relying on any external classification scheme that which may not be sufficiently fine-grained to detect niche topics or emerging research fronts. In the following sections we will present a selection of these SDG maps. In the online S1 File, the maps for all 16 SDGs are presented.

### SDG 5: Gender equality has the expected high coverage of sex and gender relevant research

**Fig 1:**
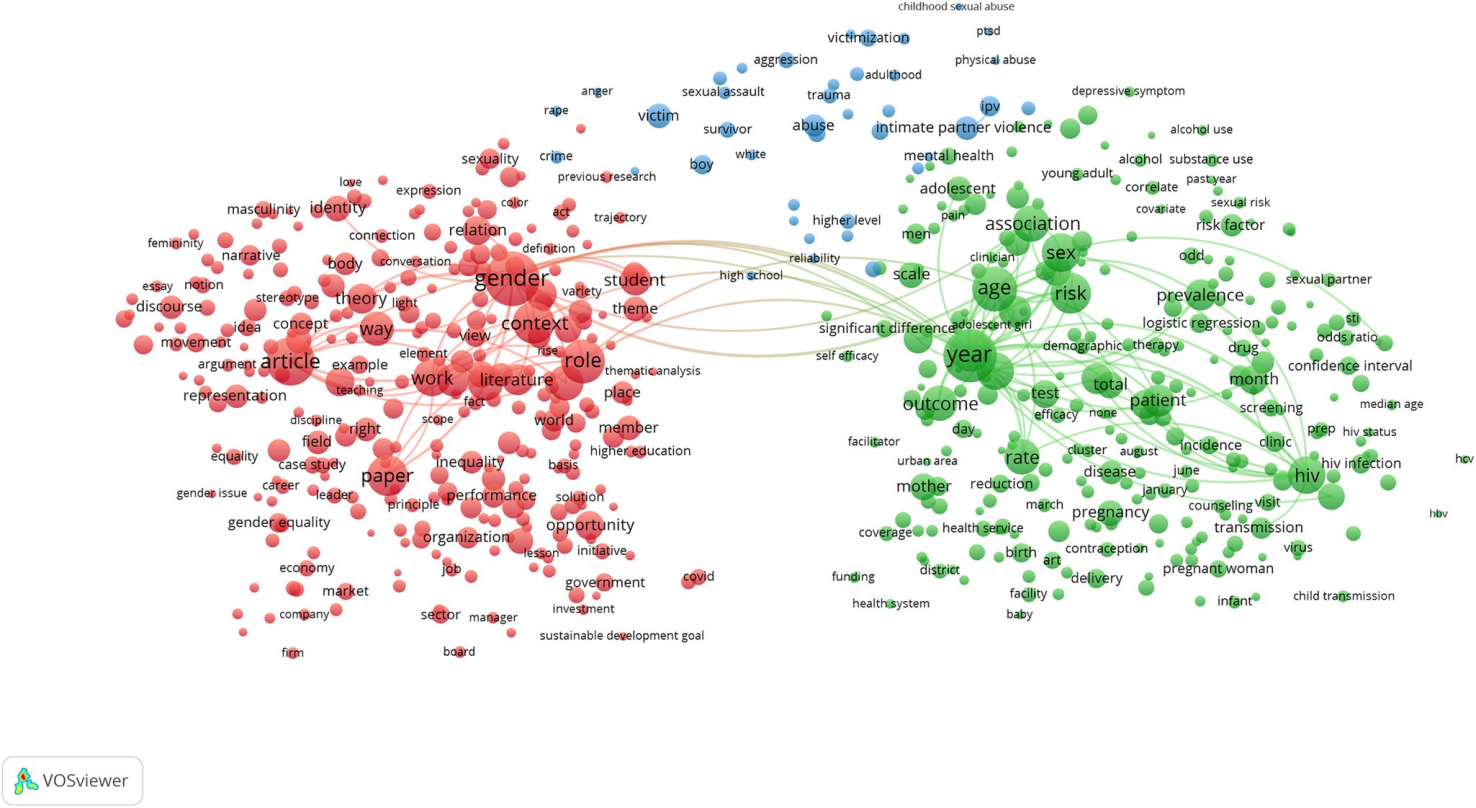
SDG 5: Gender equality term network map. Binary counting (present/absent, not count of occurrences) was applied to terms in titles and abstracts of 25,601 publications in 2020, and those with at least 100 occurrences were mapped using VOSviewer. Node size indicates count of occurrences, and node proximity reflects frequency of co-occurrence (nodes close together co-occur more frequently than nodes far apart). In this network visualization, the colors indicate topical clusters.

**Fig 2:**
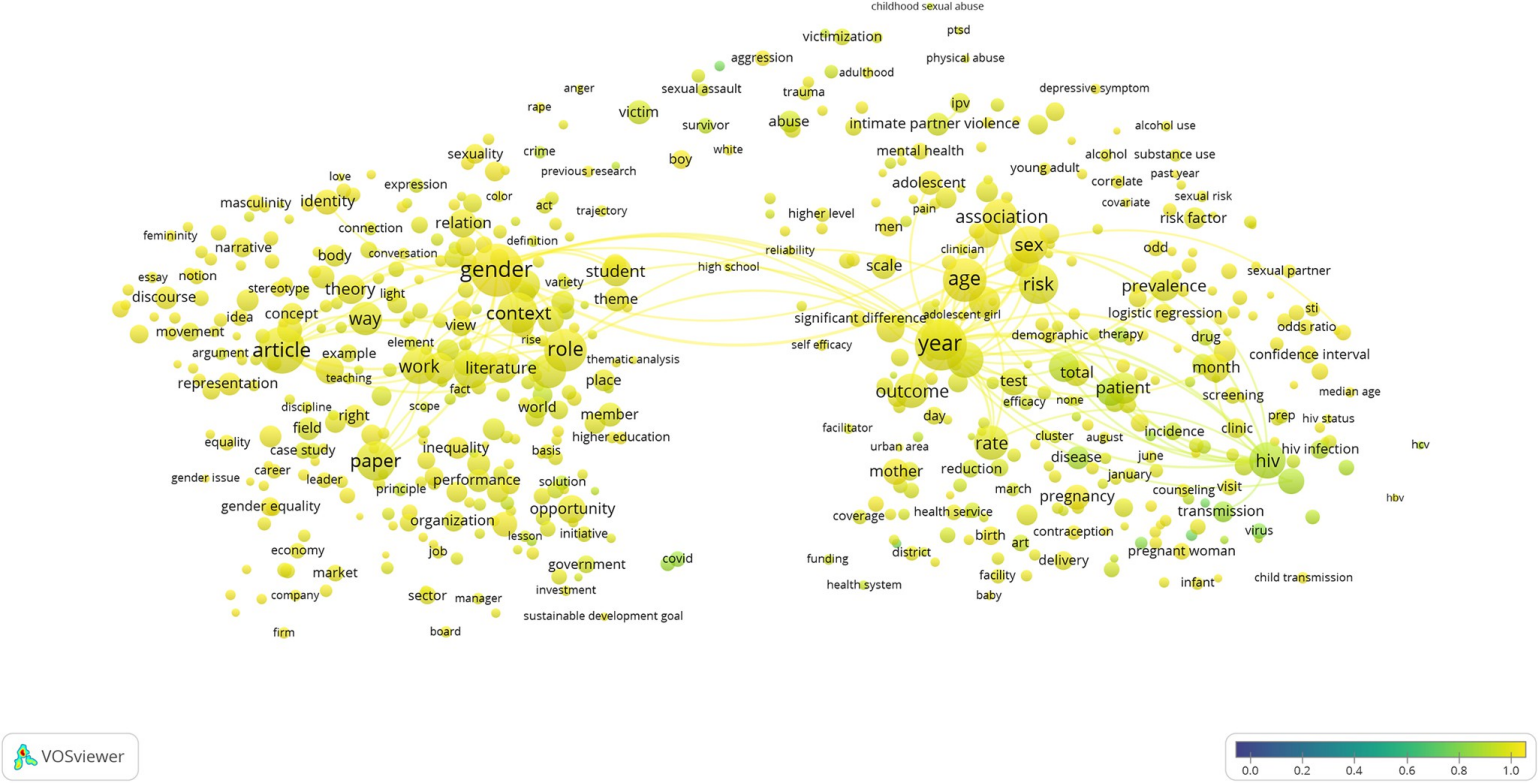
SDG 5: Gender equality term overlay map. Binary counting (present/absent, not count of occurrences) was applied to terms in titles and abstracts of 25,601 publications in 2020, and those with at least 100 occurrences were mapped using VOSviewer. Node size indicates count of occurrences, and node proximity reflects frequency of co-occurrence (nodes close together co-occur more frequently than nodes far apart). In this overlay visualization, the color scale indicates the proportion of publications associated with the mapped terms that were also identified by the sex and gender keyword search: blue nodes indicate terms with relatively low consideration of sex and/or gender; yellow terms indicate terms with relatively high consideration of sex and/or gender.

The term map created for SDG 5: Gender Equality is shown in Fig 1, and the topical clusters clearly reveal the diversity of research relevant to this SDG’s targets and indicators. Major clusters of terms relating to gender in health on the right side of the map and gender in social policy on the left are bridged by a cluster relating to the medical and policy literature around sexual abuse and exploitation appear at the top of the map. Fig 2 illustrates the high degree of overlap (95% of publications, as shown in Table 2) between the SDG publication set and those publications within it tagged as also being identified by the sex and gender keyword search, i.e. those that also cover sex and gender research topics. None of the terms visible in the map at this scale are associated with a low proportion of sex and gender tagged publications.

### Sex and gender relevant research is unevenly spread across SDG 3: Good Health and Well-being

**Fig 3:**
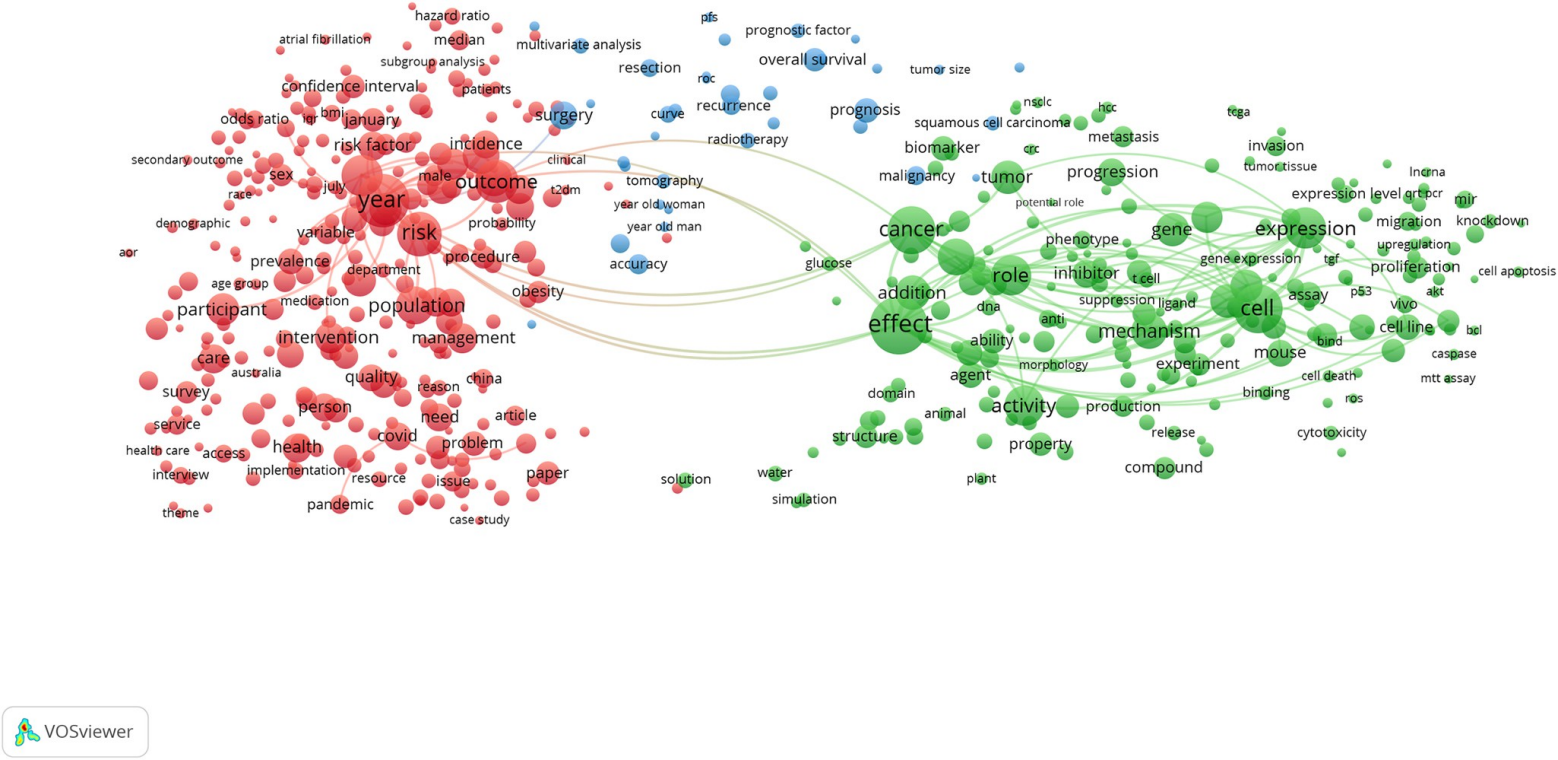
SDG 3: Good health and well-being term network map. Binary counting (present/absent, not count of occurrences) was applied to terms in titles and abstracts of 19,983 publications in 2020 (sampled from 417,443 in total), and those with at least 100 occurrences were mapped using VOSviewer. Node size indicates count of occurrences, and node proximity reflects frequency of co-occurrence (nodes close together co-occur more frequently than nodes far apart). In this network visualization, the colors indicate topical clusters.

Given the importance of human health to societies around the world, and the volume of funding made available to address research priorities ranging from formulating evidence-based public health policies to understanding the human genome, the SDG 3: Good Health and Well-being keyword search retrieves an order of magnitude more publications than most other SDG queries. The variation in volume of each of the SDG’s corpus is apparent, ranging from SDG 3: Good Health and Well-being (417,443 publications) to the much smaller SDG 1: No Poverty (13,424 publications), to some extent reflecting the disparity in the number and complexity of the targets relating to each goals (e.g. 13 targets for SDG 3. To work within the processing capability of VOSviewer, a random sample of approximately 20,000 publications was used to create the map shown in Fig 3, which shows the topical clusters relevant to this SDG’s targets and indicators. Topics related to health evidence, governance and policy appear in red on the left side of the map, while the underlying biology of human illness and disease appear in green on the right. Bridging these two larger topical domains is a smaller set of terms in blue, relating most closely to surgical interventions in cancer treatment. Of particular interest are the terms related to the COVID-19 pandemic at the bottom on the left cluster in this map such as ‘covid’, ‘pandemic’, ‘coronavirus’ and related viral designations.

**Fig 4:**
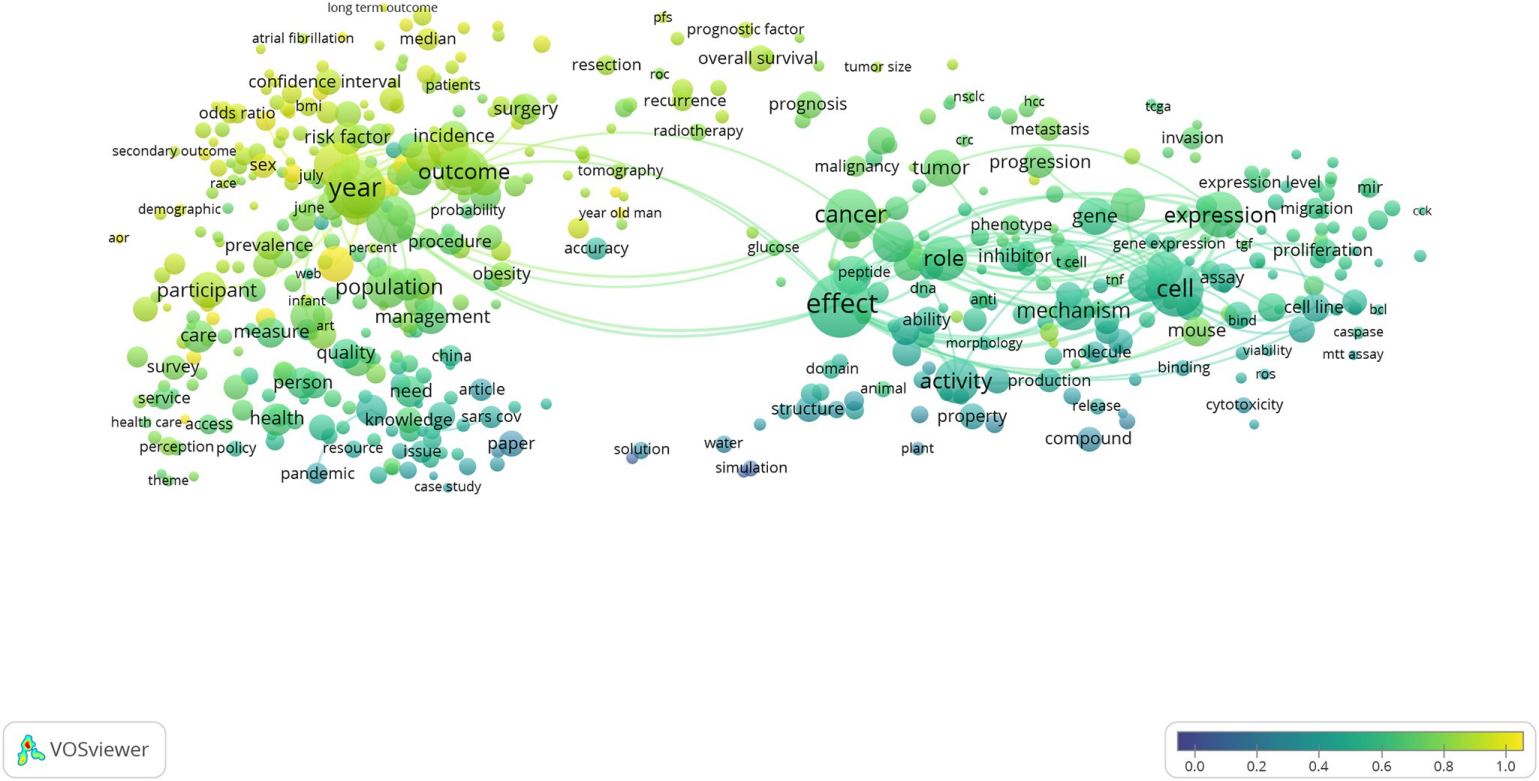
SDG 3: Good Health and Well-being term overlay map. Binary counting (present/absent, not count of occurrences) was applied to terms in titles and abstracts of 19,983 publications in 2020 (sampled from 417,443 in total), and those with at least 100 occurrences were mapped using VOSviewer. Node size indicates count of occurrences, and node proximity reflects frequency of co-occurrence (nodes close together co-occur more frequently than nodes far apart). In this overlay visualization, the color scale indicates the proportion of publications associated with the mapped terms that were also identified by the sex and gender keyword search: blue nodes indicate terms with relatively low consideration of sex and/or gender; yellow terms indicate terms with relatively high consideration of sex and/or gender.

Acknowledging the “gender-sensitive” nature of this SDG [4] and the high proportion of publications that are also identified by the sex and gender keyword search (62% of publications per Table 2), the strong overlap of sex and gender in this SDG shown in Fig 4 is to be expected. What is perhaps surprising is that the terms in this map associated with publications that do not explicitly mention sex and/or gender terms are largely in the topical clusters relating to fundamental biology (the cluster on the right side of the map) but also those terms on the left of the map that relate specifically to the COVID-19 pandemic. This is despite calls for sex and gender to be incorporated and reported in research across the spectrum of human health [3, 24–26]. Such calls recognise that our understanding of the underlying causes of poor health and effective prevention and treatment requires attention to sex and/or gender disaggregation in studies in the published research literature. In the case of the COVID-19 pandemic-related terms, this apparent under-acknowledgement of the role of sex & gender is despite early evidence that COVID-19 patient outcomes are different for men and women [27].

### Sex and gender aspects of SDG 4: Quality education are explicit in education practice but not education policy research

**Fig 5:**
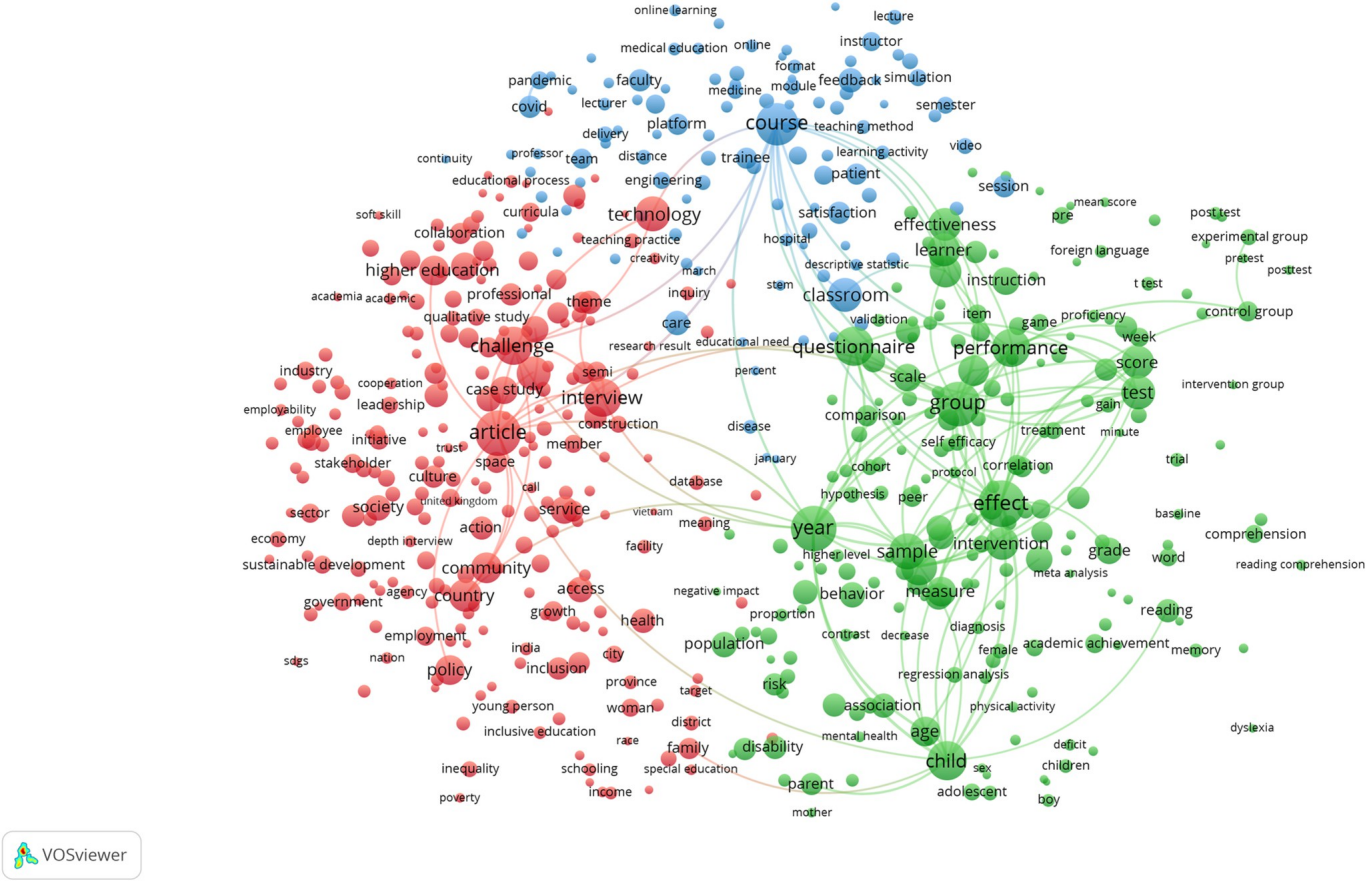
SDG 4: Quality education term network map. Binary counting (present/absent, not count of occurrences) was applied to terms in titles and abstracts of 20,030 publications in 2020 (sampled from 37,206 in total), and those with at least 100 occurrences were mapped using VOSviewer. Node size indicates count of occurrences, and node proximity reflects frequency of co-occurrence (nodes close together co-occur more frequently than nodes far apart). In this network visualization, the colors indicate topical clusters.

Pedagogy is the theory and practice of teaching and learning, and research relevant to this field is represented in the map for SDG 4: Quality Education shown in Fig 5 by four topical clusters. On the right of the map, the green cluster represents terms dealing primarily with educational settings from early years (preschool and kindergarten) through to high school (secondary education); on the left, the focus is on tertiary education as well as vocational education and labour force outcomes. The blue cluster at the top of the map deals with issues relating to medical education.

**Fig 6:**
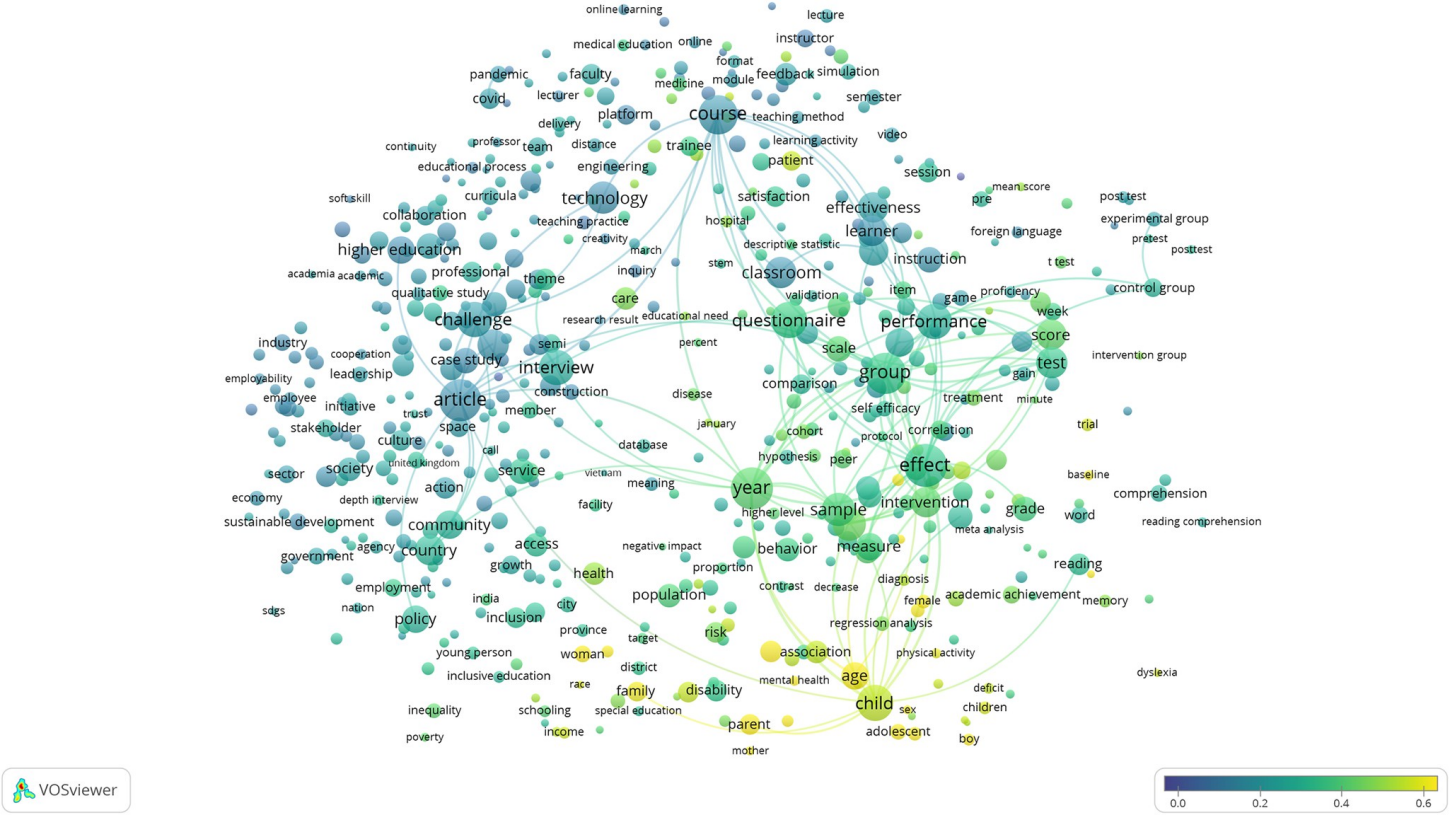
SDG 4: Quality education term overlay map. Binary counting (present/absent, not count of occurrences) was applied to terms in titles and abstracts of 20,030 publications in 2020 (sampled from 37,206 in total), and those with at least 100 occurrences were mapped using VOSviewer. Node size indicates count of occurrences, and node proximity reflects frequency of co-occurrence (nodes close together co-occur more frequently than nodes far apart). In this overlay visualization, the color scale indicates the proportion of publications associated with the mapped terms that were also identified by the sex and gender keyword search: blue nodes indicate terms with relatively low consideration of sex and/or gender; yellow terms indicate terms with relatively high consideration of sex and/or gender.

SDG 4 is classed as “gender-sensitive” by the UN Women report [4] and for many years it has been known that formative educational experiences and educational attainment are different for boys and girls [28]. However, it has also become clear more recently that teacher (professor) gender in higher education affects attainment and outcomes for women but not for men [29]. However, Fig 6 appears to reflect sex and gender elements are included in publications addressing topics around early years, junior and senior school education (and to a lesser extent in medical education) but is almost absent from those parts of the map dealing with higher education or the effectiveness of classroom teaching.

### SDG 13: Climate action has very low coverage of sex and gender relevant research

**Fig 7:**
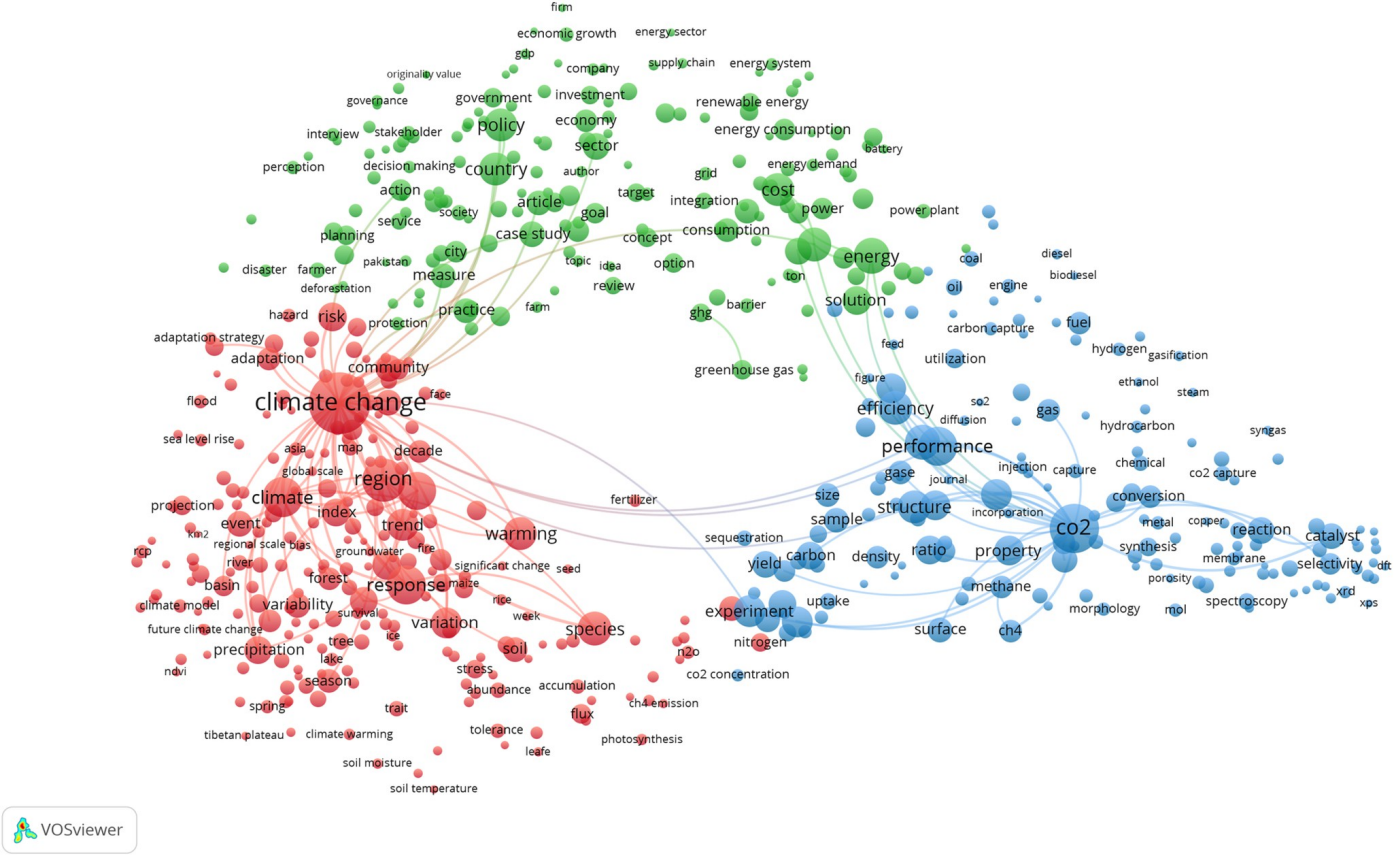
SDG 13: Climate action term map. Binary counting (present/absent, not count of occurrences) was applied to terms in titles and abstracts of 20,030 publications in 2020 (sampled from 42,699 in total), and those with at least 100 occurrences were mapped using VOSviewer. Node size indicates count of occurrences, and node proximity reflects frequency of co-occurrence (nodes close together co-occur more frequently than nodes far apart). In this network visualization, colors indicate topical clusters.

The final map we examine in detail here is for SDG 13: Climate Action, and Fig 7 illustrates the breadth of topics that this global challenge encompasses. The red cluster in the bottom left covers climate change risk, response and adaptation, while the blue cluster is focussed on carbon, especially carbon capture and storage (perhaps unsurprising given the policy emphasis on carbon sequestration [30]). Linking these is the green cluster, dealing primarily with climate and energy governance and policy.

**Fig 8:**
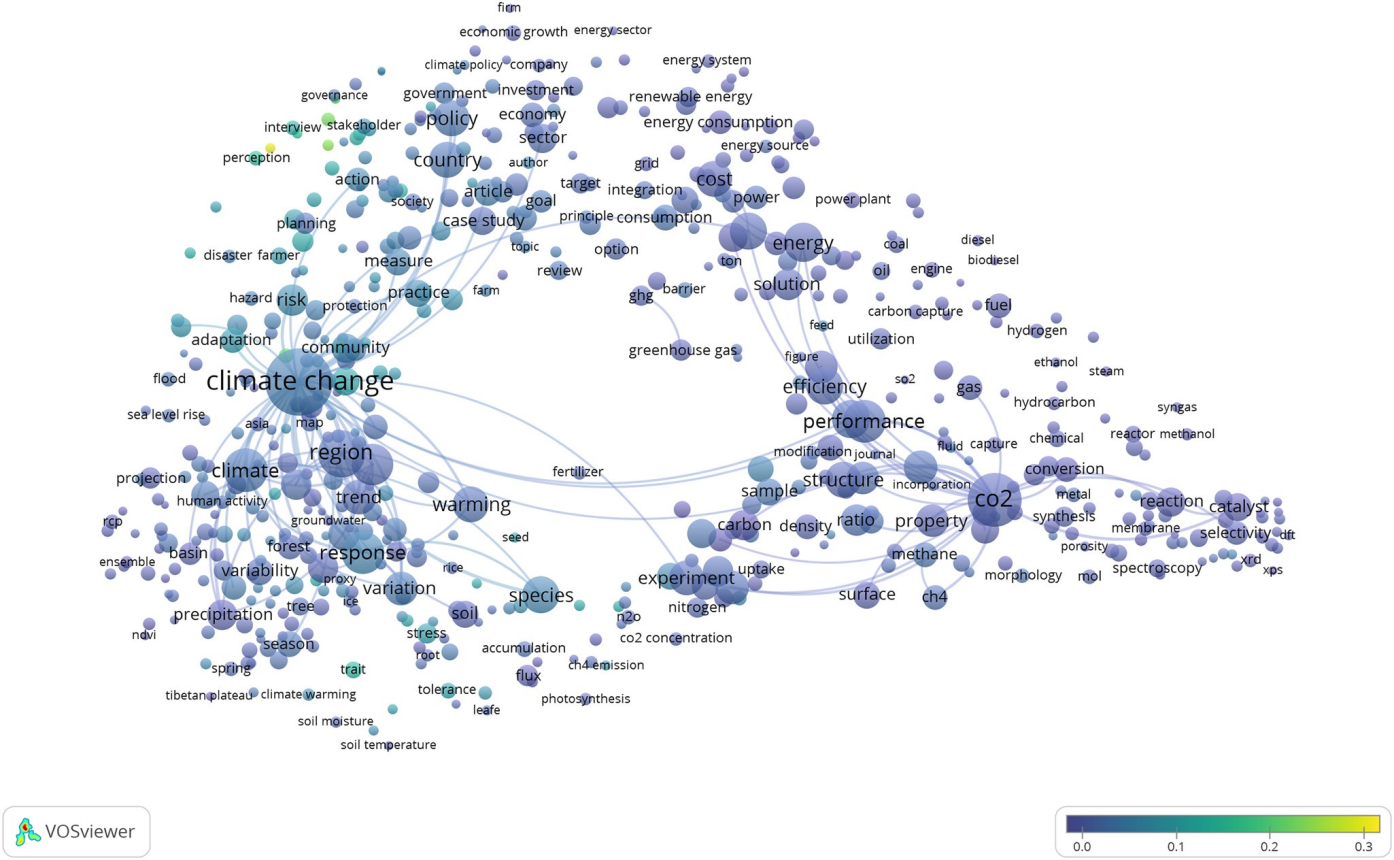
SDG 13: Climate action term map. Binary counting (present/absent, not count of occurrences) was applied to terms in titles and abstracts of 20,030 publications in 2020 (sampled from 42,699 in total), and those with at least 100 occurrences were mapped using VOSviewer. Node size indicates count of occurrences, and node proximity reflects frequency of co-occurrence (nodes close together co-occur more frequently than nodes far apart). In this overlay visualization, the color scale indicates the proportion of publications associated with the mapped terms that were also identified by the sex and gender keyword search: blue nodes indicate terms with relatively low consideration of sex and/or gender; yellow terms indicate terms with relatively high consideration of sex and/or gender.

SDG 13 has just a 3% overlap of publications found by both the SDG query and the sex and gender keyword query (and is considered “gender-sparse” according to the UN Women classification [4]). As Fig 8 makes clear, very few terms in this map are associated with a relatively high proportion of sex and gender tagged publications, and these are related mainly to perceptions of and adaptation to climate change. This seems appropriate, since evidence is building that women and men experience climate change effects differently, partly as a result of the intersection between gender, poverty and political engagement [31–35].

## Discussion

The approach described here offers a fresh perspective on both the UN SDGs and sex and gender consideration in SDG research by visualizing the topical coverage of the publications in the corpus of each SDG as a term map, and then overlaying that view with the proportion of the publications associated with each term that also explicitly include sex and/or gender terms. In establishing this approach, we want to emphasize that this is not an end-point in of itself: as research evolves and the terms used by authors to describe their work in their publications evolve too, we understand that these keyword queries will need to be revised and updated, perhaps even extended or narrowed as policy and research priorities shift and change.

What we have been able to show with great clarity even with a single year (2020) snapshot is that consideration of sex and gender is uneven across the SDGs (Table 2), and that even where overlap between the SDG and sex and gender research corpus is high as in SDG 5: Gender Equality, significant topical areas of relevance to the SDG do not consider sex and/or gender (Figs 1 and 2). Furthermore, we have demonstrated that the proportion of publications that consider sex and/or gender is quite steady over time, despite increasing calls for this consideration and even as the SDGs matured. With this, we have demonstrated that there is progress to be made if we are to ensure that women and men benefit equally from achievements that stem from the UN SDGs. However, we acknowledge that the formulation of the SDG goals and targets was not necessarily conducted with reference to sex and gender considerations and that it must not be necessarily expected that the research community has responded to the design of the UN SDGs with targeted sex and gender relevant research.

Importantly, this study lays the groundwork for the evidence-based development of a roadmap toward greater integration of sex and/or gender across research in every SDG, as well as an approach to evaluate change across each SDG over time. Our approach could be used to inform future reports by UN Women [4] to provide a rigorous evidence base to support the inclusion of sex and gender in the formulation of fresh sustainability goals and targets.

Fresh maps and tables can be created each year to monitor sex and/or gender integration progress as we move toward the 2030 SDG target goal. Furthermore, this approach could be adapted to investigate the extent to which animal and human subject studies in SDG research clusters incorporate sex and/or gender disaggregated analyses (also referred to as sex and gender-based analysis, SGBA), drawing on methodology developed for the 2018 She Figures report [36] and included within a study examining the sex and gender-based analysis of Alzheimer’s Disease research studies [37].

## Supporting information

S1 File (DOCX)

## References

[1] Heidari S, Babor TF, De Castro P, Curno M. Sex and gender equity in research: Rationale for the SAGER guidelines and recommended use. Res Integr Peer Rev. 2016;1. doi: 10.1186/s41073-016-0007-6 29451543 PMC5793986

[2] ICMJE. Recommendations for the conduct, reporting, editing, and publication of scholarly work in medical journals. 2019 [cited 2021 Dec 23]. http://www.icmje.org/icmje-recommendations.pdf 25558501

[3] Tannenbaum C, Ellis RP, Eyssel F, Zou J, Schiebinger L. Sex and gender analysis improves science and engineering. Nature. 2019;575;137–146. doi: 10.1038/s41586-019-1657-6 31695204

[4] UN Women. Turning promises into action: Gender equality in the 2030 agenda for sustainable development. 2018 [cited 2021 Dec 21]. https://www.unwomen.org/en/digital-library/publications/2018/2/gender-equality-in-the-2030-agenda-for-sustainable-development-2018

[5] Gender Summits [Internet]. GS10 Tokyo Recommendation: BRIDGE; c2016 [cited 2021 Dec 21]. https://gender-summit.com/tokyo-recommendation-bridge.

[6] Lee H, Pollitzer E. The Role of Gender-based Innovations for the UN Sustainable Development Goals. 2016 [cited 2021 Dec 21]. https://gendersummit.com/images/GS6Docs/SDG_Report_FINAL.Jan13.pdf

[7] Hepp P, Somerville C, Borisch B. Accelerating the United Nation’s 2030 Global Agenda: Why prioritization of the gender goal is essential. Glob Policy. 2019;10(4);677–685. doi: 10.1111/1758-5899.12721

[8] United Nations [Internet]. The 17 Goals; c2021 [cited 2021 Dec 21]. https://sdgs.un.org/goals

[9] Leave No One Behind [Internet]. UN Sustainable Development Group [cited 2022 Aug 31]. https://unsdg.un.org/2030-agenda/universal-values/leave-no-one-behind

[10] Lee H, Pollitzer E. Gender in science and innovation as component of inclusive socioeconomic growth. 2016 [cited 2021 Dec 21]. https://gendersummit.com/images/GS6Docs/SDG_Report_FINAL.Jan13.pdf

[11] Manandhar M, Hawkes S, Buse K, Nosrati E, Magar V. Gender, health and the 2030 agenda for sustainable development. Bull World Health Organ. 2018;96(9);644–653. doi: 10.2471/BLT.18.211607 30262946 PMC6154065

[12] Sugimoto CR, Ahn Y-Y, Smith E, Macaluso B, Larivière V. Factors affecting sex-related reporting in medical research: a cross-disciplinary bibliometric analysis. Lancet. 2019;393(10171);550–559. doi: 10.1016/S0140-6736(18)32995-7 30739690

[13] Elsevier. The researcher journey through a gender lens. 2020 [cited 2021 Dec 21]. https://www.elsevier.com/connect/gender-report

[14] West JD, Jacquet J, King MM, Correll SJ, Bergstrom CT. The Role of Gender in Scholarly Authorship. PLoS ONE 2013;8;7;e66212. doi: 10.1371/journal.pone.0066212 23894278 PMC3718784

[15] United Nations. Transforming our world: The 2030 agenda for sustainable development. 2015 [cited 2021 Dec 21]. https://www.un.org/ga/search/view_doc.asp?symbol=A/RES/70/1&Lang=E

[16] UN Women. Progress on the Sustainable Development Goals: The gender snapshot 2021. 2021 [cited 2021 Dec 21]. https://www.unwomen.org/en/digital-library/publications/2021/09/progress-on-the-sustainable-development-goals-the-gender-snapshot-2021#view

[17] Elsevier. Gender in the global research landscape. 2017 [cited 2021 Dec 21]. https://www.elsevier.com/researchintelligence/campaigns/gender-17

[18] Rivest M, Kashnitsky Y, Bédard-Vallée A, Campbell D, Khayat P, Labrosse I, et al. Improving the Scopus and Aurora queries to identify research that supports the United Nations’ Sustainable Development Goals (SDGs) 2021. Mendeley Data. 2021 Aug 26 [cited 2021 Dec 21]. https://elsevier.digitalcommonsdata.com/datasets/9sxdykm8s4/4 doi: 10.17632/9sxdykm8s4.4

[19] World Health Organization. World health statistics overview 2019. 2019 [cited 2021 Dec 21]. https://apps.who.int/iris/bitstream/handle/10665/311696/WHO-DAD-2019.1-eng.pdf?ua=1

[20] Elsevier [Internet]. Gender in the Global Research Landscape Mendeley Group; c2017 [cited 2021 Dec 21]. https://www.mendeley.com/community/gender-in-the-global-research-landscape/

[21] International Center for the Study of Research [Internet]. ICSR Lab, c2020 [cited 2021 Dec 21]. https://www.elsevier.com/icsr/icsrlab

[22] CWTS [Internet]. Welcome to VOSviewer; c2021 [cited 2021 Dec 21]. https://www.vosviewer.com

[23] CWTS [Internet]. VOSviewer Manual for version 1.6.17; c2021 [cited 2022 Aug 31]. https://www.vosviewer.com/getting-started#vosviewer-manual

[24] Hawkes S, Buse K. Gender and global health: Evidence, policy, and inconvenient truths. Lancet. 2013;381;1783–1787. doi: 10.1016/S0140-6736(13)60253-6 23683645

[25] Magar V. Gender, health and the Sustainable Development Goals. Bull World Health Organ. 2015;93(11);743. doi: 10.2471/BLT.15.165027 26549898 PMC4622165

[26] Clayton J A, Tannenbaum C. Reporting sex, gender, or both in clinical research? JAMA. 2016;316(18);1863–1864. doi: 10.1001/jama.2016.16405 27802482

[27] Gebhard C, Regitz-Zagrosek V, Klein S, Morgan R, Neuhauser HK. Impact of sex and gender on COVID-19 outcomes in Europe. Biol Sex Differ. 2020;11. doi: 10.1186/s13293-020-00304-9 32450906 PMC7247289

[28] Buchmann C, DiPrete TA, McDaniel A. Gender equalities in education. Annu Rev Sociol. 2008;34;319–337. doi: 10.1146/annurev.soc.34.040507.134719

[29] Carrell SE, Page ME, West JE. Sex and science: How professor gender perpetuates the gender gap. Q J Econ. 2010;125(3);1101–1144. doi: 10.1162/qjec.2010.125.3.1101

[30] Schenuit F, Colvin R, Fridahl M, McMullin B, Reisinger A, Sanchez DL, et al, Wreford A and Geden O (2021) Carbon Dioxide Removal Policy in the Making: Assessing Developments in 9 OECD Cases. Front. Clim. 3:638805. doi: 10.3389/fclim.2021.638805

[31] UN Climate Change. Differentiated impacts of climate change on women and men; the integration of gender considerations in climate policies, plans and actions; and progress in enhancing gender balance in national climate delegations. 2019 [cited 2021 Dec 21]. https://unfccc.int/sites/default/files/resource/sbi2019_inf8.pdf

[32] UN Climate Change. Introduction to gender and climate change. 2020 [cited 2021 Dec 21]. https://unfccc.int/gender

[33] UN Climate Change. The Enhanced Lima Work Programme on Gender. 2020 [cited 2021 Dec 21]. https://unfccc.int/topics/gender/workstreams/the-enhanced-lima-work-programme-on-gender

[34] International Union for Conservation of Nature [Internet]. Gender and climate change; c2020 [cited 2021 Dec 21]. https://www.iucn.org/resources/issues-briefs/gender-and-climate-change

[35] Fosado Centeno E. The socio-political construction of climate change: Looking for paths to sustainability and gender justice. Sustainability. 2020;12(8);3382. doi: 10.3390/su12083382

[36] European Commission. She Figures. 2018 [cited 2021 Dec 21].. https://publications.europa.eu/en/publication-detail/-/publication/9540ffa1-4478-11e9-a8ed-01aa75ed71a1/language-en

[37] Elsevier. Alzheimer’s disease research insights: Impacts, trends, opportunities. 2019 [cited 2021 Dec 21]. https://www.elsevier.com/research-intelligence/resource-library/alzheimers-disease-researchinsights

