# Supplementary material for: Sustainability through a gender lens: The extent to which research on UN Sustainable Development Goals includes sex and gender consideration
Source: PLoS One. 2022 Oct 7;17(10):e0275657. doi: 10.1371/journal.pone.0275657 (PMC9543628; doi:10.1371/journal.pone.0275657)
Supplement: S1 File — (DOCX) [file pone.0275657.s001.docx]

# **Supplementary Material**

Article title: Sustainability through a gender lens: The extent to which research on UN Sustainable Development Goals includes sex and gender consideration

Authors: Rachel Herbert, Holly J. Falk-Krzesinski, Kristy James, and Andrew Plume

**The following Supporting Information is available for this article:**

Supplementary text

Table S1

Figures S1 to S32

**Data & Methodology**

**Data source**

Throughout this study, we used data sources from an analytical copy of the Scopus dataset accessed via ICSR Lab [1], snapshot dated June 1st 2021.

**Methodology: identifying the corpora**

***Identifying sex and/or gender related publications***

A keyword search was developed to identify publications that explicitly include terms related to sex and/or gender topical research. We took the following iterative approach to build the keyword search:

1. Relevant keywords captured from:
   1. Keywords from publications within the public Mendeley library “Gender in the Global Research Landscape” [3]
   2. Terms used by established organizations and societies, e.g. Gender Identity Research and Education Society, UNICEF and UNESCO, e.g. [4]
   3. Terms provide by Portia Ltd, the organizer of the Gender Summits
2. Each keyword was tested individually in a Scopus search of publication titles, abstracts and author/index keywords for precision and recall. The appropriate wildcards, Boolean and proximity indicators were identified for each one. Some keywords (such as ‘man’, ‘marriage’ and ‘family’) were excluded because their non-specificity resulted in decreased precision without any increase in recall.

The keyword search (**Table 1**) was applied to terms in the title, abstract or keywords of publications to identify the corpus of sex and/or gender related research.

As terminology evolves, the authors recommend that the development steps be repeated periodically when re-using this approach to ensure that precision and recall remains high.

**SI Table 1. The selected Scopus sex and gender keyword search.**

| TITLE-ABS-KEY(woman OR transgender* OR sexuality OR sexis* OR patriarch* OR neutrois OR matrimon* OR matriarch* OR maternity OR maternal* OR paternity OR paternal* OR masculin* OR intersectional* OR housewi*e* OR femini* OR {third sex} OR {men} OR *gender* OR "son preference" OR "sexual object*" OR "sex traffick*" OR "non binary" OR "human traffick*" OR "force* marriage*" OR "daughter preference" OR "child rear*" OR "sex* affect*" OR "sex* biodivers*" OR sexing OR male OR female OR childbear* OR {sexes} OR "sexual dimorph*" OR "sex* variat*" OR "sex* system*" OR "sex* select*" OR "sex* related differen*" OR "sex* ratio*" OR "sex* preselect*" OR "sex* matur*" OR "sex* identif*" OR "sex* factor*" OR "sex* extinct*" OR "sex* divers*" OR "sex* distribution*" OR "sex* disparit*" OR "sex* differen*" OR "sex* determin*" OR "sex* dependen*" OR "sex* chromosome*" OR "sex* character*" OR "sex* specific*" OR "sex* indicat*" OR "reproductive work*" OR "reproductive right*" OR "reproductive health*" OR "sex* violen*" OR "sex* harass*" OR "sex* exploit*" OR "sex* discriminat*" OR mother* OR boy OR girl OR father* OR "sex* trait*" OR "sex* health" OR "sex* behavio*r" OR daughter OR parent*) OR TITLE-ABS-KEY(biolog* w/3 sex) OR TITLE-ABS-KEY(biomark* w/5 sex) OR TITLE-ABS-KEY(sex w/5 stratif*) AND DOCTYPE(ar OR re OR cp OR sh OR dp) AND PUBYEAR IS 2020 AND NOT TITLE-ABS-KEY(*engender*) |
| --- |

We limited publications to those published between 2015 and 2020, inclusive.

***Identifying sex and/or gender related publications among SDG research***

*Identifying the corpora:* The publications referencing sex and/or gender were matched to the corpus of publications reflecting research related to each of 16 SDGs (excluding SDG 17: Partnership for the Goals) that have been defined on the basis of expert-informed Scopus keyword searches augmented with machine learning [5].

This delivered two unique publication sets for each of the 16 SDGs:

Corpus 1: Publications identified by each SDG keyword search

Corpus 2: Publications ALSO identified by the sex and gender keyword search

Publications in Corpus 1 and 2 were tagged as such after matching using Scopus unique publication identifiers. This tagging was used as the basis for calculating the proportion of each SDG’s publications that include those related to sex and/or gender research topics as well as for developing topical maps using VOSviewer.

*Create sex and gender overlay maps:* To generate the sex and gender overlay of the map, which is not typically available in a Scopus-generated VOSviewer map of a publication set, we set up a small work-around to ensure that VOSviewer could read the original underlying data files in such a way to deliver the overlay we were presenting. In this case, we re-used the Cited by column generated by the Scopus extract for the purpose of sex and gender consideration.

**Methodology: mapping**

*Mapping tool:* The topical maps presented in this study were developed using VOSviewer. VOSviewer is “a software tool used for constructing and visualizing bibliometric networks” [2]; the current version at the time of analysis (Version 1.6.18) was employed. This tool uses natural language processing and network mapping techniques to process publication data exported from Scopus for visualization and further analysis.

*Timeframe:* For the maps generated for this study, we limited the results to 2020 only.

***Mapping settings***

*Sampling:* Owing to the processing limits of VOSviewer, where SDG publication sets were too large for mapping they have been randomly down-sampled to approximately 20,000 publications; this applies to all SDG publication sets except for SDG 1, which resulted in fewer than 20,000 publications and so all publications were included because that volume falls within the processing power of VOSviewer.

*Counting:* In VOSviewer, we applied binary counting of terms, meaning that the presence or absence of a term in a publication was used for determining the occurrence frequencies and term co-occurrence, not the number of occurrences of a term in a publication.

*Term occurrence threshold:* We applied a term occurrence threshold of at least 100 occurrences for inclusion in the map across each SDG’s publication set with the exception of SDG 1, where the smaller volume of retrieved publications meant that a threshold of at least 50 publications was more appropriate; these thresholds were selected heuristically as a trade-off between comprehensiveness and readability/interpretability of the resulting maps, and other thresholds did not materially alter our observations

*Relevance:* VOSviewer’s default setting to map 60% of the most relevant terms (based on the calculated relevance score) was selected since “terms with a high relevance score tend to represent specific topics covered by the text data, while terms with a low relevance score tend to be of a general nature and tend not to be representative of any specific topic. By excluding terms with a low relevance score, general terms are filtered out and the focus shifts to more specific and more informative terms. By default, 40% of the terms are excluded based on their relevance score” [6].

*Validation:* To further validate the specificity of the sex and gender keyword search, we carefully examined the results of the approach described above for SDG 5: Gender Equality. We found the expected high degree of overlap between the SDG 5 publication set and those publications within it tagged as also being identified by the sex and gender keyword search.

***Viewing the maps****Map files:* The files required for all maps presented in this study are available [7]. To open and inspect the maps, the user will need access to VOSviewer (available at https://www.vosviewer.com). A pair of .csv files is provided for each SDG 1 - 16: . From within the VOSviewer tool, the pair of .csv files can be used to Open the map file.

*Overlay visualization:* The sex and gender overlay maps can be viewed by selecting the 'Overlay Visualization' tab within the mapping tool and then selecting "Avg. citations": this actually reflects the extent to which the terms consider sex and gender.

**References**

1. International Center for the Study of Research [Internet]. ICSR Lab, c2020 [cited 2021 Dec 21]. Available from: <https://www.elsevier.com/icsr/icsrlab>
2. CWTS [Internet]. Welcome to VOSviewer; c2021 [cited 2021 Dec 21]. Available from: <https://www.vosviewer.com>
3. Elsevier [Internet]. Gender in the Global Research Landscape Mendeley Group; c2017 [cited 2021 Dec 21]. Available from: <https://www.mendeley.com/community/gender-in-the-global-research-landscape/>
4. UNICEF. Gender Equality, Glossary of Terms and Concepts. 2017 Nov [cited 2021 Dec 21]. Available from: <https://www.unicef.org/rosa/media/1761/file/Gender%20glossary%20of%20terms%20and%20concepts%20.pdf>
5. Rivest M, Kashnitsky Y, Bédard-Vallée A, Campbell D, Khayat P, Labrosse I, et al. Improving the Scopus and Aurora queries to identify research that supports the United Nations' Sustainable Development Goals (SDGs) 2021. Mendeley Data. 2021 Aug 26 [cited 2021 Dec 21]. Available from: <https://elsevier.digitalcommonsdata.com/datasets/9sxdykm8s4/4> doi:10.17632/9sxdykm8s4.4
6. CWTS [Internet]. VOSviewer Manual for version 1.6.17; c2021 [cited 2022 Aug 31]. Available from: <https://www.vosviewer.com/getting-started#vosviewer-manual>
7. Herbert R, James K, Plume A, Falk-Krzesinksi H. Data for: Sustainability through a gender lens: The extent to which research on UN Sustainable Development Goals includes sex and gender consideration. Mendeley Data. 2022 Aug 31 [cited 2022 Aug 31]. Available from <https://elsevier.digitalcommonsdata.com/datasets/8kthxbmmm5/1>. DOI: 10.17632/8kthxbmmm5.2

**S1 Table 2: Counts and proportion of SDG publications that include sex and gender keywords, 2015-2020.**

| **SDG No.** | **Sustainable Development Goal** | **2015 publications identified by SDG query** | **2015 publications ALSO identified by sex and gender keyword search** | **2015 proportion of SDG publications that include sex and gender keywords** | **2016 publications identified by SDG query** | **2016 publications ALSO identified by sex and gender keyword search** | **2016 proportion of SDG publications that include sex and gender keywords** | **2017 publications identified by SDG query** | **2017 publications ALSO identified by sex and gender keyword search** | **2017 proportion of SDG publications that include sex and gender keywords** | **2018 publications identified by SDG query** | **2018 publications ALSO identified by sex and gender keyword search** | **2018 proportion of SDG publications that include sex and gender keywords** | **2019 publications identified by SDG query** | **2019 publications ALSO identified by sex and gender keyword search** | **2019 proportion of SDG publications that include sex and gender keywords** | **2020 publications identified by SDG query** | **2020 publications ALSO identified by sex and gender keyword search** | **2020 proportion of SDG publications that include sex and gender keywords** |
| --- | --- | --- | --- | --- | --- | --- | --- | --- | --- | --- | --- | --- | --- | --- | --- | --- | --- | --- | --- |
| 1 | No Poverty | 8,058 | 2,513 | 31% | 8,727 | 2,666 | 31% | 9,437 | 2,922 | 31% | 10,015 | 3,207 | 32% | 11,326 | 3,725 | 33% | 13,424 | 4,142 | 31% |
| 2 | Zero Hunger | 22,150 | 4,367 | 20% | 23,741 | 4,768 | 20% | 25,252 | 5,104 | 20% | 27,888 | 5,586 | 20% | 30,947 | 6,203 | 20% | 37,067 | 7,335 | 20% |
| 3 | Good Health and Well-being | 307,041 | 195,044 | 64% | 313,007 | 197,981 | 63% | 315,343 | 202,407 | 64% | 329,287 | 211,349 | 64% | 351,024 | 224,181 | 64% | 417,443 | 256,741 | 62% |
| 4 | Quality Education | 21,583 | 5,268 | 24% | 22,758 | 5,457 | 24% | 24,228 | 6,262 | 26% | 27,208 | 7,167 | 26% | 30,776 | 8,020 | 26% | 37,206 | 9,302 | 25% |
| 5 | Gender Equality | 15,521 | 14,622 | 94% | 16,254 | 15,344 | 94% | 16,935 | 16,082 | 95% | 18,809 | 17,855 | 95% | 21,240 | 20,261 | 95% | 25,601 | 24,319 | 95% |
| 6 | Clean Water and Sanitation | 31,241 | 1,607 | 5% | 35,109 | 1,825 | 5% | 35,789 | 1,838 | 5% | 39,656 | 2,072 | 5% | 44,141 | 2,129 | 5% | 51,057 | 2,349 | 5% |
| 7 | Affordable and Clean Energy | 70,699 | 669 | 1% | 75,632 | 699 | 1% | 82,313 | 771 | 1% | 90,972 | 812 | 1% | 102,576 | 882 | 1% | 112,053 | 1,037 | 1% |
| 8 | Decent Work and Economic Growth | 21,292 | 3,110 | 15% | 23,107 | 3,183 | 14% | 25,295 | 3,542 | 14% | 28,761 | 4,059 | 14% | 33,054 | 4,570 | 14% | 40,920 | 5,639 | 14% |
| 9 | Industry, Innovation and Infrastructure | 28,980 | 848 | 3% | 32,219 | 894 | 3% | 36,339 | 1,094 | 3% | 42,124 | 1,201 | 3% | 49,389 | 1,477 | 3% | 58,662 | 1,764 | 3% |
| 10 | Reduced Inequalities | 21,870 | 7,470 | 34% | 23,387 | 7,994 | 34% | 25,242 | 9,037 | 36% | 27,957 | 10,384 | 37% | 31,492 | 11,893 | 38% | 38,250 | 14,129 | 37% |
| 11 | Sustainable Cities and Communities | 31,463 | 2,319 | 7% | 34,564 | 2,479 | 7% | 36,956 | 2,652 | 7% | 43,107 | 3,026 | 7% | 48,771 | 3,494 | 7% | 57,878 | 3,992 | 7% |
| 12 | Responsible Consumption and Production | 19,308 | 680 | 4% | 21,598 | 712 | 3% | 23,789 | 836 | 4% | 26,917 | 1,027 | 4% | 30,621 | 1,173 | 4% | 37,391 | 1,533 | 4% |
| 13 | Climate Action | 24,681 | 661 | 3% | 26,912 | 747 | 3% | 28,915 | 813 | 3% | 31,864 | 891 | 3% | 35,217 | 1,065 | 3% | 42,699 | 1,336 | 3% |
| 14 | Life Below Water | 18,207 | 1,343 | 7% | 20,010 | 1,512 | 8% | 20,707 | 1,603 | 8% | 22,411 | 1,594 | 7% | 24,250 | 1,692 | 7% | 28,146 | 1,882 | 7% |
| 15 | Life on Land | 24,199 | 2,329 | 10% | 25,798 | 2,449 | 9% | 26,985 | 2,483 | 9% | 29,089 | 2,677 | 9% | 31,129 | 2,670 | 9% | 35,543 | 3,114 | 9% |
| 16 | Peace, Justice and Strong Institutions | 21,140 | 7,896 | 37% | 22,227 | 8,261 | 37% | 23,520 | 8,999 | 38% | 26,138 | 10,222 | 39% | 29,354 | 11,539 | 39% | 35,037 | 13,599 | 39% |
|  | Deduplicated total | 1,395,985 | 285,157 | 20% | 1,403,699 | 283,082 | 20% | 1,409,423 | 287,441 | 20% | 1,465,097 | 301,096 | 21% | 1,538,962 | 314,107 | 20% | 1,669,868 | 352,228 | 21% |

What follows is supplementary material which extends the approach described in our main paper to all 16 SDGs. For each SDG, two term maps are presented as individual figures, depicting the topical structure of the research connected to the SDG (the first in each pair, network map) and the extent to which those topics are associated with sex and/or gender (the second in each pair, overlay map).


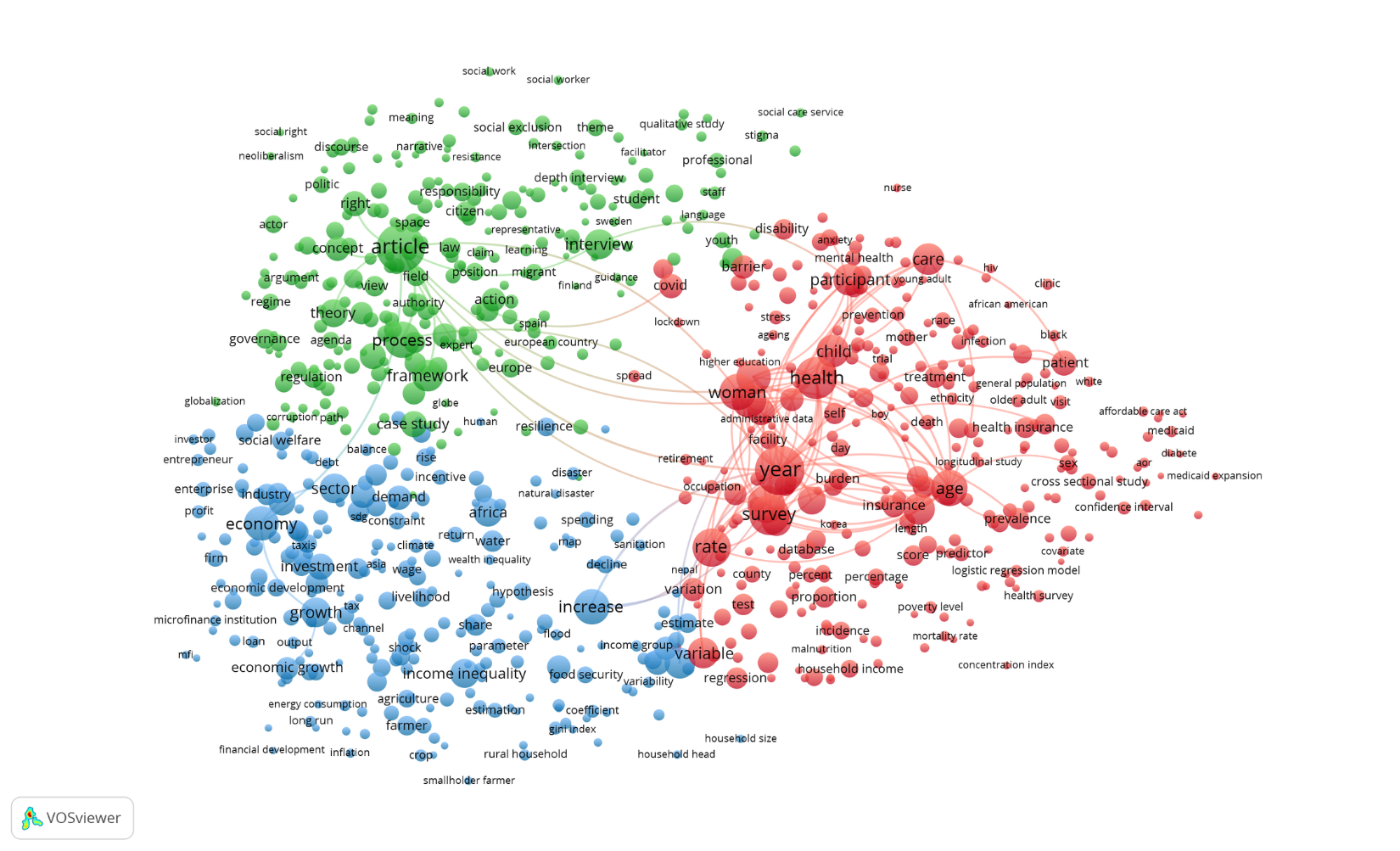


**S1 Fig. SDG 1: No Poverty term network map.**Binary counting (present/absent, not count of occurrences) was applied to terms in titles and abstracts of 13,424 publications in 2020, and those with at least 50 occurrences were mapped using VOSviewer. Node size indicates count of occurrences, and node proximity reflects frequency of co-occurrence (nodes close together co-occur more frequently than nodes far apart). In this network visualization, the colors indicate topical clusters.


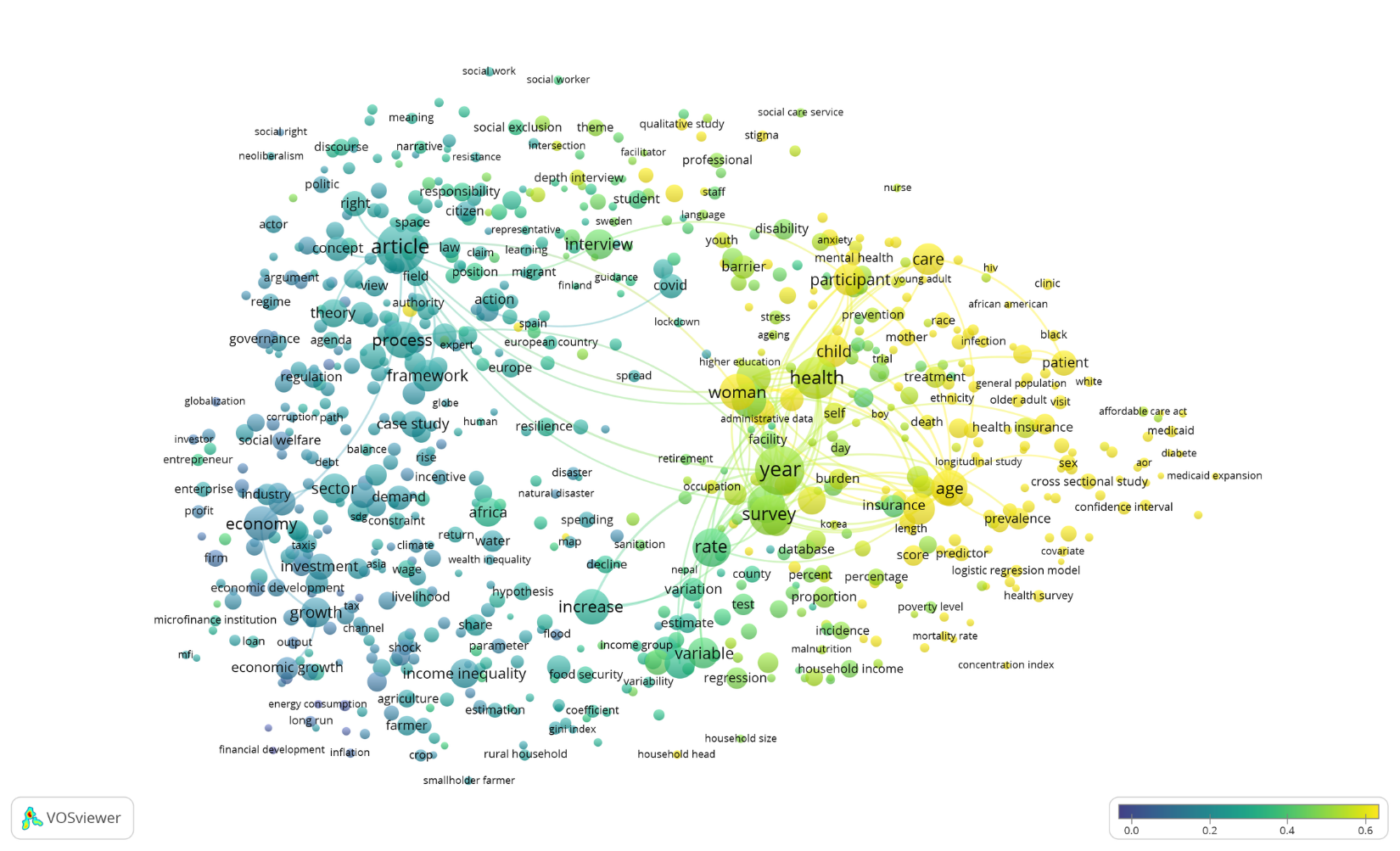


**S2 Fig. SDG 1: No Poverty term overlay map.**Binary counting (present/absent, not count of occurrences) was applied to terms in titles and abstracts of 13,424 publications in 2020, and those with at least 50 occurrences were mapped using VOSviewer. Node size indicates count of occurrences, and node proximity reflects frequency of co-occurrence (nodes close together co-occur more frequently than nodes far apart). In this overlay visualization, the color scale indicates the proportion of publications associated with the mapped terms that were also identified by the sex and gender keyword search: blue nodes indicate terms with relatively low consideration of sex and/or gender; yellow terms indicate terms with relatively high consideration of sex and/or gender.


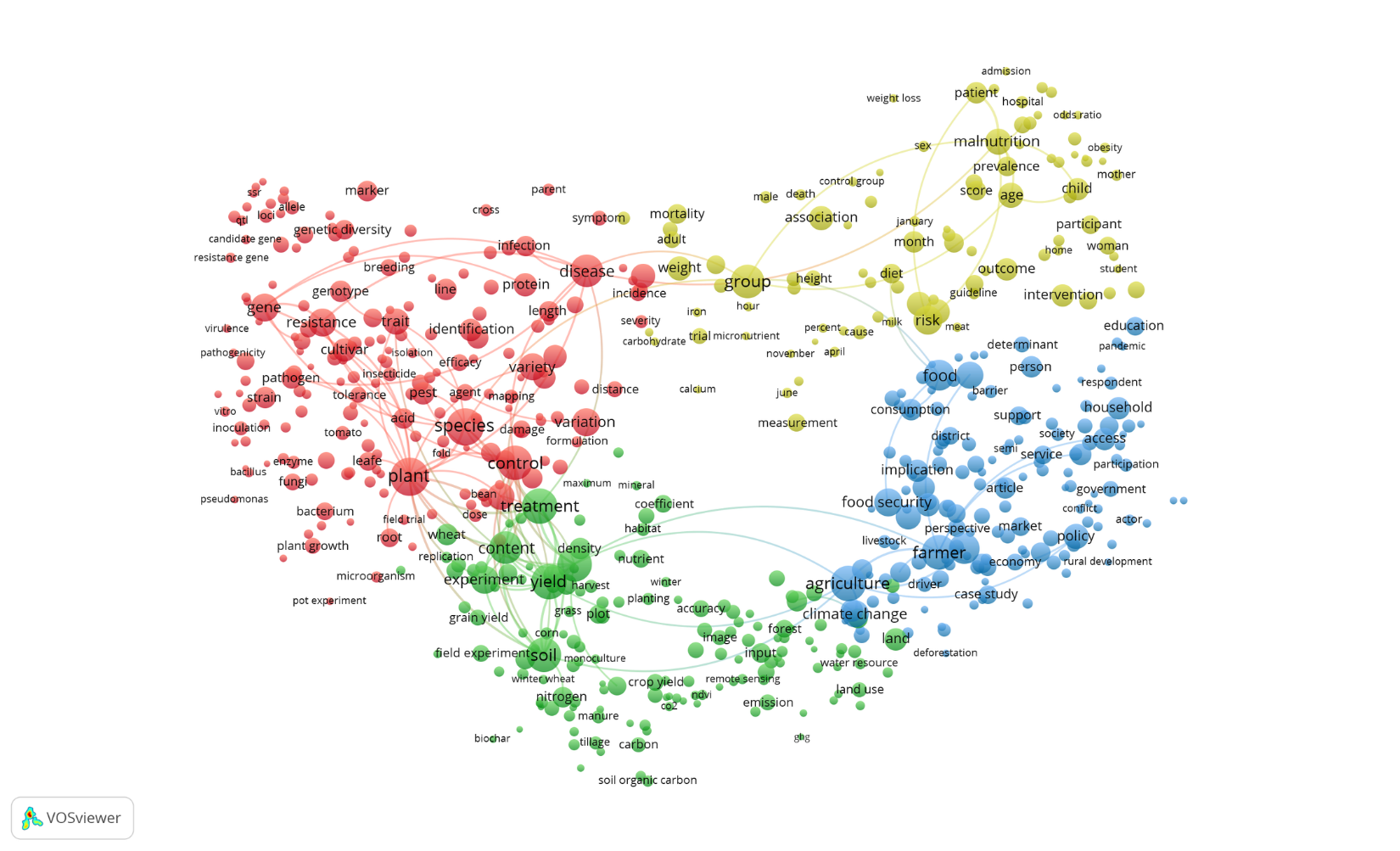


**S3 Fig. SDG 2: Zero Hunger term network map.**Binary counting (present/absent, not count of occurrences) was applied to terms in titles and abstracts of 19,979 publications in 2020 (sampled from 37,067 in total), and those with at least 100 occurrences were mapped using VOSviewer. Node size indicates count of occurrences, and node proximity reflects frequency of co-occurrence (nodes close together co-occur more frequently than nodes far apart). In this network visualization, the colors indicate topical clusters.


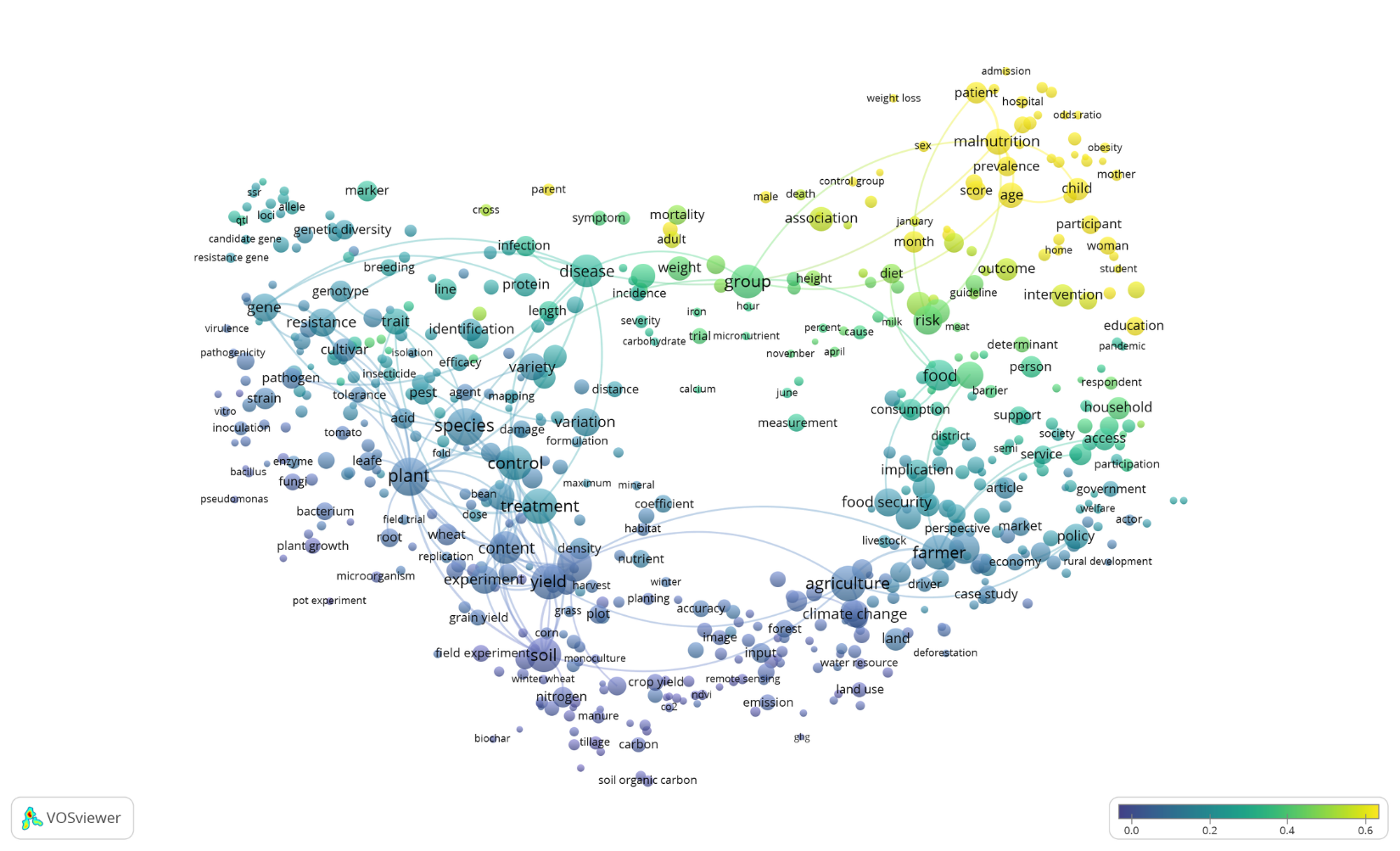


**S4 Fig. SDG 2: Zero Hunger term overlay map.**Binary counting (present/absent, not count of occurrences) was applied to terms in titles and abstracts of 19,979 publications in 2020 (sampled from 37,067 in total), and those with at least 100 occurrences were mapped using VOSviewer. Node size indicates count of occurrences, and node proximity reflects frequency of co-occurrence (nodes close together co-occur more frequently than nodes far apart). In this overlay visualization, the color scale indicates the proportion of publications associated with the mapped terms that were also identified by the sex and gender keyword search: blue nodes indicate terms with relatively low consideration of sex and/or gender; yellow terms indicate terms with relatively high consideration of sex and/or gender.


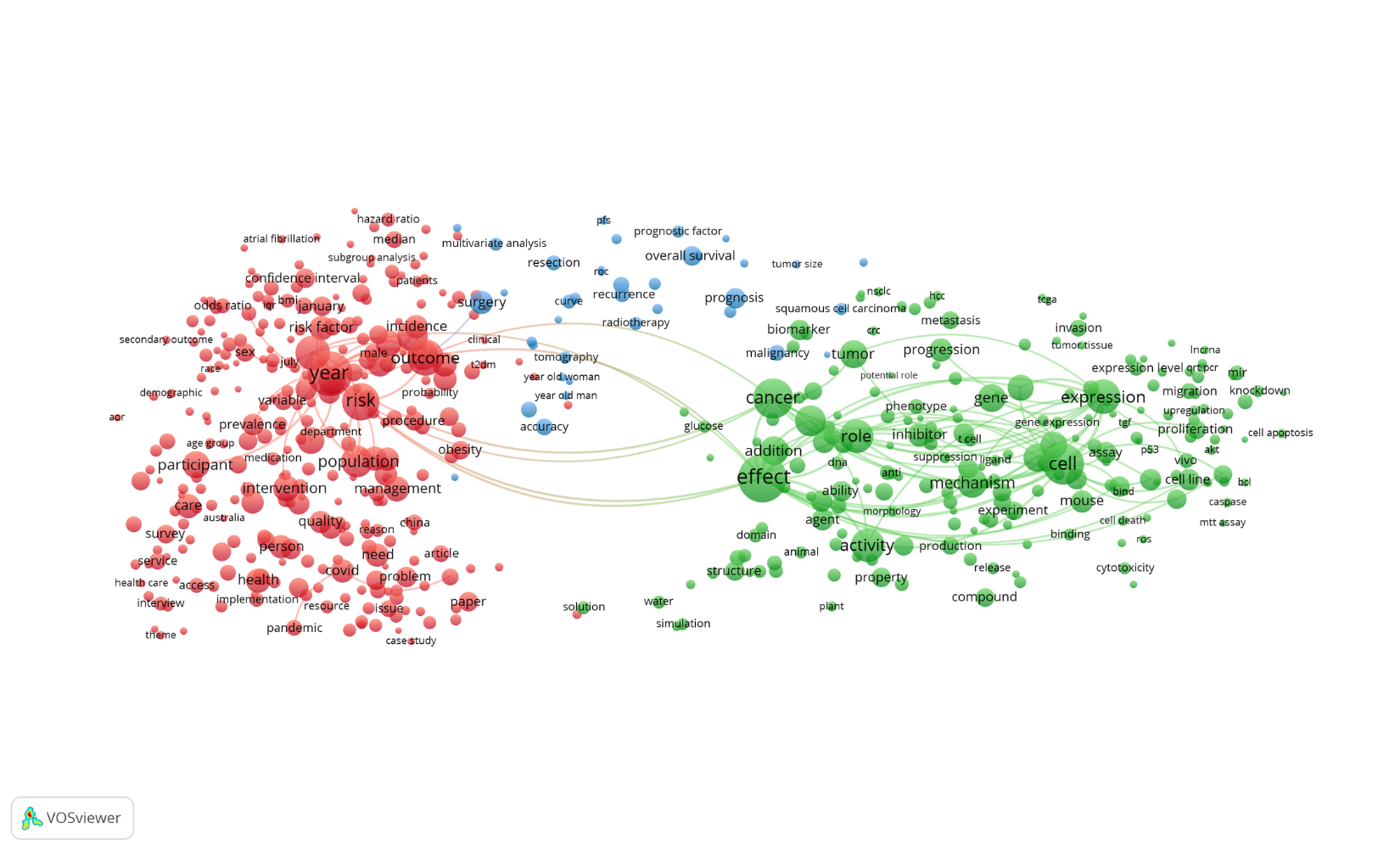


**S5 Fig. SDG 3: Good Health and Well-being term network map.**Binary counting (present/absent, not count of occurrences) was applied to terms in titles and abstracts of 19,983 publications in 2020 (sampled from 417,443 in total), and those with at least 100 occurrences were mapped using VOSviewer. Node size indicates count of occurrences, and node proximity reflects frequency of co-occurrence (nodes close together co-occur more frequently than nodes far apart). In this network visualization, colors indicate topical clusters.


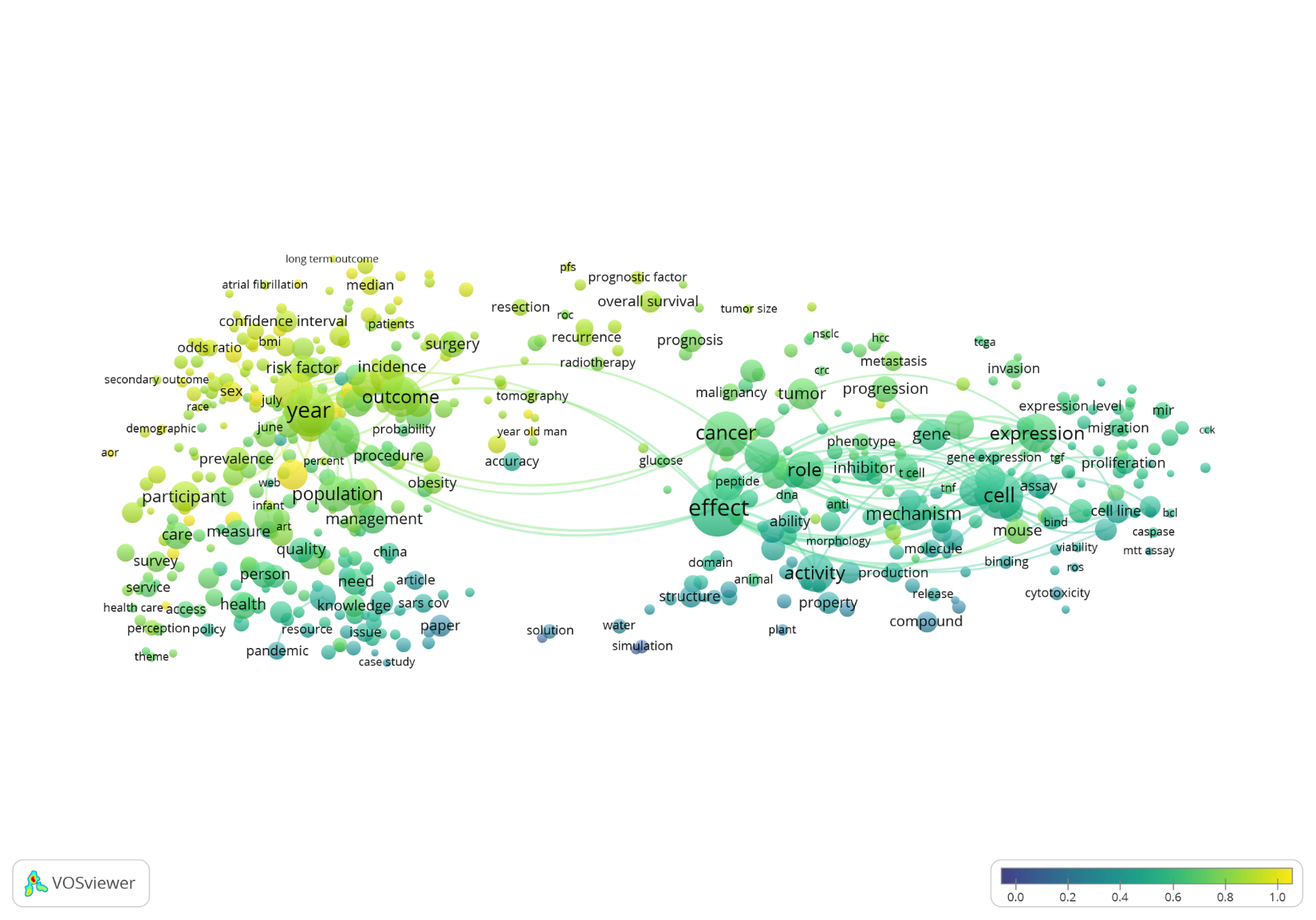


**S6 Fig. SDG 3: Good Health and Well-being term overlay map.**Binary counting (present/absent, not count of occurrences) was applied to terms in titles and abstracts of 19,983 publications in 2020 (sampled from 417,443 in total), and those with at least 100 occurrences were mapped using VOSviewer. Node size indicates count of occurrences, and node proximity reflects frequency of co-occurrence (nodes close together co-occur more frequently than nodes far apart). In this overlay visualization, the color scale indicates the proportion of publications associated with the mapped terms that were also identified by the sex and gender keyword search: blue nodes indicate terms with relatively low consideration of sex and/or gender; yellow terms indicate terms with relatively high consideration of sex and/or gender.


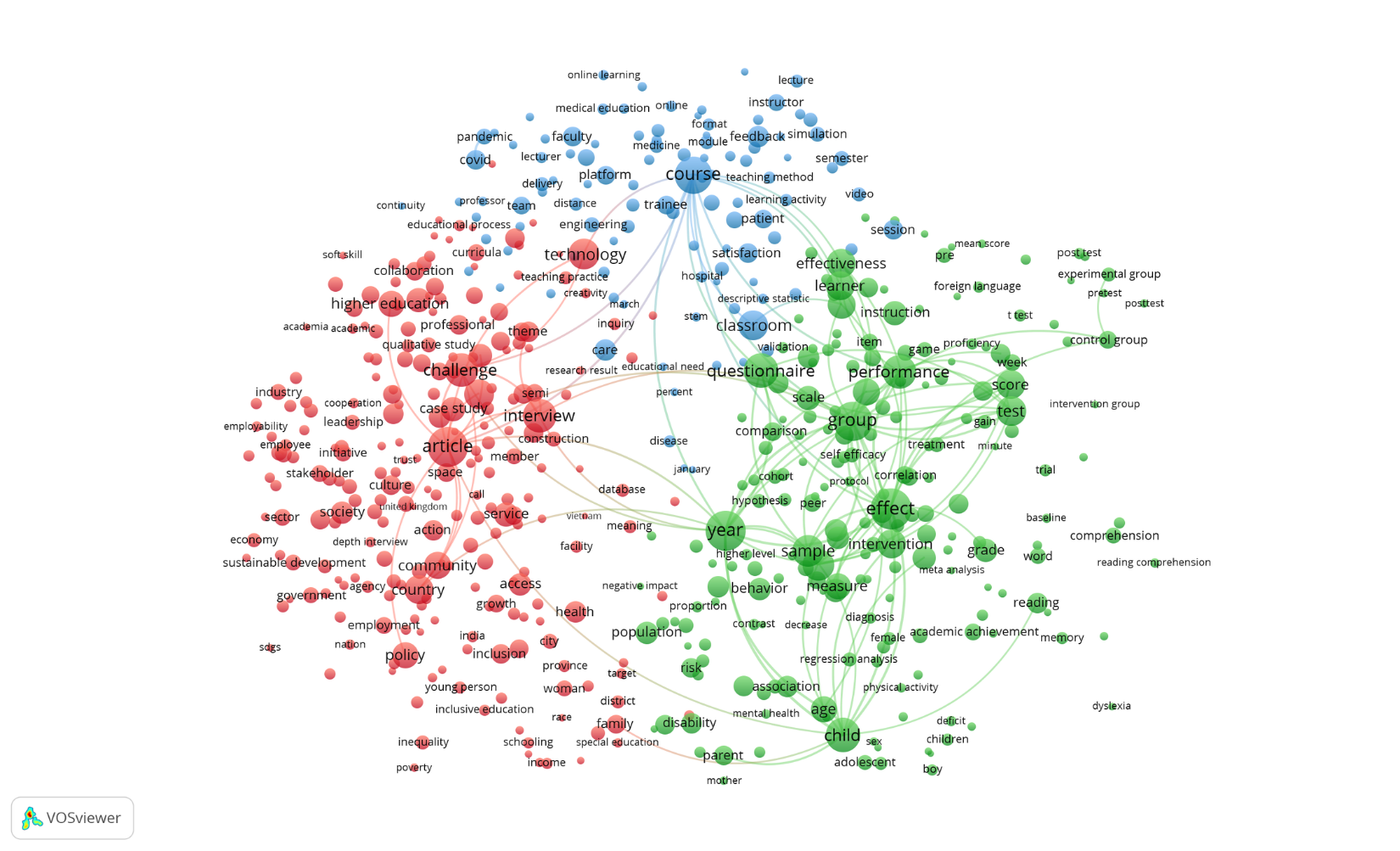


**S7 Fig. SDG 4: Quality Education term network map.**Binary counting (present/absent, not count of occurrences) was applied to terms in titles and abstracts of 20,030 publications in 2020 (sampled from 37,206 in total), and those with at least 100 occurrences were mapped using VOSviewer. Node size indicates count of occurrences, and node proximity reflects frequency of co-occurrence (nodes close together co-occur more frequently than nodes far apart). In this network visualization, the colors indicate topical clusters.


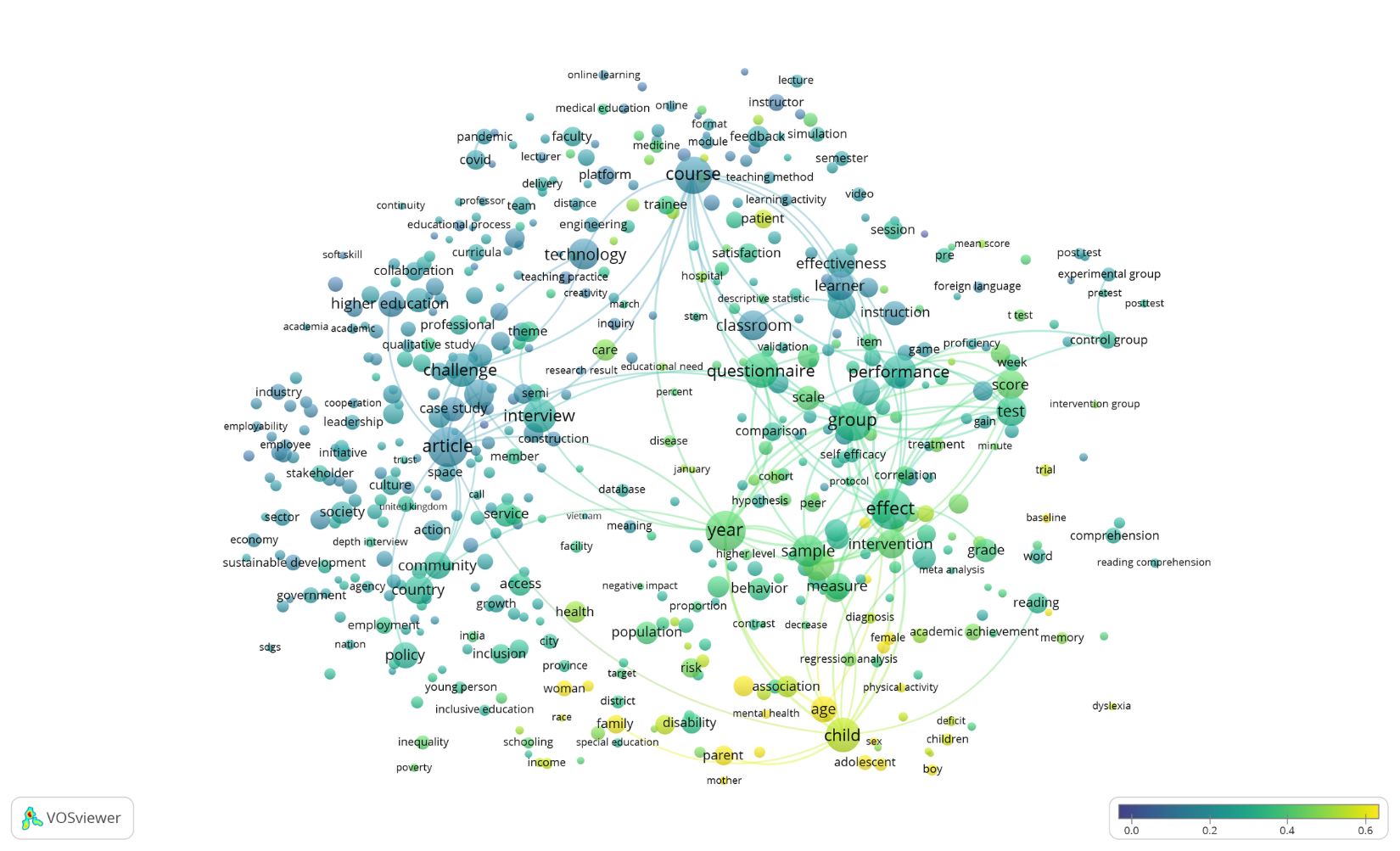


**S8 Fig. SDG 4: Quality Education term overlay map.**Binary counting (present/absent, not count of occurrences) was applied to terms in titles and abstracts of 20,030 publications in 2020 (sampled from 37,206 in total), and those with at least 100 occurrences were mapped using VOSviewer. Node size indicates count of occurrences, and node proximity reflects frequency of co-occurrence (nodes close together co-occur more frequently than nodes far apart). In this overlay visualization, the color scale indicates the proportion of publications associated with the mapped terms that were also identified by the sex and gender keyword search: blue nodes indicate terms with relatively low consideration of sex and/or gender; yellow terms indicate terms with relatively high consideration of sex and/or gender.

 
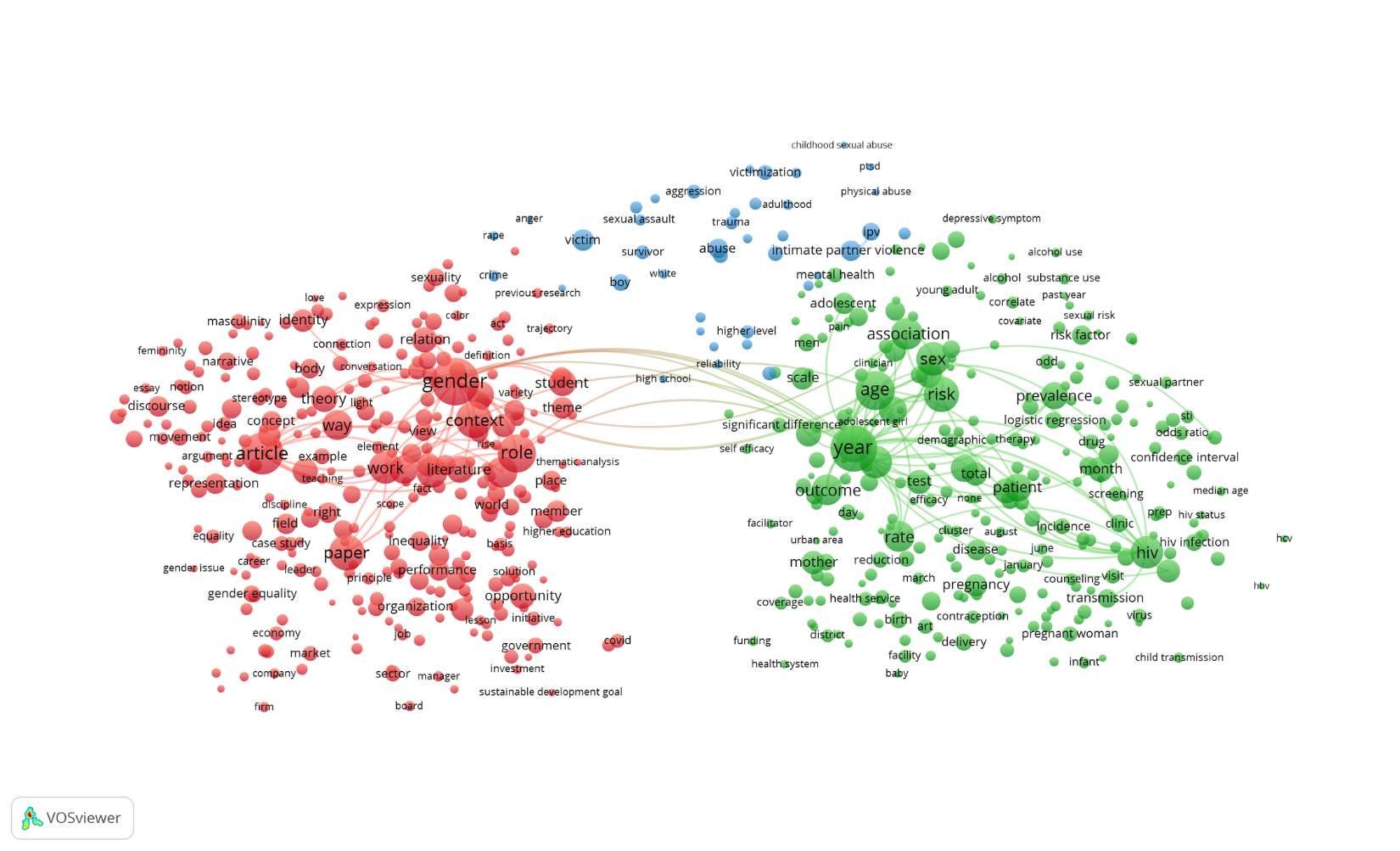


**S9 Fig. SDG 5: Gender Equality term network map.**Binary counting (present/absent, not count of occurrences) was applied to terms in titles and abstracts of 18,976 publications in 2020 (sampled from 25,601 in total), and those with at least 100 occurrences were mapped using VOSviewer. Node size indicates count of occurrences, and node proximity reflects frequency of co-occurrence (nodes close together co-occur more frequently than nodes far apart). In this network visualization, the  colors indicate topical clusters.


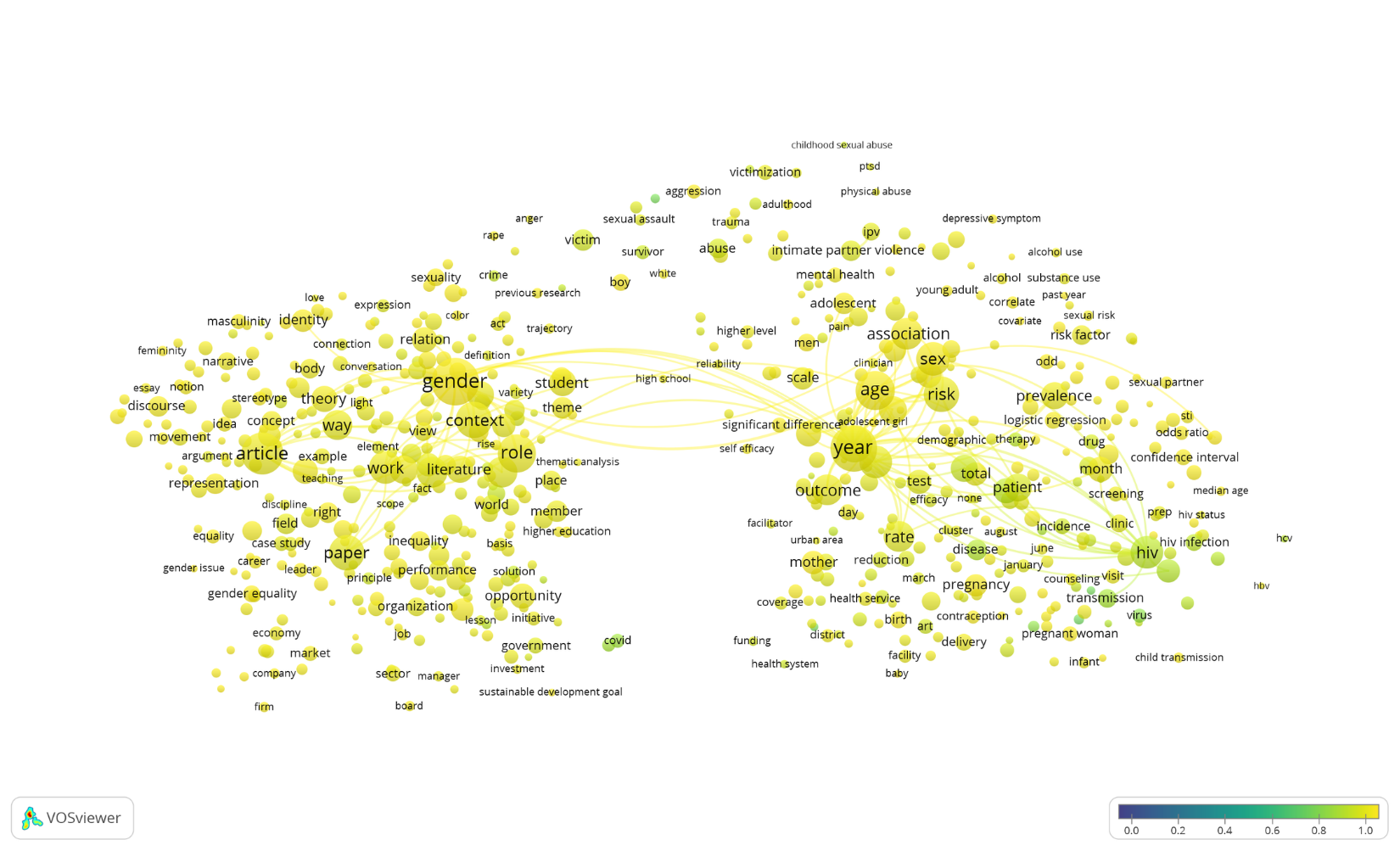


**S10 Fig. SDG 5: Gender Equality term overlay map.**Binary counting (present/absent, not count of occurrences) was applied to terms in titles and abstracts of 18,976 publications in 2020 (sampled from 25,601 in total), and those with at least 100 occurrences were mapped using VOSviewer. Node size indicates count of occurrences, and node proximity reflects frequency of co-occurrence (nodes close together co-occur more frequently than nodes far apart). In this overlay visualization, the color scale indicates the proportion of publications associated with the mapped terms that were also identified by the sex and gender keyword search: blue nodes indicate terms with relatively low consideration of sex and/or gender; yellow terms indicate terms with relatively high consideration of sex and/or gender.


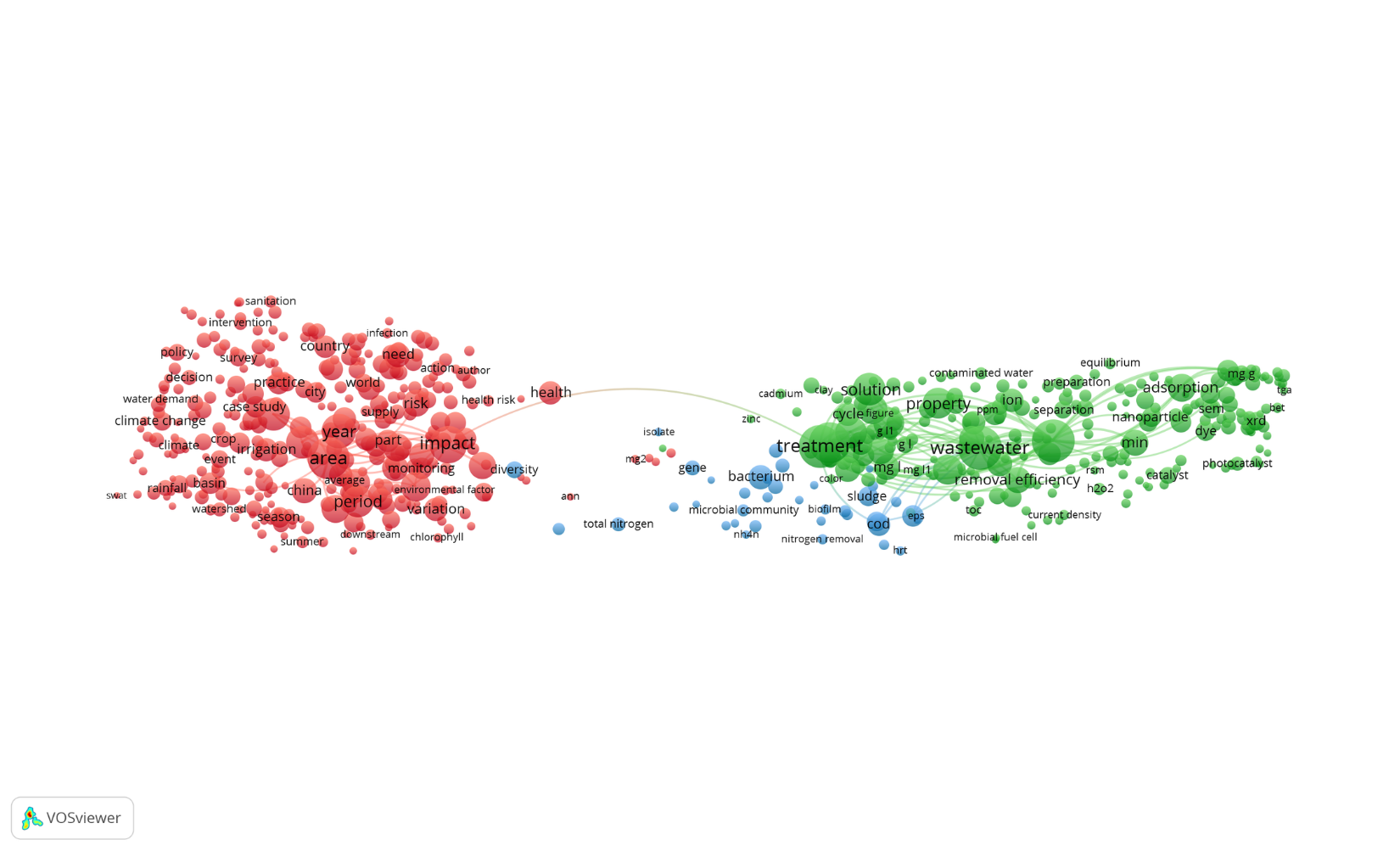


**S11 Fig. SDG 6: Clean Water and Sanitation term network map.**Binary counting (present/absent, not count of occurrences) was applied to terms in titles and abstracts of 20,047 publications in 2020 (sampled from 51,057 in total), and those with at least 100 occurrences were mapped using VOSviewer. Node size indicates count of occurrences, and node proximity reflects frequency of co-occurrence (nodes close together co-occur more frequently than nodes far apart). In this network visualization, the colors indicate topical clusters.


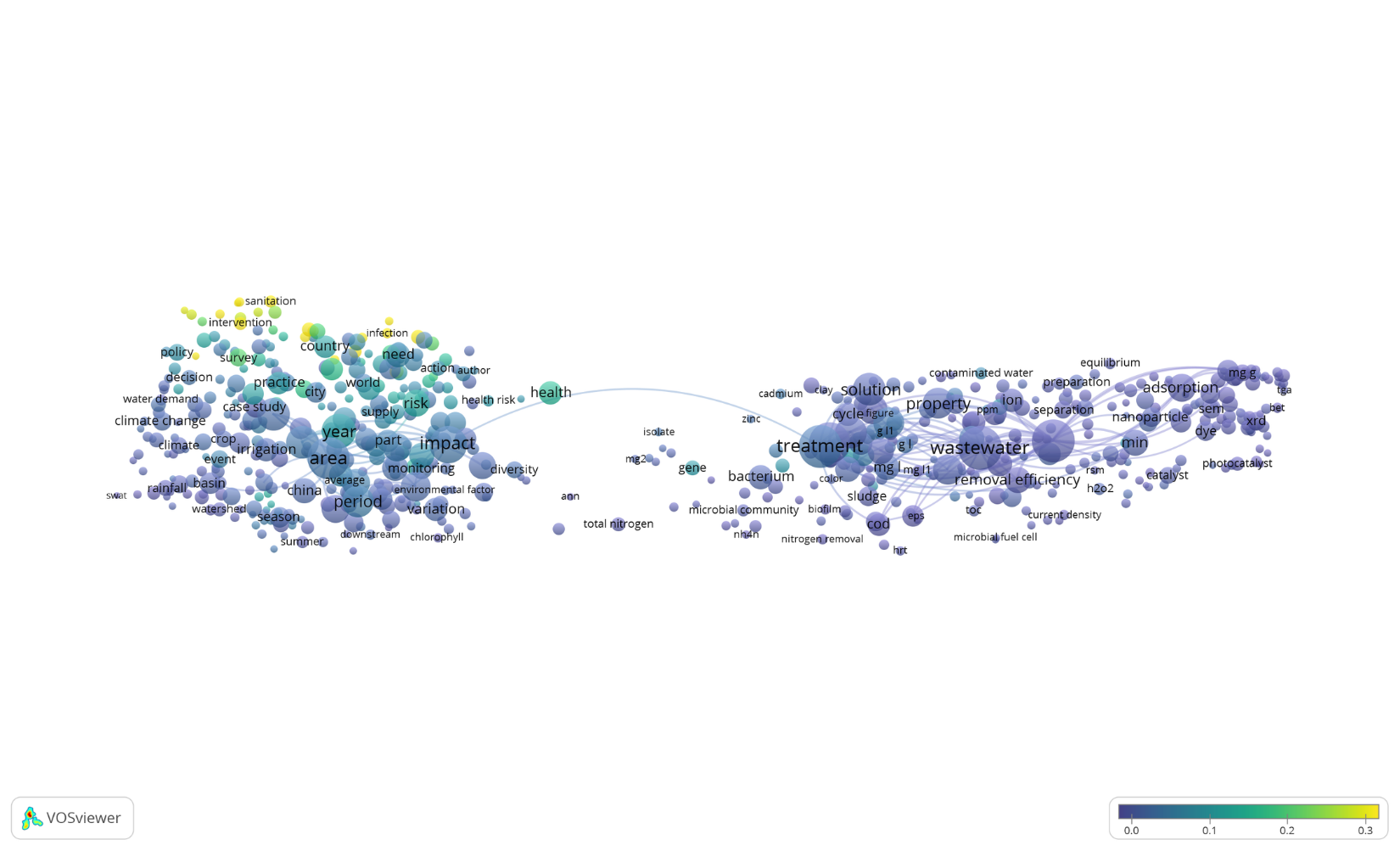


**S12 Fig. SDG 6: Clean Water and Sanitation term overlay map.**Binary counting (present/absent, not count of occurrences) was applied to terms in titles and abstracts of 20,047 publications in 2020 (sampled from 51,057 in total), and those with at least 100 occurrences were mapped using VOSviewer. Node size indicates count of occurrences, and node proximity reflects frequency of co-occurrence (nodes close together co-occur more frequently than nodes far apart). In this overlay visualization, the color scale indicates the proportion of publications associated with the mapped terms that were also identified by the sex and gender keyword search: blue nodes indicate terms with relatively low consideration of sex and/or gender; yellow terms indicate terms with relatively high consideration of sex and/or gender.


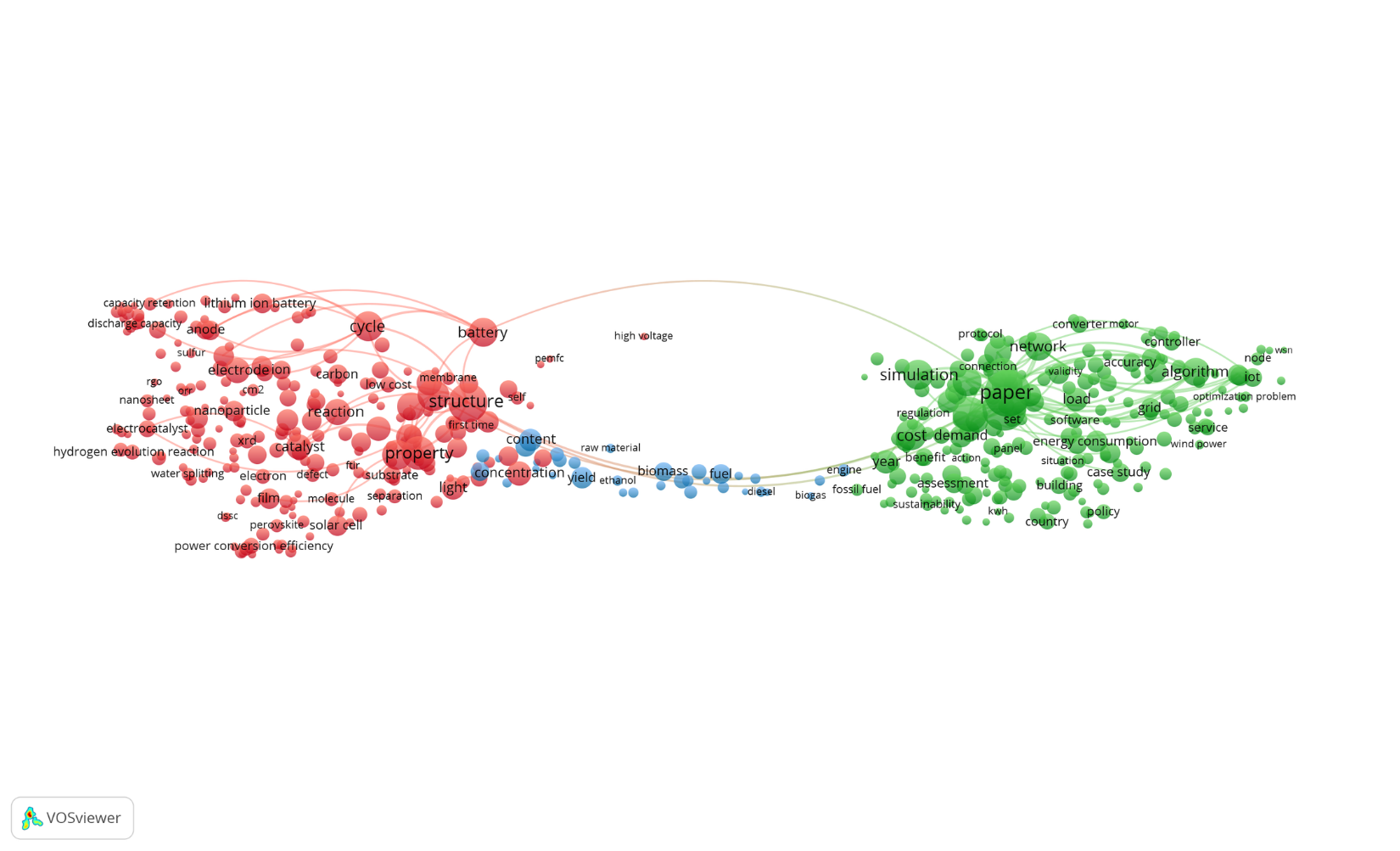


**S13 Fig. SDG 7: Affordable and Clean Energy term network map.**Binary counting (present/absent, not count of occurrences) was applied to terms in titles and abstracts of 20,142 publications in 2020 (sampled from 112,053 in total), and those with at least 100 occurrences were mapped using VOSviewer. Node size indicates count of occurrences, and node proximity reflects frequency of co-occurrence (nodes close together co-occur more frequently than nodes far apart). In this network visualization, the colors indicate topical clusters.


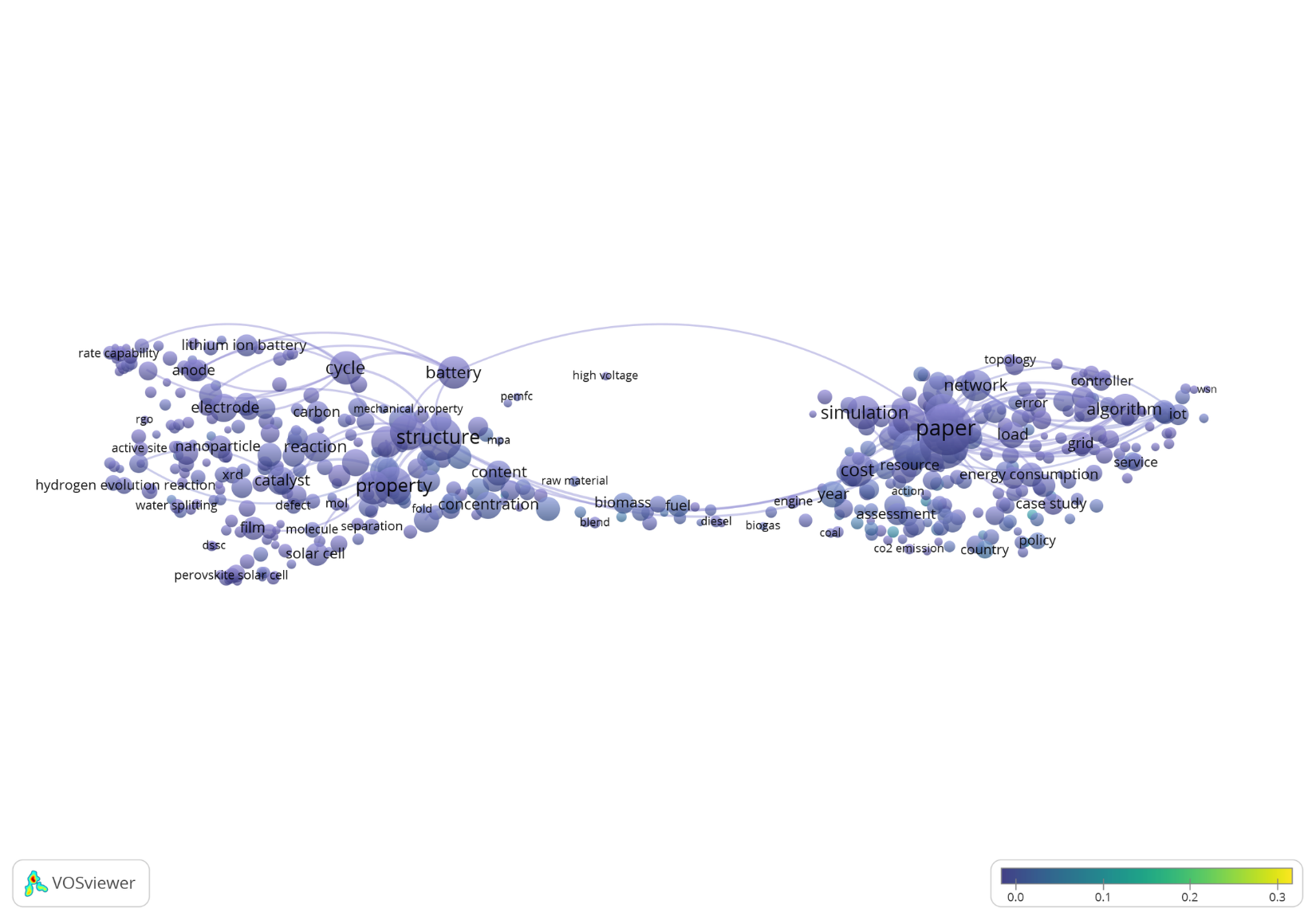


**S14 Fig. SDG 7: Affordable and Clean Energy term overlay map.**Binary counting (present/absent, not count of occurrences) was applied to terms in titles and abstracts of 20,142 publications in 2020 (sampled from 112,053 in total), and those with at least 100 occurrences were mapped using VOSviewer. Node size indicates count of occurrences, and node proximity reflects frequency of co-occurrence (nodes close together co-occur more frequently than nodes far apart). In this overlay visualization, the color scale indicates the proportion of publications associated with the mapped terms that were also identified by the sex and gender keyword search: blue nodes indicate terms with relatively low consideration of sex and/or gender; yellow terms indicate terms with relatively high consideration of sex and/or gender.


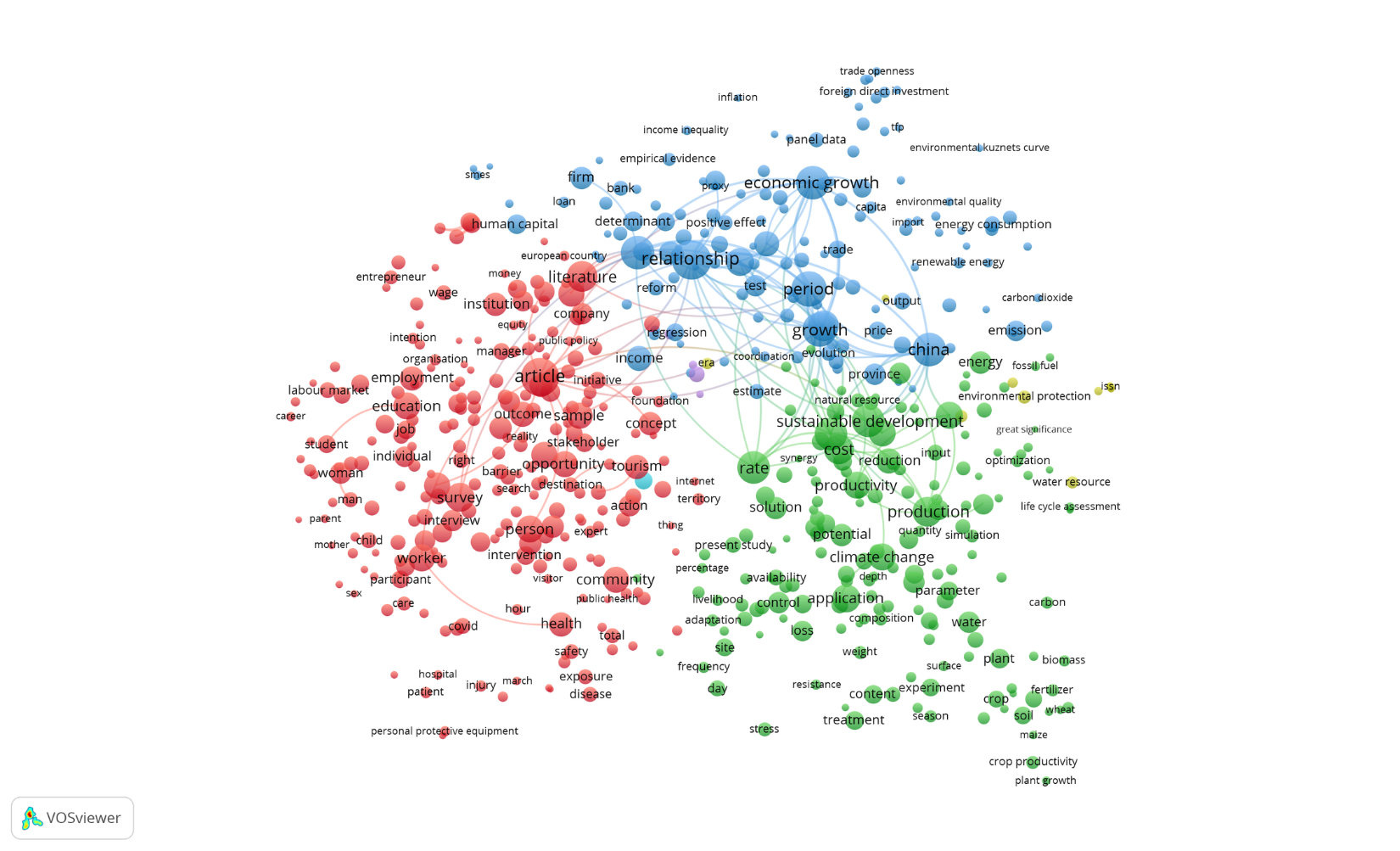


**S15 Fig. SDG 8: Decent Work and Economic Growth term network map.**Binary counting (present/absent, not count of occurrences) was applied to terms in titles and abstracts of 20,014 publications in 2020 (sampled from 40,920 in total), and those with at least 100 occurrences were mapped using VOSviewer. Node size indicates count of occurrences, and node proximity reflects frequency of co-occurrence (nodes close together co-occur more frequently than nodes far apart). In this network visualization, the colors indicate topical clusters.


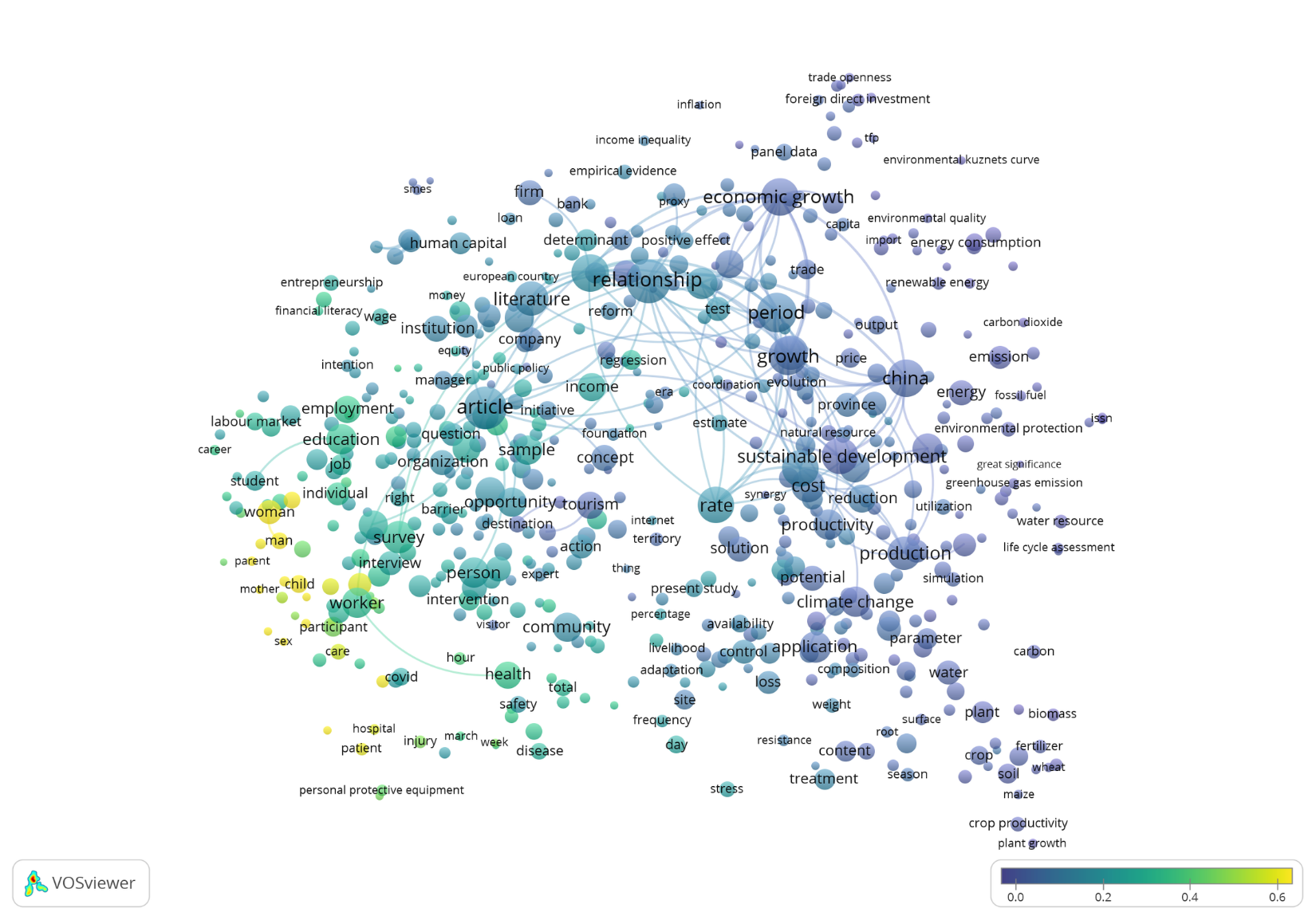


**S16 Fig. SDG 8: Decent Work and Economic Growth term overlay map.**Binary counting (present/absent, not count of occurrences) was applied to terms in titles and abstracts of 20,014 publications in 2020 (sampled from 40,920 in total), and those with at least 100 occurrences were mapped using VOSviewer. Node size indicates count of occurrences, and node proximity reflects frequency of co-occurrence (nodes close together co-occur more frequently than nodes far apart). In this overlay visualization, the color scale indicates the proportion of publications associated with the mapped terms that were also identified by the sex and gender keyword search: blue nodes indicate terms with relatively low consideration of sex and/or gender; yellow terms indicate terms with relatively high consideration of sex and/or gender.


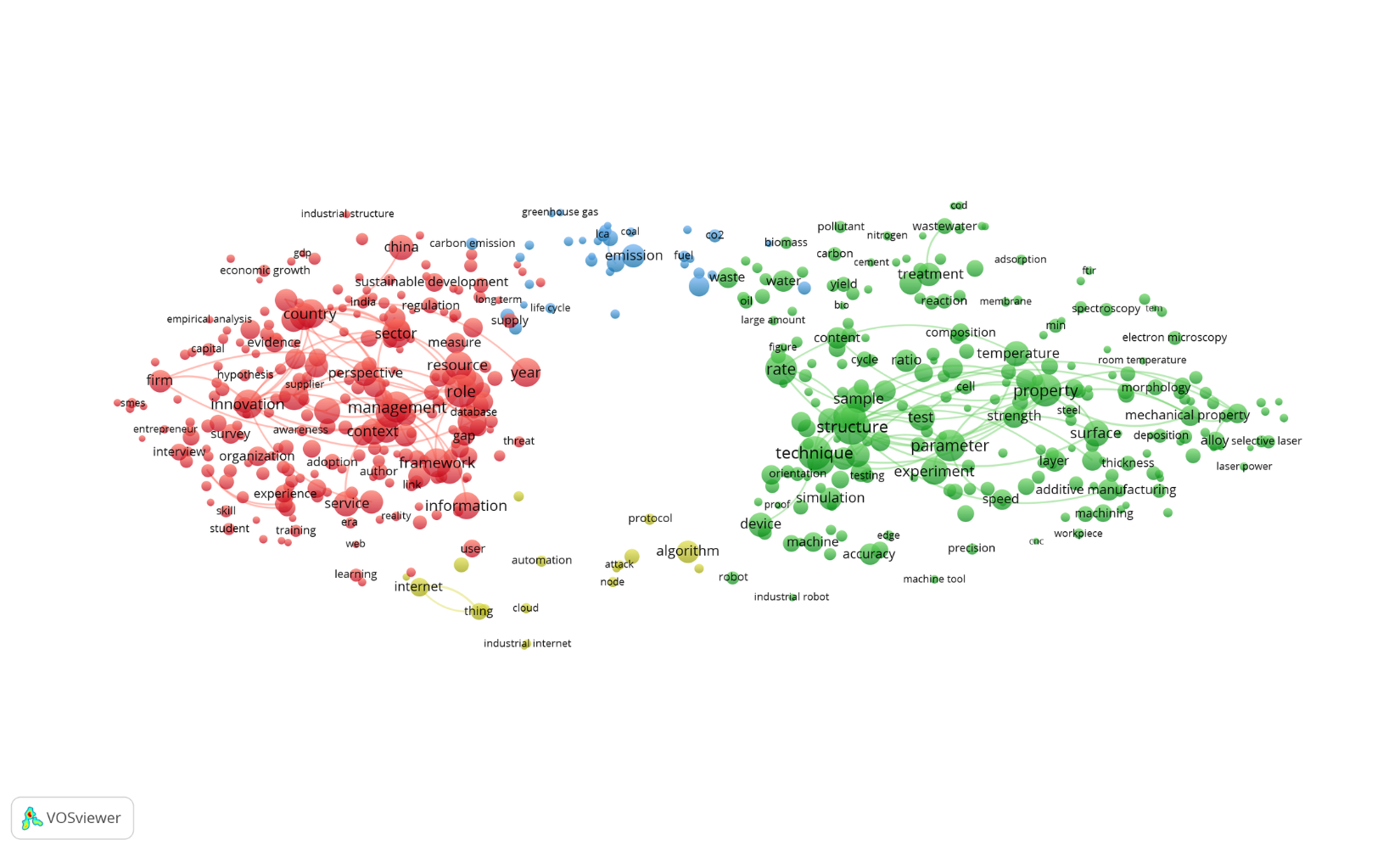


**S17 Fig. SDG 9: Industry, Innovation and Infrastructure term network map.**Binary counting (present/absent, not count of occurrences) was applied to terms in titles and abstracts of 20,135 publications in 2020 (sampled from 58,662 in total), and those with at least 100 occurrences were mapped using VOSviewer. Node size indicates count of occurrences, and node proximity reflects frequency of co-occurrence (nodes close together co-occur more frequently than nodes far apart). In this network visualization, the colors indicate topical clusters.


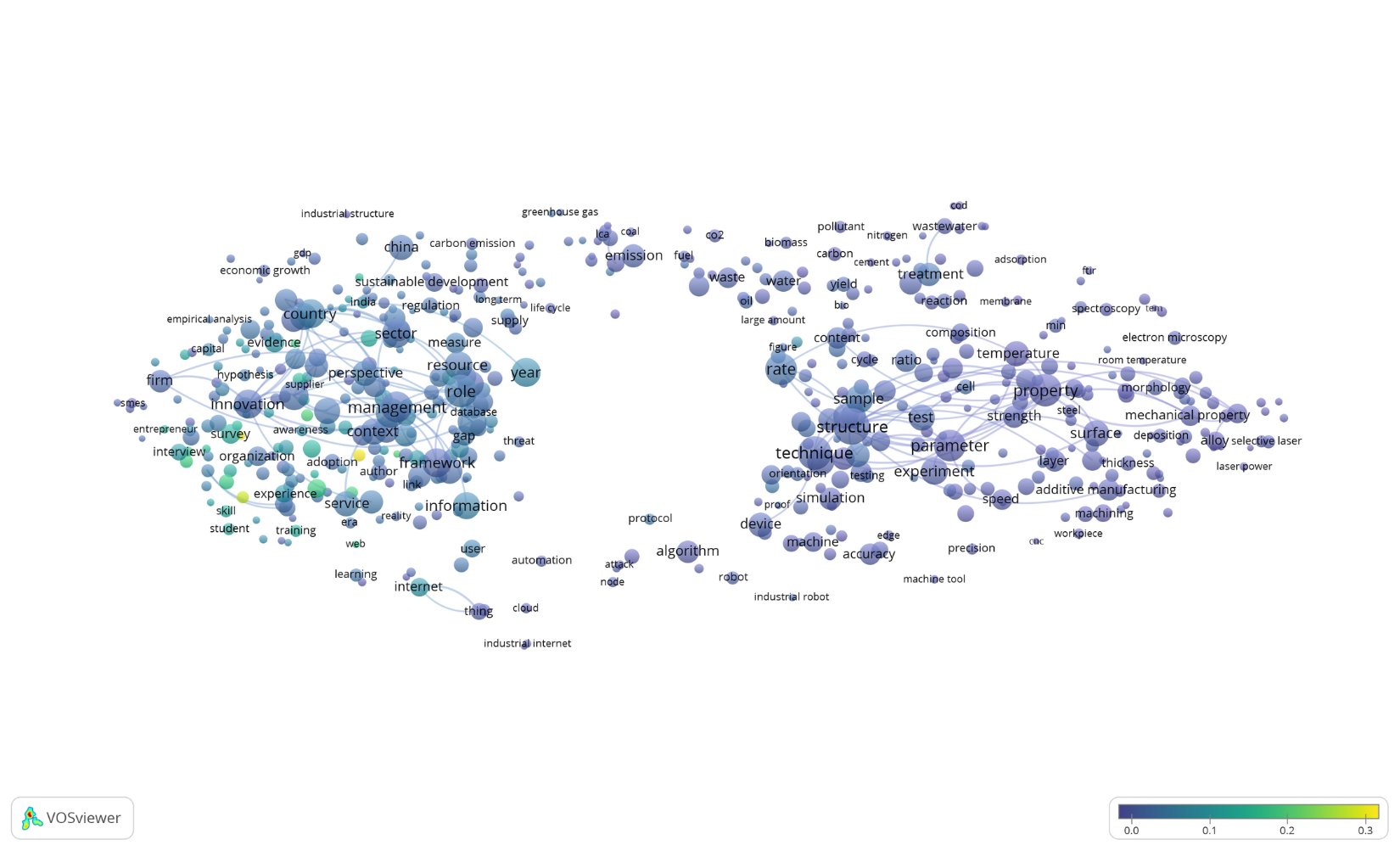


**S18 Fig. SDG 9: Industry, Innovation and Infrastructure term overlay map.**Binary counting (present/absent, not count of occurrences) was applied to terms in titles and abstracts of 20,135 publications in 2020 (sampled from 58,662 in total), and those with at least 100 occurrences were mapped using VOSviewer. Node size indicates count of occurrences, and node proximity reflects frequency of co-occurrence (nodes close together co-occur more frequently than nodes far apart). In this overlay visualization, the color scale indicates the proportion of publications associated with the mapped terms that were also identified by the sex and gender keyword search: blue nodes indicate terms with relatively low consideration of sex and/or gender; yellow terms indicate terms with relatively high consideration of sex and/or gender.


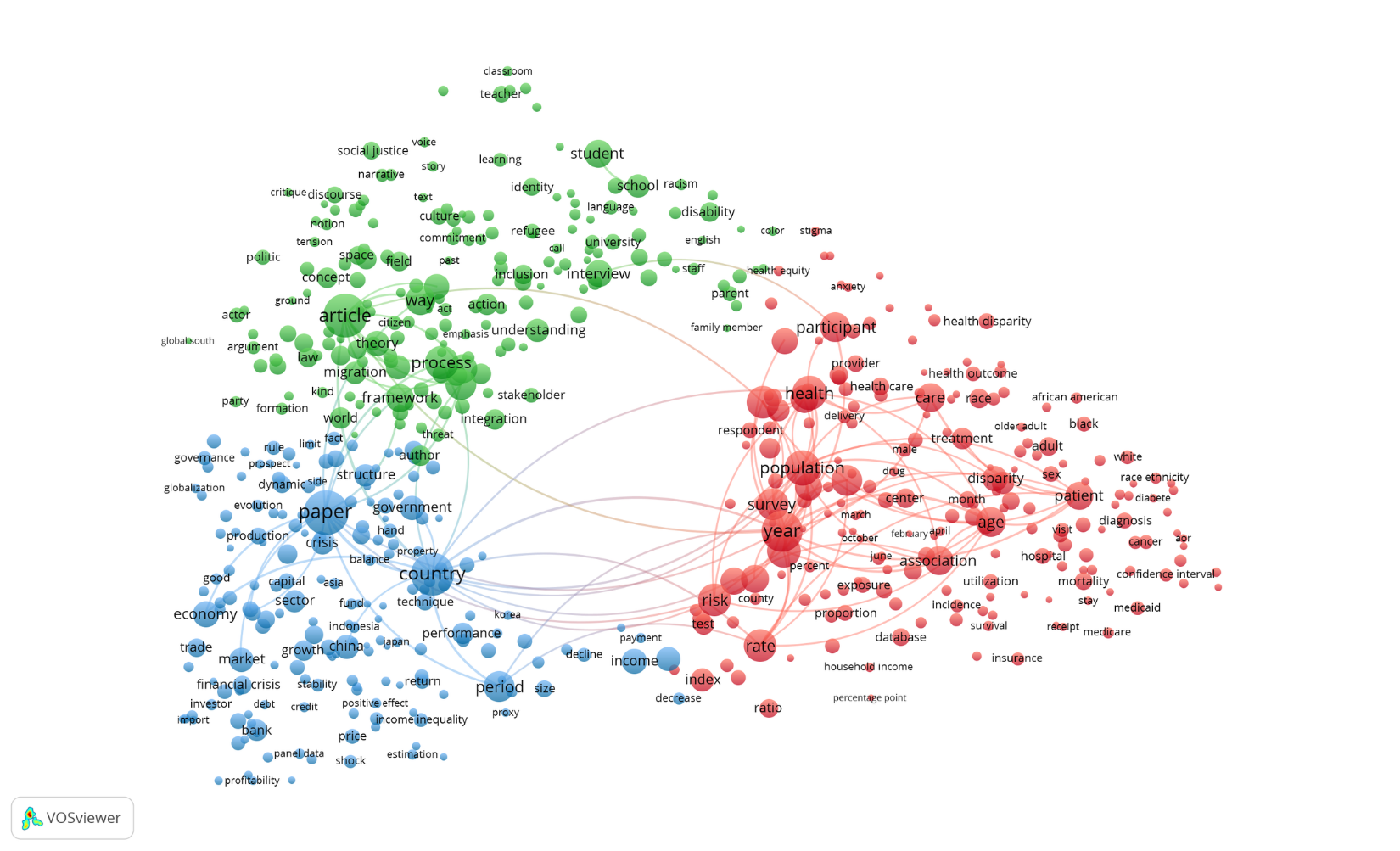


**S19 Fig. SDG 10: Reduced Inequalities term network map.**Binary counting (present/absent, not count of occurrences) was applied to terms in titles and abstracts of 20,025 publications in 2020 (sampled from 38,250 in total), and those with at least 100 occurrences were mapped using VOSviewer. Node size indicates count of occurrences, and node proximity reflects frequency of co-occurrence (nodes close together co-occur more frequently than nodes far apart). In this network visualization, the colors indicate topical clusters.


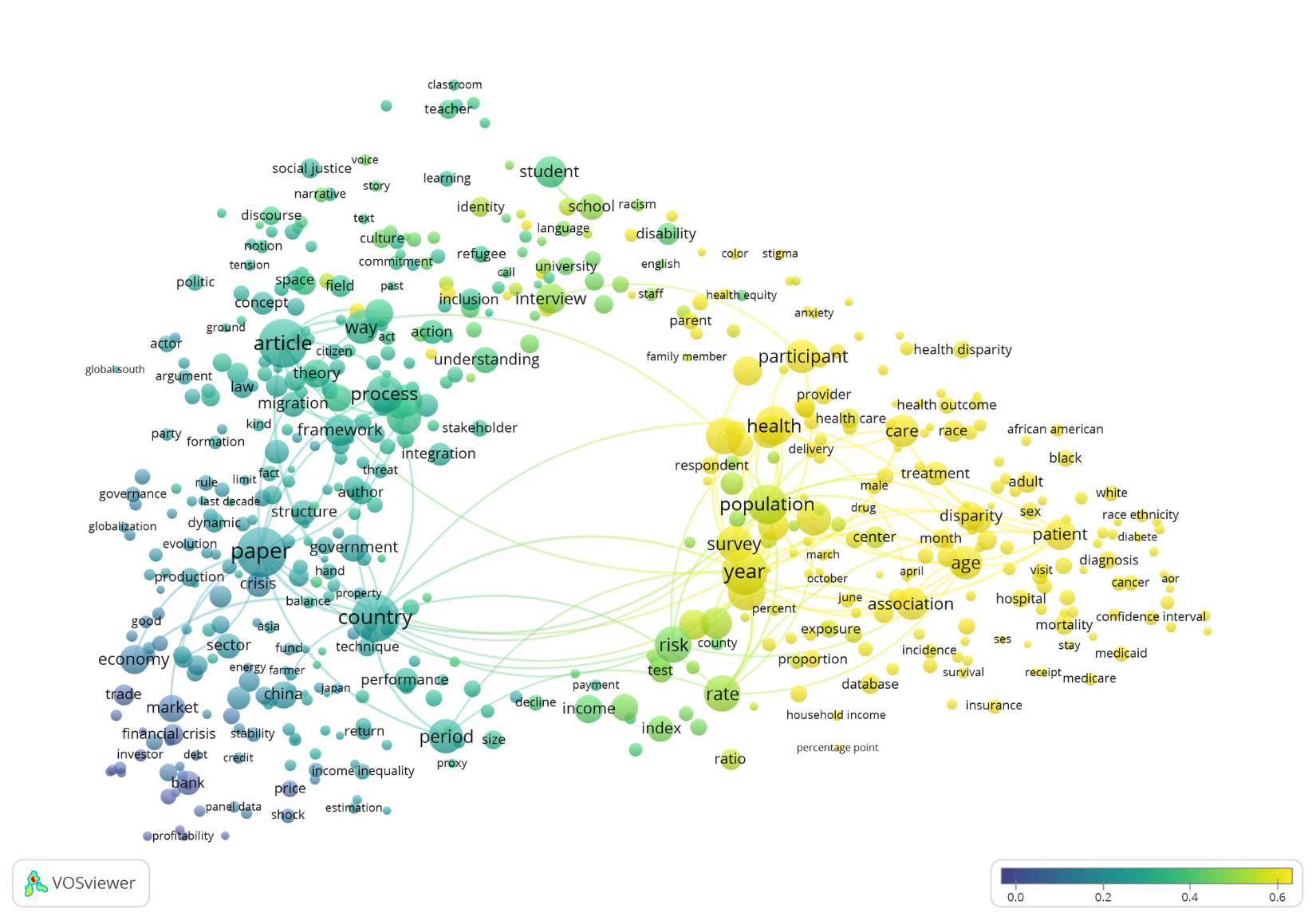


**S20 Fig. SDG 10: Reduced Inequalities term overlay map.**Binary counting (present/absent, not count of occurrences) was applied to terms in titles and abstracts of 20,025 publications in 2020 (sampled from 38,250 in total), and those with at least 100 occurrences were mapped using VOSviewer. Node size indicates count of occurrences, and node proximity reflects frequency of co-occurrence (nodes close together co-occur more frequently than nodes far apart). In this overlay visualization, the color scale indicates the proportion of publications associated with the mapped terms that were also identified by the sex and gender keyword search: blue nodes indicate terms with relatively low consideration of sex and/or gender; yellow terms indicate terms with relatively high consideration of sex and/or gender.


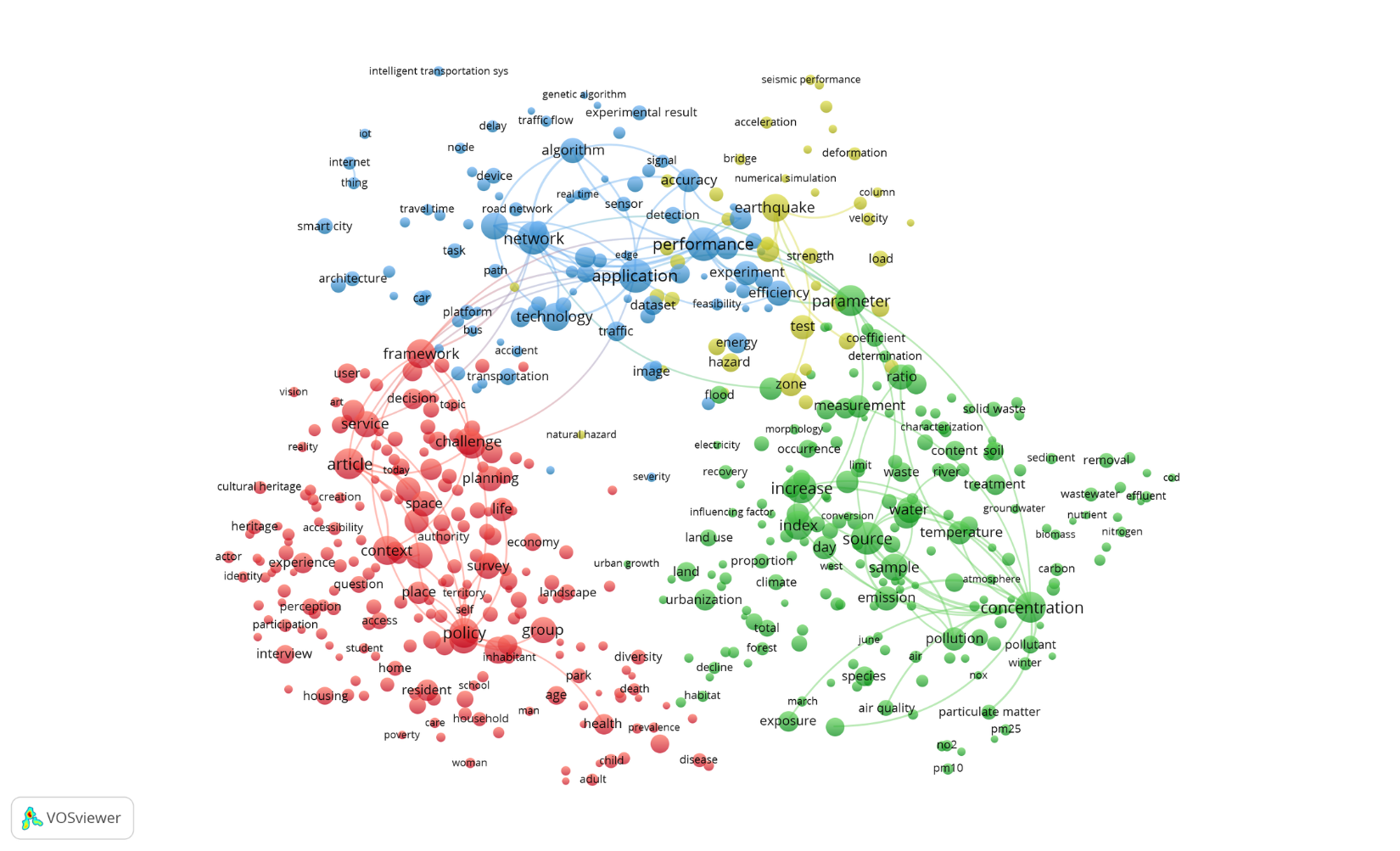


**S21 Fig. SDG 11: Sustainable Cities and Communities term network map.**Binary counting (present/absent, not count of occurrences) was applied to terms in titles and abstracts of 20,184 publications in 2020 (sampled from 57,878 in total), and those with at least 100 occurrences were mapped using VOSviewer. Node size indicates count of occurrences, and node proximity reflects frequency of co-occurrence (nodes close together co-occur more frequently than nodes far apart). In this network visualization, the colors indicate topical clusters.


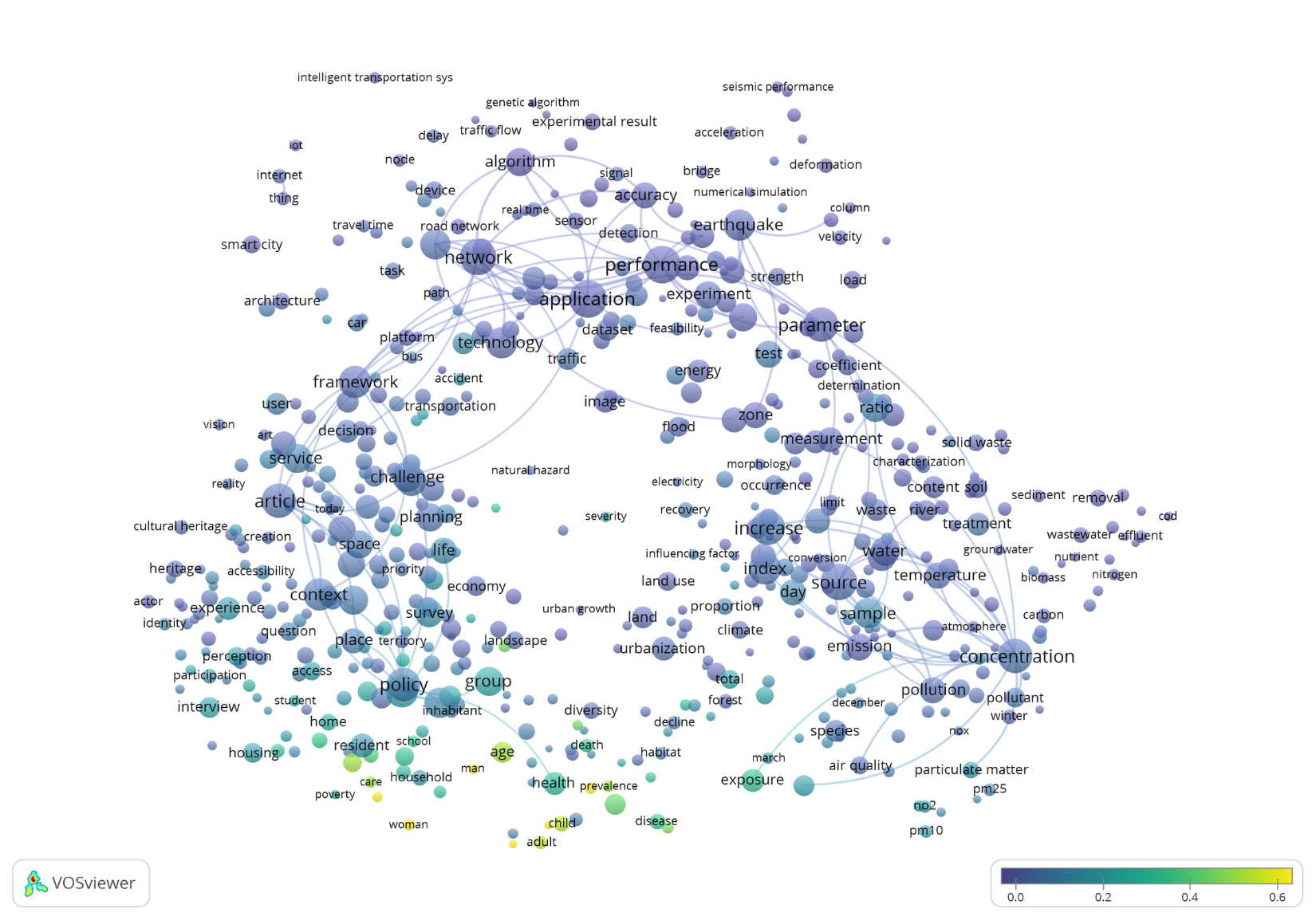


**S22 Fig. SDG 11: Sustainable Cities and Communities term overlay map.**Binary counting (present/absent, not count of occurrences) was applied to terms in titles and abstracts of 20,184 publications in 2020 (sampled from 57,878 in total), and those with at least 100 occurrences were mapped using VOSviewer. Node size indicates count of occurrences, and node proximity reflects frequency of co-occurrence (nodes close together co-occur more frequently than nodes far apart). In this overlay visualization, the color scale indicates the proportion of publications associated with the mapped terms that were also identified by the sex and gender keyword search: blue nodes indicate terms with relatively low consideration of sex and/or gender; yellow terms indicate terms with relatively high consideration of sex and/or gender.


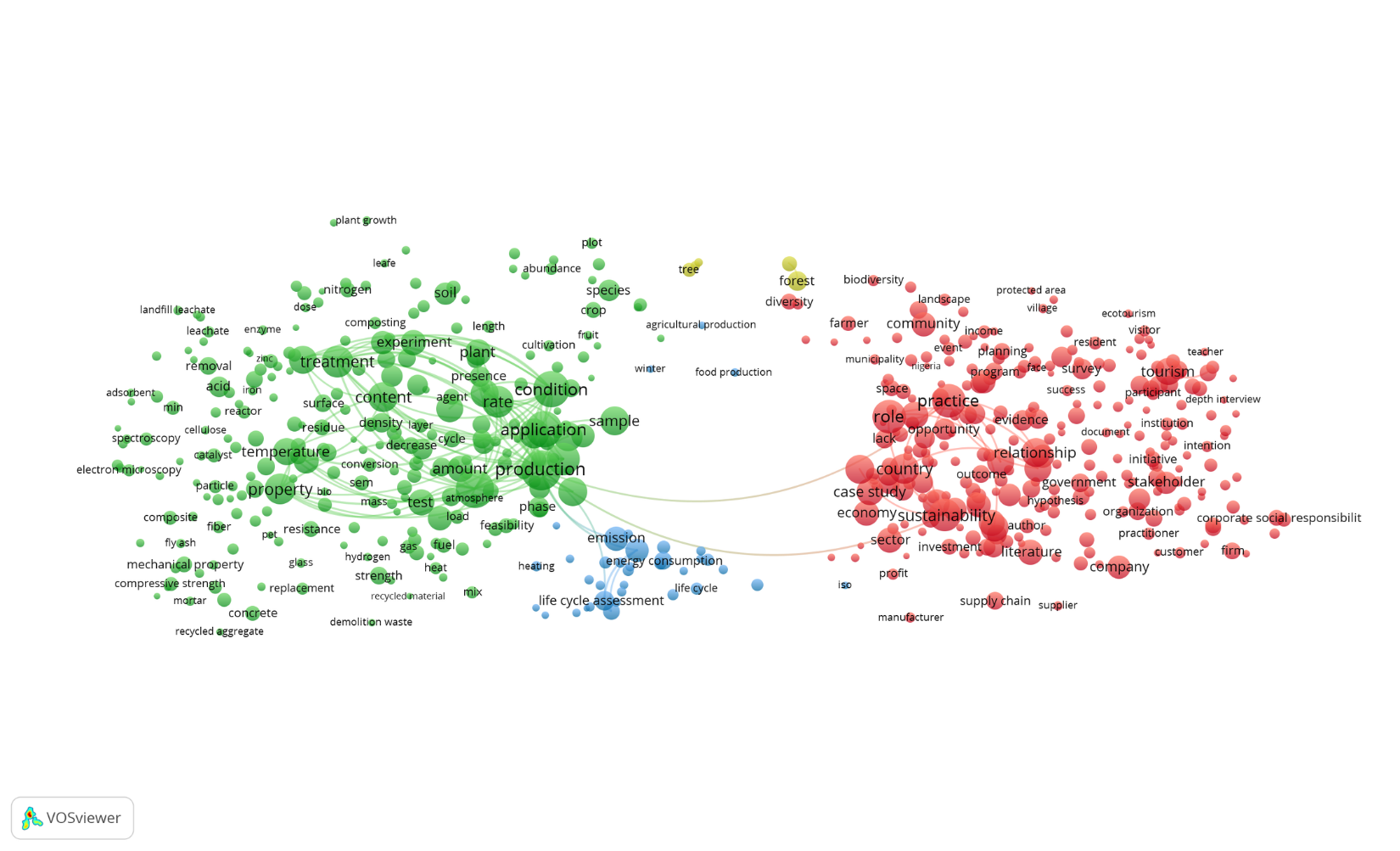


**S23 Fig. SDG 12: Responsible Consumption and Production term network map.**Binary counting (present/absent, not count of occurrences) was applied to terms in titles and abstracts of 19,984 publications in 2020 (sampled from 37,391 in total), and those with at least 100 occurrences were mapped using VOSviewer. Node size indicates count of occurrences, and node proximity reflects frequency of co-occurrence (nodes close together co-occur more frequently than nodes far apart). In this network visualization, the colors indicate topical clusters.


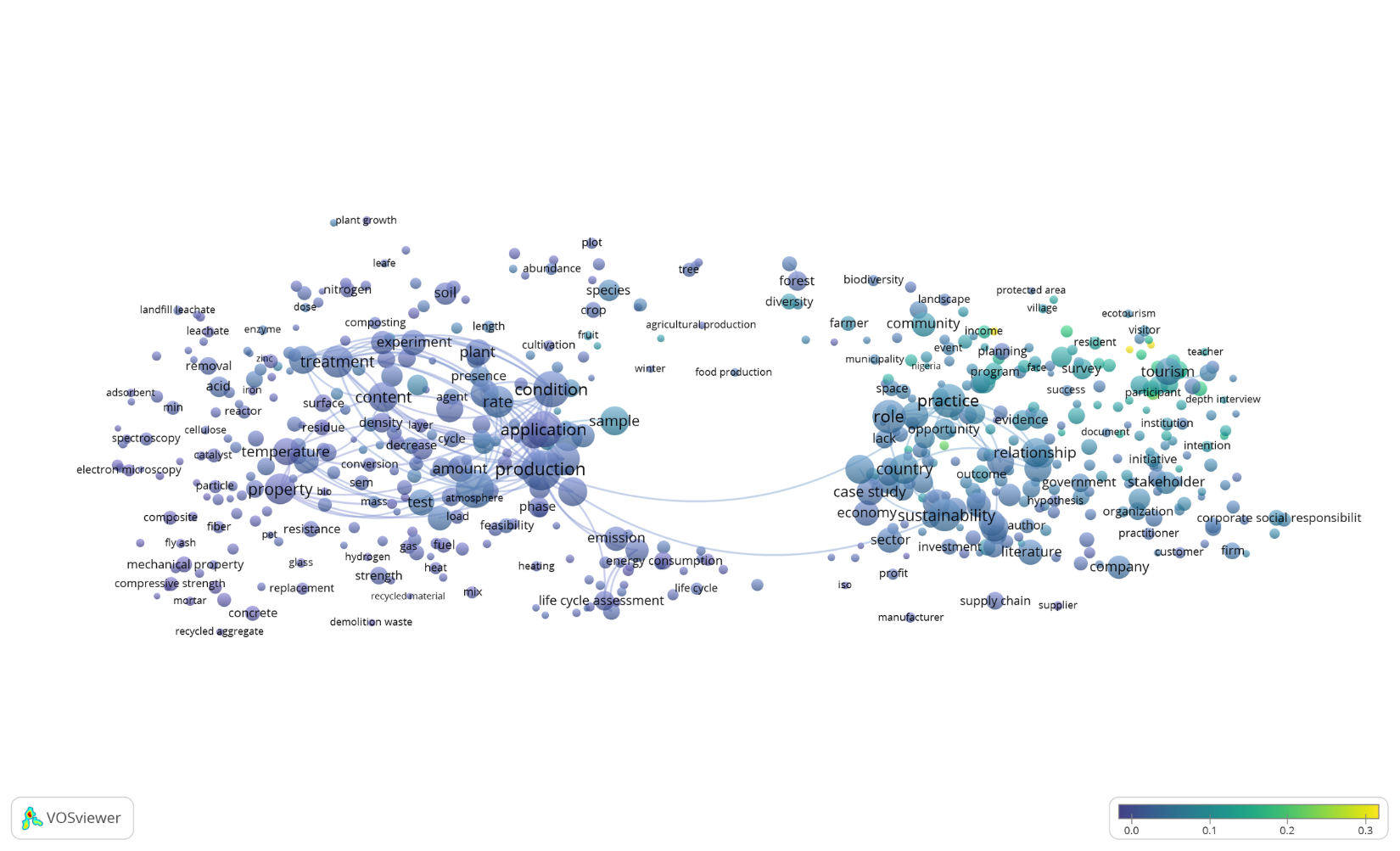


**S24 Fig. SDG 12: Responsible Consumption and Production term overlay map.**Binary counting (present/absent, not count of occurrences) was applied to terms in titles and abstracts of 19,984 publications in 2020 (sampled from 37,391 in total), and those with at least 100 occurrences were mapped using VOSviewer. Node size indicates count of occurrences, and node proximity reflects frequency of co-occurrence (nodes close together co-occur more frequently than nodes far apart). In this overlay visualization, the color scale indicates the proportion of publications associated with the mapped terms that were also identified by the sex and gender keyword search: blue nodes indicate terms with relatively low consideration of sex and/or gender; yellow terms indicate terms with relatively high consideration of sex and/or gender.


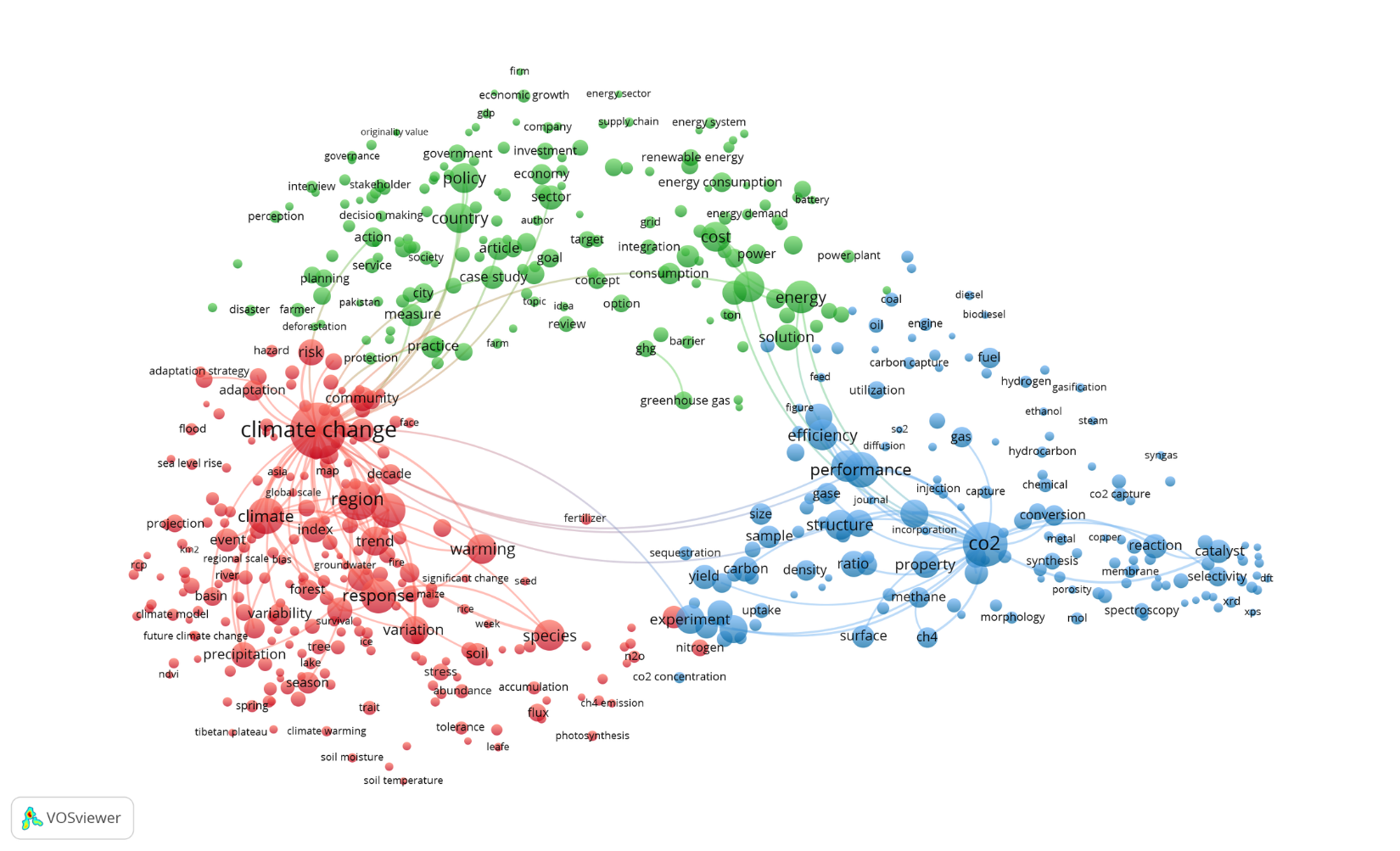


**S25 Fig. SDG 13: Climate Action term network map.**Binary counting (present/absent, not count of occurrences) was applied to terms in titles and abstracts of 20,030 publications in 2020 (sampled from 42,699 in total), and those with at least 100 occurrences were mapped using VOSviewer. Node size indicates count of occurrences, and node proximity reflects frequency of co-occurrence (nodes close together co-occur more frequently than nodes far apart). In this network visualization, the colors indicate topical clusters.


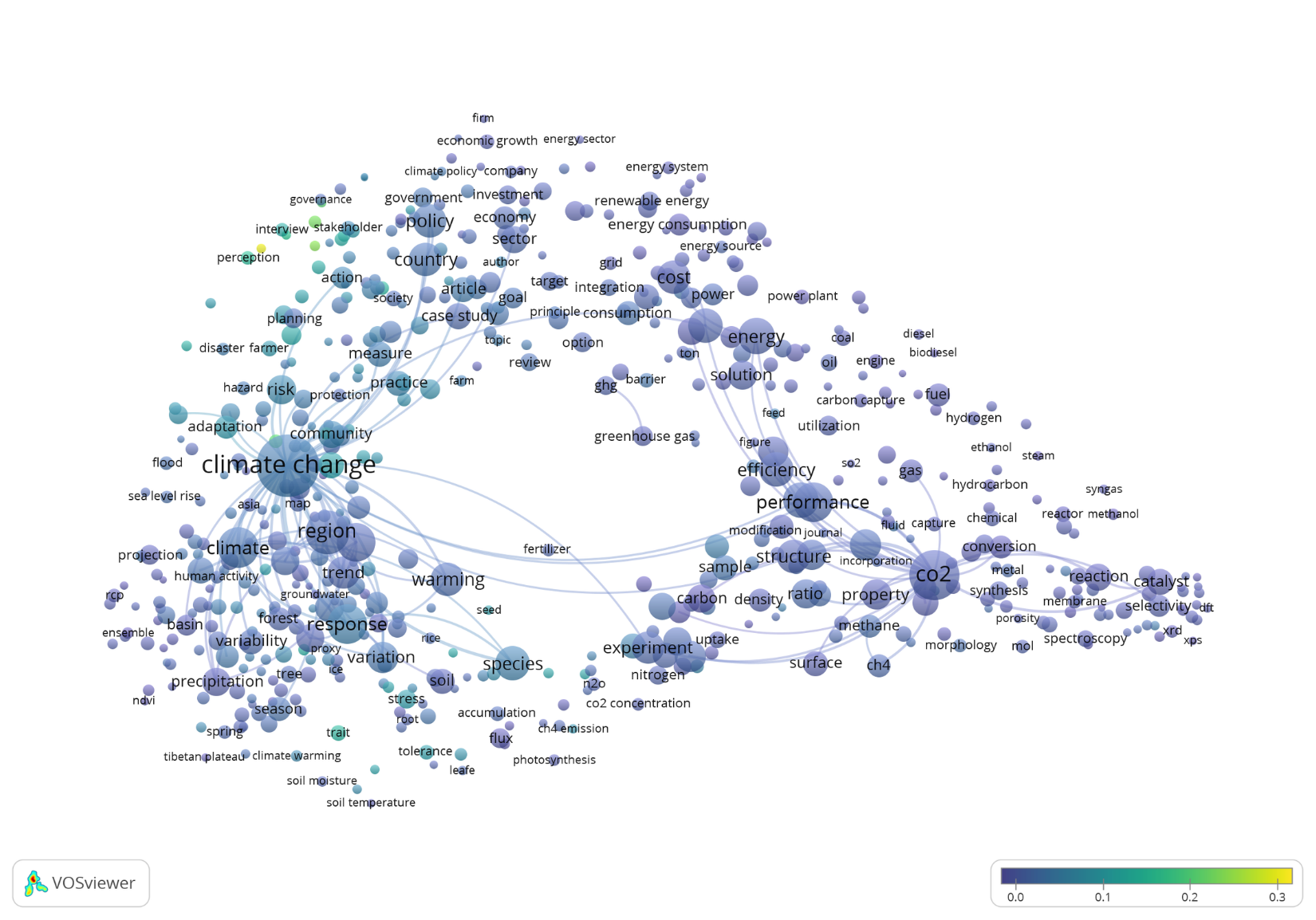


**S26 Fig. SDG 13: Climate Action term overlay map.**Binary counting (present/absent, not count of occurrences) was applied to terms in titles and abstracts of 20,030 publications in 2020 (sampled from 42,699 in total), and those with at least 100 occurrences were mapped using VOSviewer. Node size indicates count of occurrences, and node proximity reflects frequency of co-occurrence (nodes close together co-occur more frequently than nodes far apart). In this overlay visualization, the color scale indicates the proportion of publications associated with the mapped terms that were also identified by the sex and gender keyword search: blue nodes indicate terms with relatively low consideration of sex and/or gender; yellow terms indicate terms with relatively high consideration of sex and/or gender.


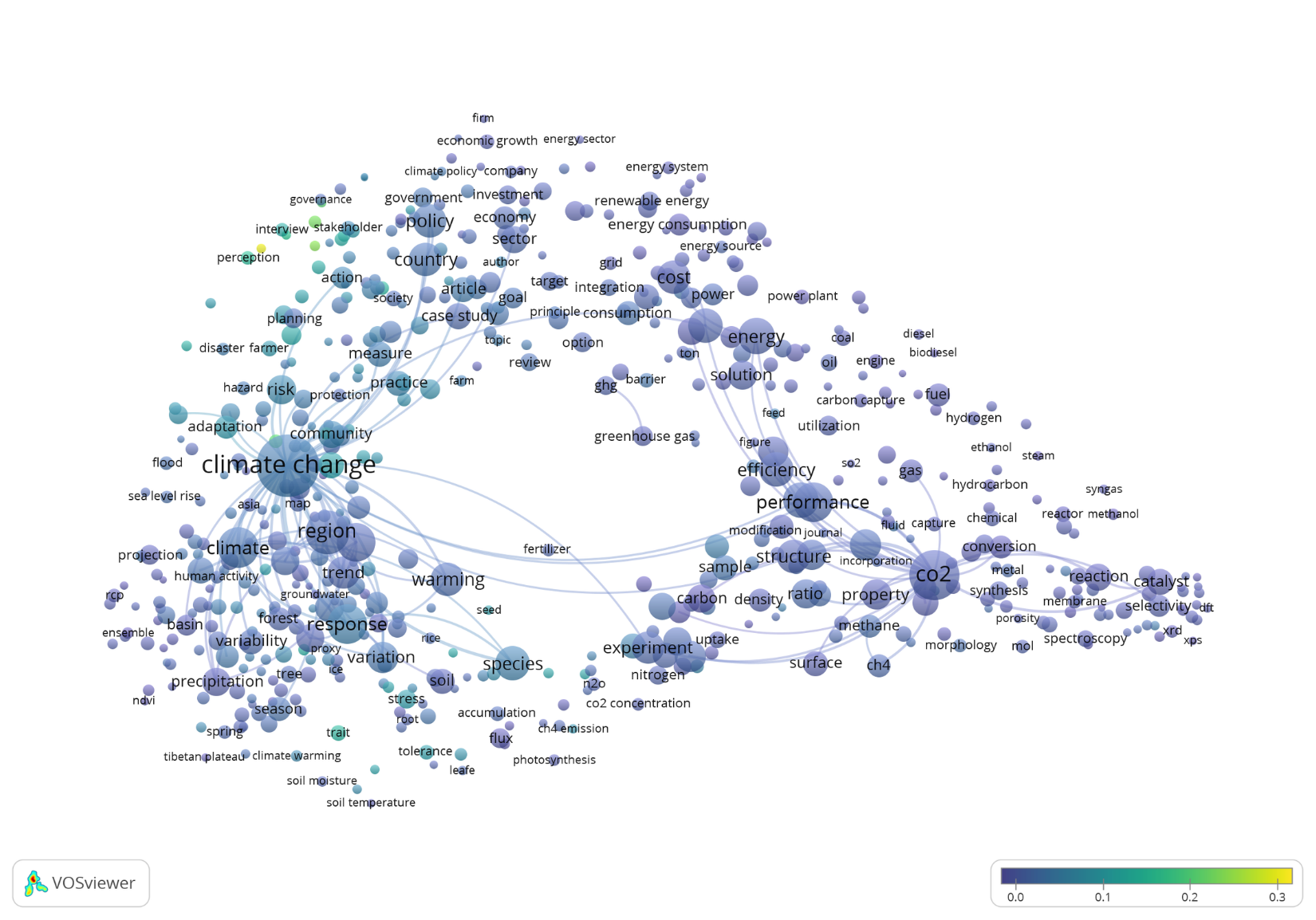


**S27 Fig. SDG 14: Life Below Water term network map.**Binary counting (present/absent, not count of occurrences) was applied to terms in titles and abstracts of 19,930 publications in 2020 (sampled from 28,146 in total), and those with at least 100 occurrences were mapped using VOSviewer. Node size indicates count of occurrences, and node proximity reflects frequency of co-occurrence (nodes close together co-occur more frequently than nodes far apart). In this network visualization, the colors indicate topical clusters.


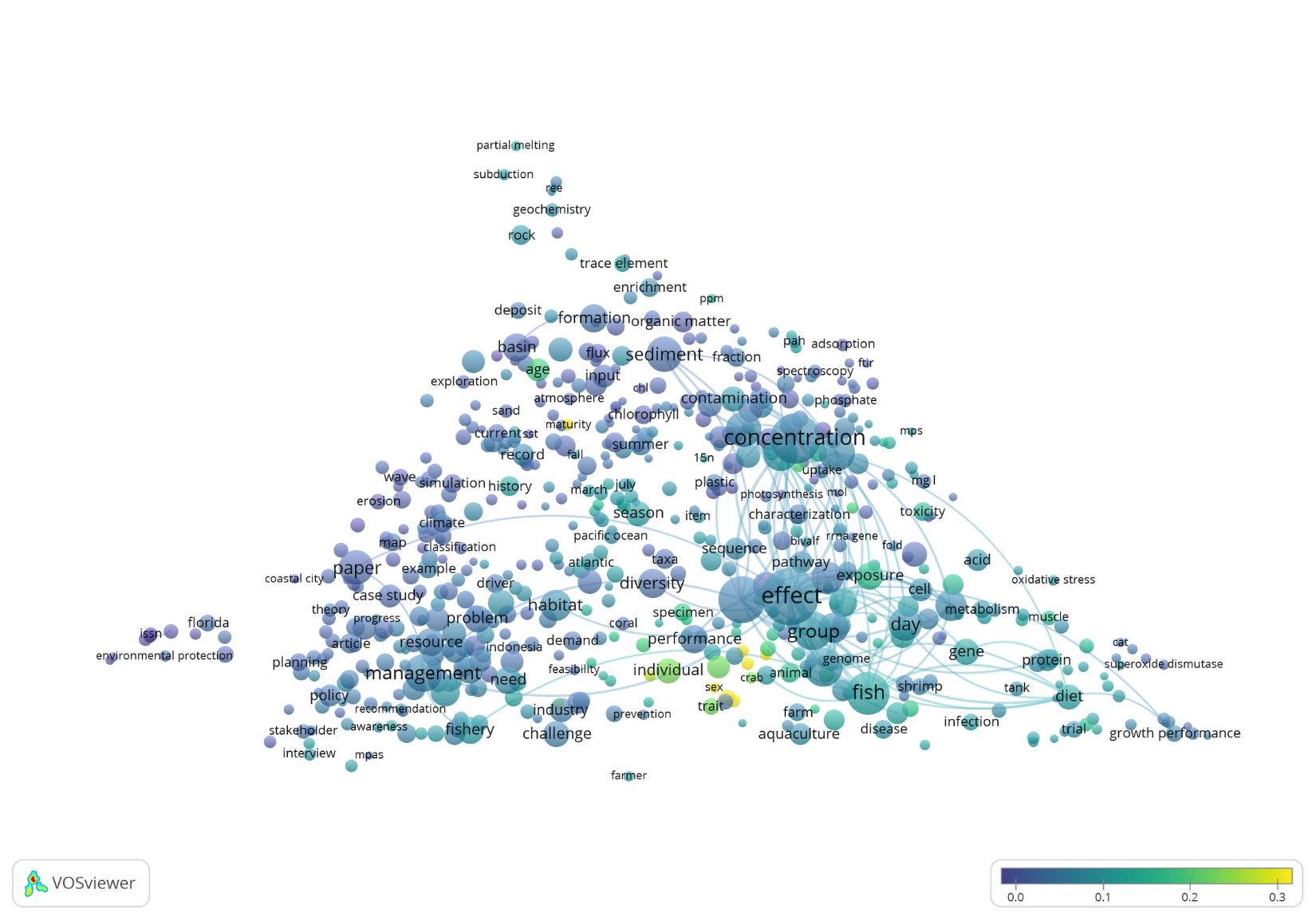


**S28 Fig. SDG 14: Life Below Water term overlay map.**Binary counting (present/absent, not count of occurrences) was applied to terms in titles and abstracts of 19,930 publications in 2020 (sampled from 28,146 in total), and those with at least 100 occurrences were mapped using VOSviewer. Node size indicates count of occurrences, and node proximity reflects frequency of co-occurrence (nodes close together co-occur more frequently than nodes far apart). In this overlay visualization, the color scale indicates the proportion of publications associated with the mapped terms that were also identified by the sex and gender keyword search: blue nodes indicate terms with relatively low consideration of sex and/or gender; yellow terms indicate terms with relatively high consideration of sex and/or gender.


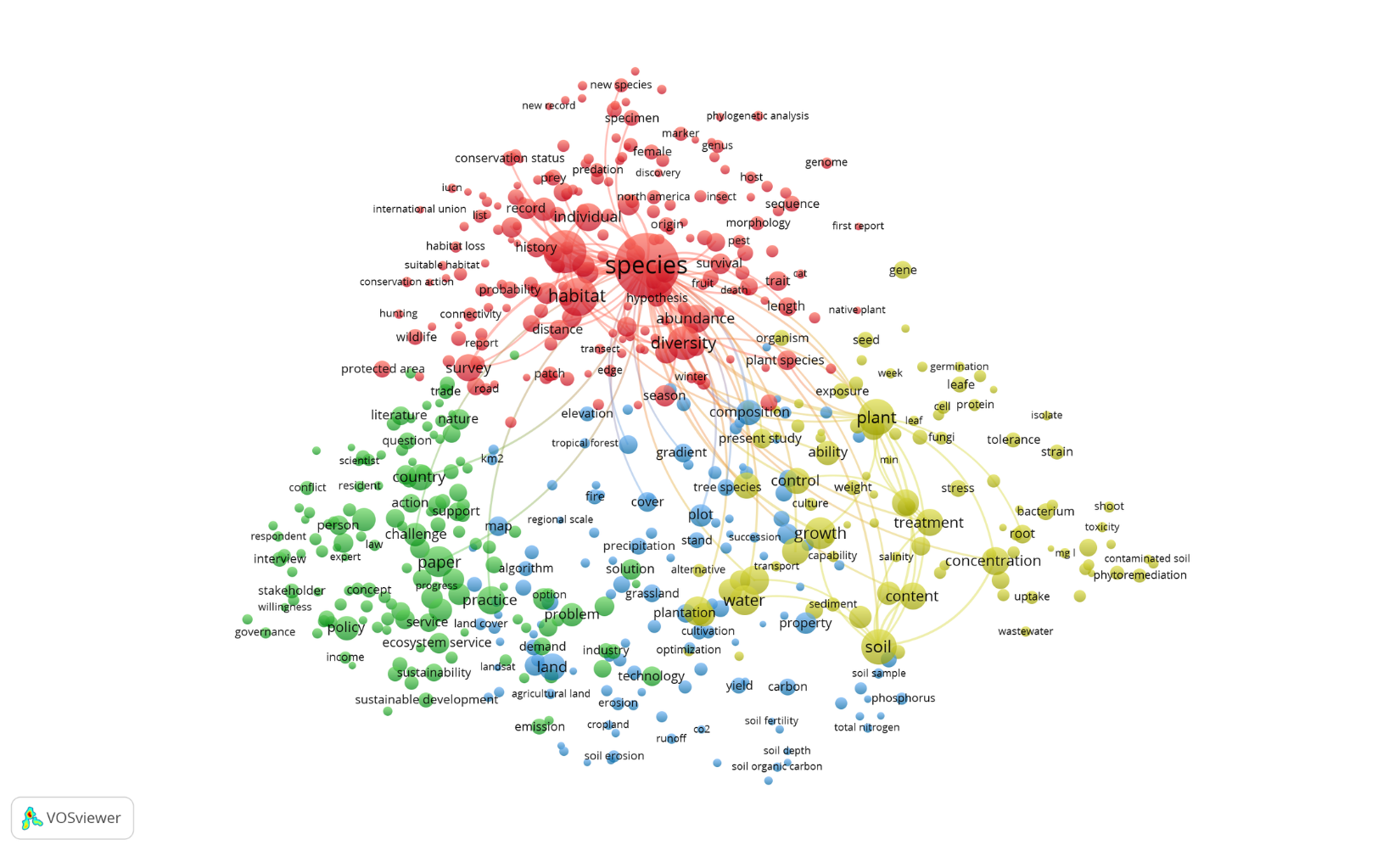


**S29 Fig. SDG 15: Life on Land term network map.**Binary counting (present/absent, not count of occurrences) was applied to terms in titles and abstracts of 19,950 publications in 2020 (sampled from 35,543 in total), and those with at least 100 occurrences were mapped using VOSviewer. Node size indicates count of occurrences, and node proximity reflects frequency of co-occurrence (nodes close together co-occur more frequently than nodes far apart). In this network visualization, the colors indicate topical clusters.


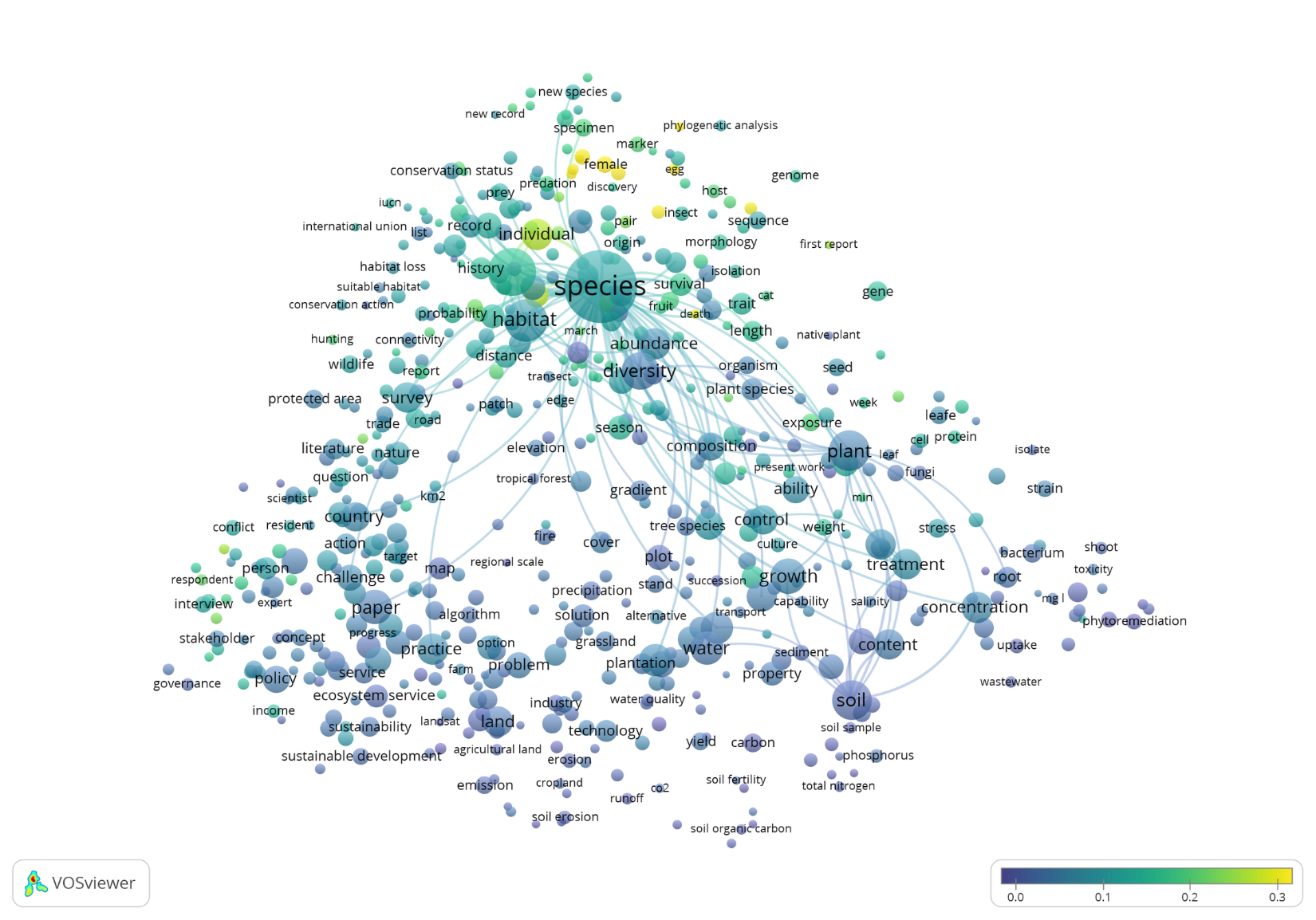


**S30 Fig. SDG 15: Life on Land term overlay map.**Binary counting (present/absent, not count of occurrences) was applied to terms in titles and abstracts of 19,950 publications in 2020 (sampled from 35,543 in total), and those with at least 100 occurrences were mapped using VOSviewer. Node size indicates count of occurrences, and node proximity reflects frequency of co-occurrence (nodes close together co-occur more frequently than nodes far apart). In this overlay visualization, the color scale indicates the proportion of publications associated with the mapped terms that were also identified by the sex and gender keyword search: blue nodes indicate terms with relatively low consideration of sex and/or gender; yellow terms indicate terms with relatively high consideration of sex and/or gender.


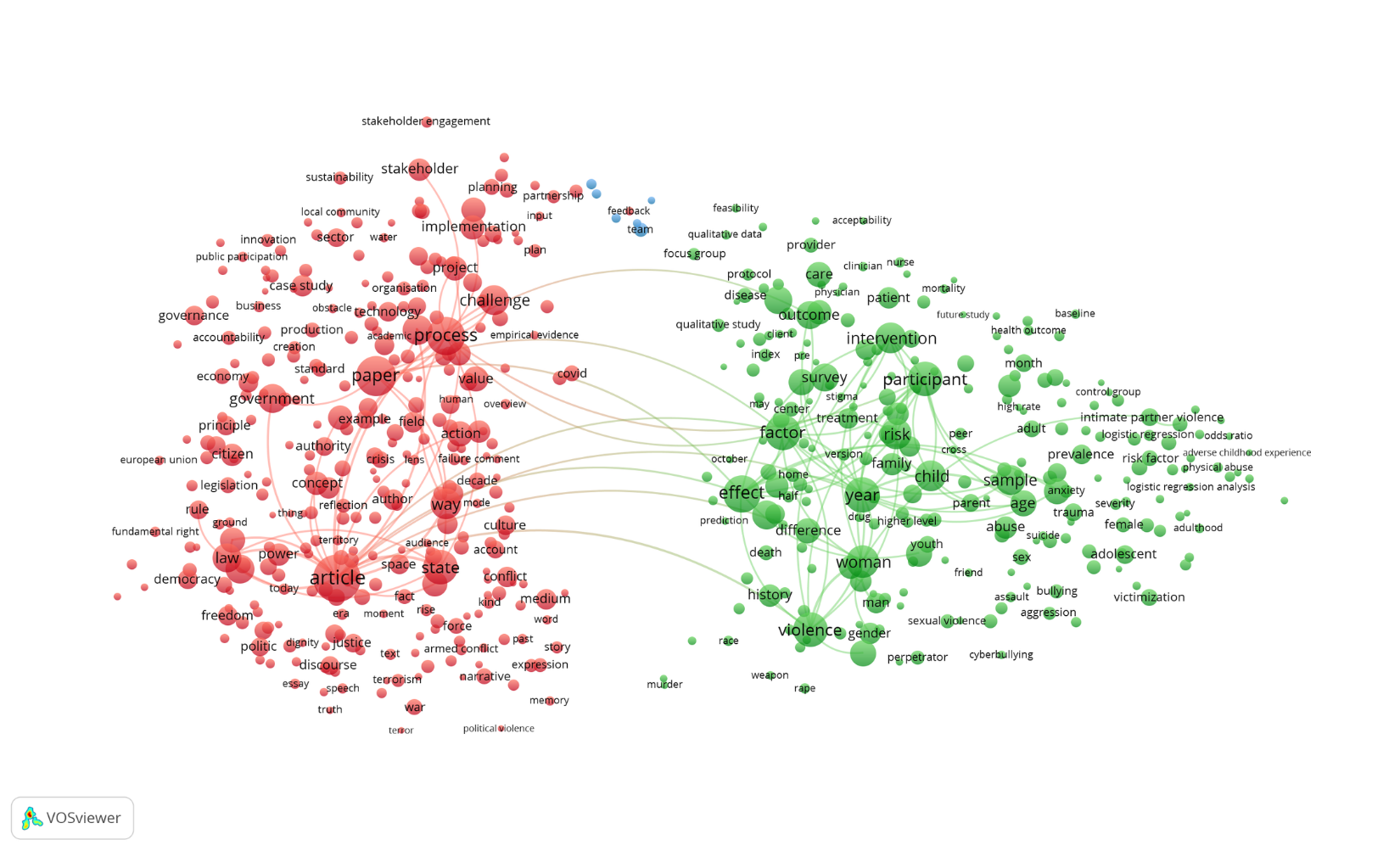


**S31 Fig. SDG 16: Peace, Justice and Strong Institutions term network map.**Binary counting (present/absent, not count of occurrences) was applied to terms in titles and abstracts of 19,975 publications in 2020 (sampled from 35,037 in total), and those with at least 100 occurrences were mapped using VOSviewer. Node size indicates count of occurrences, and node proximity reflects frequency of co-occurrence (nodes close together co-occur more frequently than nodes far apart). In this network visualization, the colors indicate topical clusters.


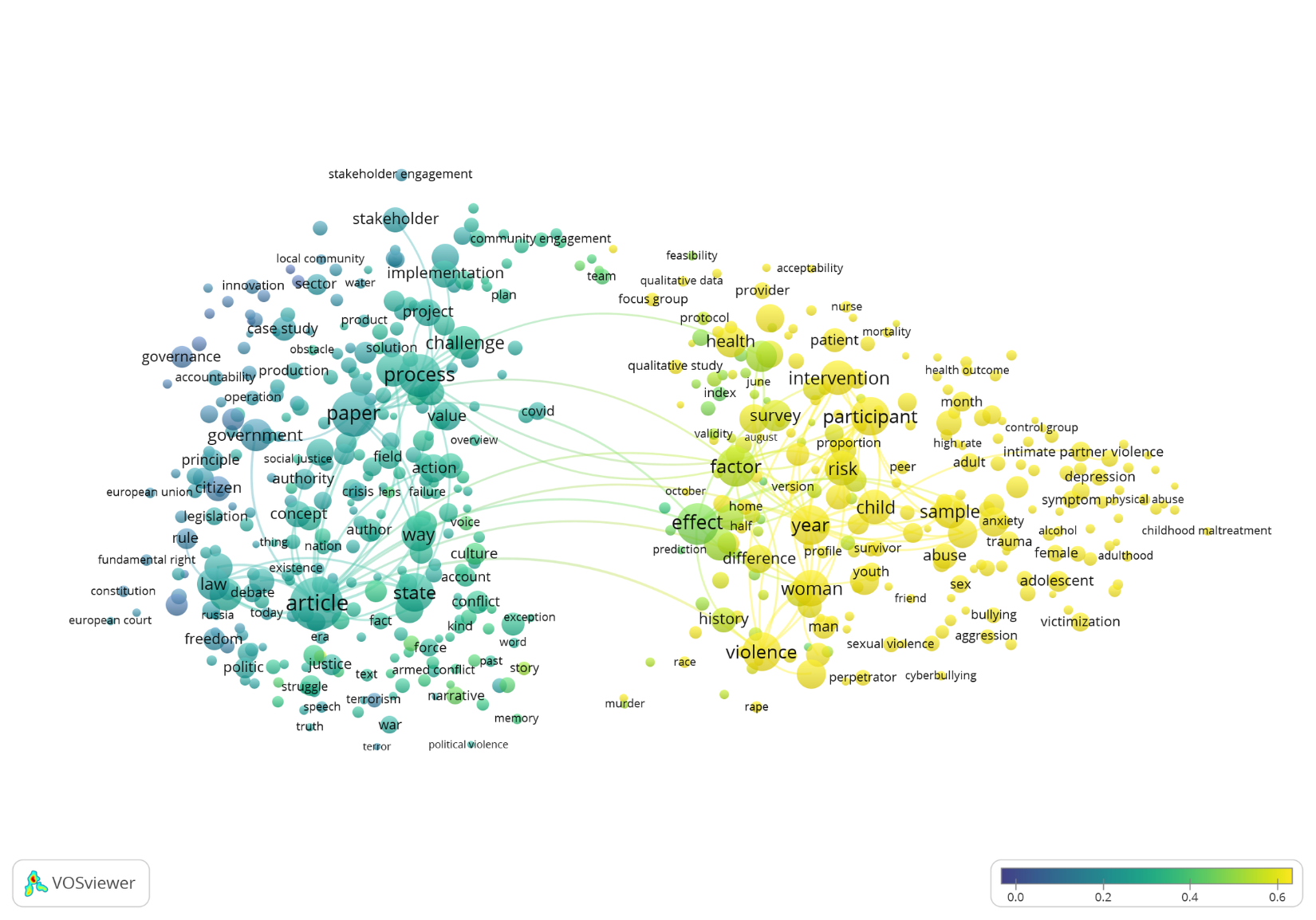


**S32 Fig. SDG 16: Peace, Justice and Strong Institutions term overlay map.**Binary counting (present/absent, not count of occurrences) was applied to terms in titles and abstracts of 19,975 publications in 2020 (sampled from 35,037 in total), and those with at least 100 occurrences were mapped using VOSviewer. Node size indicates count of occurrences, and node proximity reflects frequency of co-occurrence (nodes close together co-occur more frequently than nodes far apart). In this overlay visualization, the color scale indicates the proportion of publications associated with the mapped terms that were also identified by the sex and gender keyword search: blue nodes indicate terms with relatively low consideration of sex and/or gender; yellow terms indicate terms with relatively high consideration of sex and/or gender.
